# Supplementary material for: A novel peroxisome-related gene signature predicts clinical prognosis and is associated with immune microenvironment in low-grade glioma
Source: PeerJ. 2024 Feb 20;12:e16874. doi: 10.7717/peerj.16874 (PMC10885797; doi:10.7717/peerj.16874)
Supplement: Supplemental Information 2 [file peerj-12-16874-s002.docx]

| Table S1. 1850 DEGs between the low- and high-risk groups in the TCGA cohort were found | | | | | | |
| --- | --- | --- | --- | --- | --- | --- |
| logFC | AveExpr | t | P.Value | adj.P.Val | B |  |
| FSTL1 | 1.667346137 | 10.28515865 | 19.07613209 | 1.24E-61 | 8.10E-58 | 129.5125208 |
| PDPN | 3.261308426 | 7.784653392 | 19.05419943 | 1.58E-61 | 8.10E-58 | 129.2696218 |
| DUSP26 | -1.710235893 | 9.748191496 | -18.97248789 | 3.93E-61 | 1.61E-57 | 128.3650885 |
| ANXA1 | 2.906784634 | 9.093288187 | 18.54990091 | 4.34E-59 | 1.27E-55 | 123.6979026 |
| MSN | 1.763174615 | 11.32283556 | 18.48842851 | 8.58E-59 | 2.20E-55 | 123.0205861 |
| CRTAC1 | -2.576619189 | 9.767962533 | -18.45128982 | 1.30E-58 | 2.66E-55 | 122.6115928 |
| MAP1LC3C | 2.386491975 | 1.839399028 | 18.11874366 | 5.16E-57 | 8.15E-54 | 118.9567314 |
| TNFRSF11B | 2.651829181 | 4.126625488 | 18.09061635 | 7.04E-57 | 1.03E-53 | 118.6482273 |
| HOXA1 | 2.333630735 | 1.873792438 | 17.82392827 | 1.34E-55 | 1.71E-52 | 115.7283526 |
| PDIA4 | 1.021978773 | 10.77647592 | 17.75254292 | 2.93E-55 | 3.34E-52 | 114.9484305 |
| SLC1A6 | -2.907945904 | 6.716796418 | -17.7223639 | 4.09E-55 | 4.42E-52 | 114.6189262 |
| CSMD3 | -2.968074606 | 8.839171309 | -17.64278128 | 9.81E-55 | 1.01E-51 | 113.7506458 |
| ATP6V1G2 | -1.638114417 | 11.02574695 | -17.63295697 | 1.09E-54 | 1.02E-51 | 113.6435223 |
| LRIT2 | -2.181097737 | 4.383479815 | -17.56874169 | 2.21E-54 | 1.89E-51 | 112.9436751 |
| RIPPLY2 | -1.708149247 | 5.215571138 | -17.55249527 | 2.64E-54 | 2.17E-51 | 112.7667115 |
| CBLN1 | -2.74548113 | 6.451011006 | -17.52957252 | 3.40E-54 | 2.68E-51 | 112.5170935 |
| HOXC10 | 3.169839338 | 1.268711241 | 17.42355765 | 1.09E-53 | 8.27E-51 | 111.3636813 |
| CD58 | 1.543983389 | 6.090230366 | 17.24317754 | 7.83E-53 | 5.54E-50 | 109.4052329 |
| GABBR1 | -1.611162649 | 13.13350789 | -17.19666653 | 1.30E-52 | 8.91E-50 | 108.9010956 |
| FNDC3B | 1.020209427 | 8.480245601 | 17.18086812 | 1.55E-52 | 1.02E-49 | 108.7299359 |
| SEMA4G | -1.080248935 | 8.192517555 | -17.14841292 | 2.20E-52 | 1.33E-49 | 108.3784464 |
| PRLHR | -4.099619392 | 5.722896212 | -17.12362792 | 2.89E-52 | 1.69E-49 | 108.1101432 |
| CRY2 | -1.098401687 | 10.88685905 | -16.94917839 | 1.93E-51 | 1.07E-48 | 106.224637 |
| FAM133A | -2.180620398 | 7.000250934 | -16.93106022 | 2.35E-51 | 1.26E-48 | 106.0291115 |
| SMC4 | 1.371377719 | 8.542063703 | 16.92989761 | 2.38E-51 | 1.26E-48 | 106.0165669 |
| HOXC9 | 2.24995822 | 0.830205519 | 16.92298011 | 2.57E-51 | 1.32E-48 | 105.941932 |
| RASL10A | -2.451204914 | 8.493959763 | -16.86612732 | 4.77E-51 | 2.39E-48 | 105.3288518 |
| STEAP3 | 2.197521012 | 7.773074454 | 16.83453636 | 6.72E-51 | 3.21E-48 | 104.9884349 |
| FAM57B | -1.797118711 | 9.192018903 | -16.82565815 | 7.40E-51 | 3.45E-48 | 104.8927976 |
| DNAJC12 | -1.168374329 | 8.453626843 | -16.8218771 | 7.71E-51 | 3.52E-48 | 104.852072 |
| SERP2 | -1.014736244 | 8.679155027 | -16.80080652 | 9.69E-51 | 4.33E-48 | 104.6251681 |
| CHI3L1 | 4.319315974 | 8.786042712 | 16.77953282 | 1.22E-50 | 5.33E-48 | 104.3961587 |
| IGFBP2 | 2.702214592 | 8.490055981 | 16.73563012 | 1.97E-50 | 8.41E-48 | 103.9238113 |
| CCDC109B | 1.820536682 | 6.071924827 | 16.72129627 | 2.30E-50 | 9.62E-48 | 103.7696706 |
| OTP | 2.701588445 | 1.179310251 | 16.6842103 | 3.43E-50 | 1.38E-47 | 103.371039 |
| EVC2 | 2.260494053 | 3.424821294 | 16.67997307 | 3.59E-50 | 1.42E-47 | 103.3255099 |
| HOXA5 | 2.950543921 | 1.323558451 | 16.66922088 | 4.04E-50 | 1.56E-47 | 103.2099926 |
| TMEM71 | 2.080940967 | 3.79781967 | 16.66474349 | 4.24E-50 | 1.61E-47 | 103.1618956 |
| CHRNA4 | -2.291752752 | 8.298373423 | -16.65090877 | 4.92E-50 | 1.84E-47 | 103.0133041 |
| PTGFRN | 1.43632516 | 9.650430365 | 16.64612063 | 5.18E-50 | 1.90E-47 | 102.9618856 |
| SHOX2 | 3.581144132 | 2.309168609 | 16.60249343 | 8.31E-50 | 2.94E-47 | 102.4935842 |
| ZDHHC22 | -1.998084266 | 11.08884148 | -16.58504496 | 1.00E-49 | 3.49E-47 | 102.3063905 |
| NOG | -2.20183932 | 7.63310586 | -16.57588695 | 1.11E-49 | 3.79E-47 | 102.2081631 |
| CDHR1 | -2.024015803 | 9.595157049 | -16.55357798 | 1.41E-49 | 4.75E-47 | 101.968948 |
| PCBP3 | -1.86145381 | 7.836917104 | -16.52097501 | 2.01E-49 | 6.64E-47 | 101.619524 |
| EMP3 | 2.78156681 | 6.633537672 | 16.44726222 | 4.45E-49 | 1.45E-46 | 100.8302642 |
| CPEB3 | -1.151593361 | 8.266156736 | -16.35469529 | 1.21E-48 | 3.75E-46 | 99.84064565 |
| LOC339674 | -1.465680464 | 8.81679232 | -16.29751561 | 2.23E-48 | 6.84E-46 | 99.23020458 |
| NTNG2 | -1.469206197 | 9.169175781 | -16.28574767 | 2.53E-48 | 7.64E-46 | 99.10465426 |
| GPX8 | 2.316458126 | 4.470389963 | 16.26987401 | 3.00E-48 | 8.94E-46 | 98.93534545 |
| TIMP1 | 2.642144604 | 8.37767555 | 16.2487226 | 3.77E-48 | 1.11E-45 | 98.7098242 |
| SH2D4A | 2.325632144 | 3.601987118 | 16.24590453 | 3.89E-48 | 1.12E-45 | 98.67978412 |
| CASP8 | 1.311527298 | 6.246384855 | 16.24225136 | 4.04E-48 | 1.14E-45 | 98.64084464 |
| PLSCR1 | 1.455928502 | 8.259898628 | 16.23489457 | 4.37E-48 | 1.21E-45 | 98.56243616 |
| C17orf96 | -1.400756657 | 8.272907616 | -16.21400101 | 5.47E-48 | 1.50E-45 | 98.33981419 |
| ZNF217 | 1.147378252 | 7.89852083 | 16.21206824 | 5.59E-48 | 1.50E-45 | 98.31922499 |
| ADAM12 | 2.236058688 | 5.454569828 | 16.15504508 | 1.03E-47 | 2.71E-45 | 97.71212499 |
| STARD10 | -1.0127876 | 9.153623688 | -16.15158964 | 1.07E-47 | 2.78E-45 | 97.67535837 |
| GALNT3 | 1.975738618 | 3.870698374 | 16.13695249 | 1.25E-47 | 3.17E-45 | 97.51964357 |
| SNAP91 | -2.232673262 | 10.34567523 | -16.10323596 | 1.80E-47 | 4.50E-45 | 97.16112828 |
| SGSM1 | -1.961319554 | 8.514127981 | -16.09709112 | 1.92E-47 | 4.75E-45 | 97.09581479 |
| PHYHIPL | -1.281652956 | 12.54125593 | -16.04414806 | 3.38E-47 | 8.17E-45 | 96.53341692 |
| SAMD9L | 1.604339305 | 8.414081761 | 16.01655351 | 4.55E-47 | 1.09E-44 | 96.24052711 |
| GNAL | -2.028769067 | 7.963509565 | -16.00263319 | 5.28E-47 | 1.24E-44 | 96.09283857 |
| KCNIP2 | -2.409383936 | 9.375175375 | -15.98288005 | 6.52E-47 | 1.52E-44 | 95.88333865 |
| JPH3 | -2.351452503 | 9.849633034 | -15.96967598 | 7.50E-47 | 1.73E-44 | 95.74334491 |
| TGFB2 | 2.261198958 | 8.788792052 | 15.96763723 | 7.67E-47 | 1.75E-44 | 95.72173288 |
| HOXA6 | 1.898493099 | 0.637796501 | 15.96167015 | 8.17E-47 | 1.84E-44 | 95.65848316 |
| DPYD | 1.64938754 | 7.057749194 | 15.92286327 | 1.24E-46 | 2.76E-44 | 95.24732956 |
| DPY19L1 | 1.074722639 | 10.17758675 | 15.91891435 | 1.29E-46 | 2.85E-44 | 95.20550987 |
| FAM155A | -1.924160283 | 9.094356347 | -15.89921946 | 1.59E-46 | 3.44E-44 | 94.99698931 |
| NAP1L3 | -1.098158157 | 10.9110706 | -15.87545047 | 2.05E-46 | 4.39E-44 | 94.74544872 |
| PYGL | 1.480874301 | 8.584191939 | 15.85885332 | 2.45E-46 | 5.18E-44 | 94.56988031 |
| CA3 | 2.64908414 | 4.174409759 | 15.83276387 | 3.24E-46 | 6.71E-44 | 94.29402496 |
| SHISA7 | -1.851921269 | 9.535952241 | -15.82190315 | 3.63E-46 | 7.46E-44 | 94.17923477 |
| WNT7B | -2.732288663 | 8.193096471 | -15.78918845 | 5.15E-46 | 1.05E-43 | 93.83362431 |
| SP100 | 1.375362147 | 8.20350914 | 15.76645827 | 6.55E-46 | 1.32E-43 | 93.59363707 |
| HOXA4 | 2.971865139 | 1.568497359 | 15.72427218 | 1.03E-45 | 2.05E-43 | 93.14854537 |
| PAK7 | -2.15832436 | 7.249127763 | -15.70636571 | 1.24E-45 | 2.45E-43 | 92.95974353 |
| HOXB3 | 2.938922593 | 2.593204019 | 15.66906742 | 1.85E-45 | 3.54E-43 | 92.56671687 |
| HOXA3 | 2.913852394 | 1.266293785 | 15.66816015 | 1.86E-45 | 3.54E-43 | 92.55716065 |
| FAM111A | 1.10090815 | 8.552075338 | 15.65446378 | 2.16E-45 | 4.06E-43 | 92.41292058 |
| IGF2BP3 | 2.437398443 | 4.490406295 | 15.65011505 | 2.26E-45 | 4.21E-43 | 92.36713219 |
| ADAMTSL4 | 1.509592743 | 5.555706784 | 15.6450341 | 2.38E-45 | 4.41E-43 | 92.31363968 |
| ZC3H12B | -1.574964561 | 7.421842556 | -15.61434203 | 3.30E-45 | 6.00E-43 | 91.99064053 |
| CLIC1 | 1.513183248 | 9.465450218 | 15.60051329 | 3.82E-45 | 6.88E-43 | 91.84518106 |
| MDGA2 | -2.148950821 | 7.37407223 | -15.59967074 | 3.86E-45 | 6.88E-43 | 91.83632011 |
| KIAA1543 | -2.075662871 | 7.907227964 | -15.59624201 | 4.00E-45 | 7.08E-43 | 91.8002622 |
| CD101 | 1.623757672 | 4.724874035 | 15.56235579 | 5.73E-45 | 9.96E-43 | 91.4440509 |
| ABCC3 | 3.463150759 | 4.770802601 | 15.50857184 | 1.01E-44 | 1.75E-42 | 90.87923633 |
| COL5A2 | 1.854754494 | 8.123570066 | 15.49107692 | 1.22E-44 | 2.07E-42 | 90.6956622 |
| SLITRK5 | -1.474668281 | 8.698203903 | -15.48649117 | 1.28E-44 | 2.15E-42 | 90.64755613 |
| SHANK2 | -2.179375299 | 8.850989582 | -15.46887052 | 1.54E-44 | 2.57E-42 | 90.46275694 |
| IQGAP1 | 1.276786588 | 10.24095093 | 15.46207193 | 1.66E-44 | 2.74E-42 | 90.39147581 |
| TGIF1 | 1.446220041 | 8.025303575 | 15.44538308 | 1.97E-44 | 3.22E-42 | 90.21654586 |
| GABRB3 | -2.177863858 | 10.03355106 | -15.43060277 | 2.31E-44 | 3.70E-42 | 90.06167747 |
| SERPINH1 | 1.492353082 | 9.117630661 | 15.40865375 | 2.91E-44 | 4.63E-42 | 89.83179379 |
| NSUN7 | 2.002977759 | 3.426618075 | 15.39018497 | 3.54E-44 | 5.54E-42 | 89.63845207 |
| EN1 | 3.271356471 | 2.386282642 | 15.37582763 | 4.12E-44 | 6.40E-42 | 89.48820916 |
| TUBA1C | 1.559783648 | 8.489498635 | 15.35647466 | 5.05E-44 | 7.76E-42 | 89.28576987 |
| FKBP9 | 1.369310754 | 9.694859956 | 15.35607944 | 5.07E-44 | 7.76E-42 | 89.2816367 |
| CACNA2D2 | -1.685397239 | 7.481200778 | -15.32090847 | 7.34E-44 | 1.12E-41 | 88.91397661 |
| H2AFY2 | -1.664701615 | 9.117816427 | -15.27774244 | 1.16E-43 | 1.73E-41 | 88.46316212 |
| MRC2 | 1.595056982 | 10.0346356 | 15.24929194 | 1.56E-43 | 2.31E-41 | 88.16628873 |
| NDRG2 | -1.262383336 | 14.84721128 | -15.247766 | 1.59E-43 | 2.33E-41 | 88.15037178 |
| TUB | -1.15857686 | 11.32483917 | -15.24104933 | 1.70E-43 | 2.48E-41 | 88.08031756 |
| TMEM154 | 1.459293628 | 4.515938125 | 15.22364904 | 2.04E-43 | 2.95E-41 | 87.89888751 |
| NCRNA00219 | -1.04389294 | 8.961057672 | -15.22222637 | 2.07E-43 | 2.98E-41 | 87.88405698 |
| SCRT1 | -2.872369092 | 8.088158025 | -15.21921368 | 2.14E-43 | 3.05E-41 | 87.852653 |
| PLCB1 | -1.354255008 | 10.41421132 | -15.11308528 | 6.52E-43 | 9.17E-41 | 86.74786674 |
| BMP2 | -2.15307765 | 9.771275365 | -15.10598576 | 7.03E-43 | 9.75E-41 | 86.67406522 |
| JPH4 | -1.71514928 | 10.99912271 | -15.07921153 | 9.30E-43 | 1.27E-40 | 86.39585759 |
| VAV3 | 2.557950755 | 5.106034519 | 15.06833492 | 1.04E-42 | 1.42E-40 | 86.28289361 |
| METTL7B | 2.631960517 | 7.133529484 | 15.06392771 | 1.09E-42 | 1.47E-40 | 86.23712929 |
| PLAT | 1.940172562 | 8.137464497 | 15.06008469 | 1.14E-42 | 1.53E-40 | 86.19722773 |
| TNFRSF12A | 2.310691092 | 6.975069144 | 15.05157004 | 1.24E-42 | 1.66E-40 | 86.10883503 |
| STAC | 2.730645347 | 3.086344228 | 15.04830157 | 1.29E-42 | 1.69E-40 | 86.0749093 |
| PRICKLE3 | 1.297106131 | 4.176272306 | 15.04549911 | 1.32E-42 | 1.73E-40 | 86.04582285 |
| TTC26 | 1.271747572 | 5.656806091 | 15.03381206 | 1.50E-42 | 1.94E-40 | 85.92454644 |
| FAM109B | 1.025988201 | 5.536411459 | 15.02687093 | 1.61E-42 | 2.06E-40 | 85.8525354 |
| HOXC6 | 2.278408796 | 1.205285796 | 15.01602505 | 1.80E-42 | 2.28E-40 | 85.74003987 |
| MACC1 | 1.870107916 | 2.228443606 | 15.00923691 | 1.94E-42 | 2.44E-40 | 85.66964784 |
| TAGLN2 | 1.505553987 | 10.32432559 | 15.00728576 | 1.98E-42 | 2.47E-40 | 85.64941694 |
| LOC647946 | 1.964172586 | 1.14367273 | 15.00599957 | 2.00E-42 | 2.49E-40 | 85.63608138 |
| SPHKAP | -3.02950655 | 8.30481375 | -14.99038999 | 2.36E-42 | 2.91E-40 | 85.47427213 |
| RUNDC3A | -1.527258705 | 11.01176108 | -14.96162984 | 3.18E-42 | 3.89E-40 | 85.1763135 |
| RAB34 | 1.661429187 | 7.568094486 | 14.94871831 | 3.64E-42 | 4.42E-40 | 85.04262037 |
| GDAP1L1 | -1.91452531 | 9.667015676 | -14.9455386 | 3.77E-42 | 4.55E-40 | 85.00970275 |
| HOXD11 | 2.418783817 | 0.911303898 | 14.93991123 | 3.99E-42 | 4.79E-40 | 84.9514526 |
| HOXC11 | 1.824759643 | 0.538366033 | 14.91166618 | 5.36E-42 | 6.36E-40 | 84.65921057 |
| SERPING1 | 1.871246226 | 9.710548869 | 14.91034661 | 5.44E-42 | 6.41E-40 | 84.64556268 |
| ANXA2P2 | 1.986458965 | 7.895013594 | 14.90516074 | 5.74E-42 | 6.69E-40 | 84.59193151 |
| NALCN | -1.186682047 | 9.956874666 | -14.8164977 | 1.45E-41 | 1.60E-39 | 83.67612658 |
| ANXA2 | 1.773410137 | 9.603518451 | 14.79636003 | 1.78E-41 | 1.94E-39 | 83.46842337 |
| HOXD13 | 2.564062071 | 1.029397759 | 14.7907655 | 1.89E-41 | 2.04E-39 | 83.41074033 |
| HOTAIR | 1.983128852 | 0.591383889 | 14.78528578 | 2.00E-41 | 2.15E-39 | 83.3542494 |
| HOXA2 | 2.49303186 | 0.964902892 | 14.78322955 | 2.04E-41 | 2.18E-39 | 83.33305361 |
| HOXA7 | 3.64699195 | 2.259363773 | 14.75874657 | 2.63E-41 | 2.79E-39 | 83.08077192 |
| AFF2 | -2.305067836 | 7.796326431 | -14.74889821 | 2.92E-41 | 3.06E-39 | 82.9793376 |
| GALNT5 | 2.16534398 | 0.995770642 | 14.70911783 | 4.41E-41 | 4.57E-39 | 82.56989044 |
| RCOR2 | -1.921456919 | 9.645713516 | -14.69659304 | 5.02E-41 | 5.18E-39 | 82.44106834 |
| LAMC1 | 1.200077059 | 9.856097017 | 14.68236367 | 5.82E-41 | 5.92E-39 | 82.29476745 |
| TEF | -1.047533067 | 10.57394032 | -14.64389699 | 8.67E-41 | 8.64E-39 | 81.89955386 |
| GALNT13 | -2.113924753 | 11.05272687 | -14.63986297 | 9.04E-41 | 8.97E-39 | 81.85813179 |
| DIRAS3 | 1.968079802 | 6.367261196 | 14.61914253 | 1.12E-40 | 1.10E-38 | 81.64544379 |
| ANXA5 | 1.081470996 | 12.11490516 | 14.61910475 | 1.12E-40 | 1.10E-38 | 81.64505615 |
| C10orf75 | -1.249878991 | 8.494301896 | -14.61601824 | 1.16E-40 | 1.13E-38 | 81.61338469 |
| GNG12 | 1.808044652 | 9.472151657 | 14.58449721 | 1.60E-40 | 1.55E-38 | 81.29009538 |
| CLEC18B | 2.011469879 | 5.170657512 | 14.57306229 | 1.80E-40 | 1.73E-38 | 81.17288576 |
| FAM129A | 1.685537142 | 7.590992093 | 14.56266469 | 2.01E-40 | 1.92E-38 | 81.06634136 |
| SSTR2 | -2.108017373 | 7.976506084 | -14.55453049 | 2.19E-40 | 2.08E-38 | 80.98301178 |
| DNM3 | -1.191334325 | 10.58137081 | -14.55171756 | 2.25E-40 | 2.13E-38 | 80.95419956 |
| CACNG2 | -3.099111724 | 5.663798579 | -14.55042589 | 2.28E-40 | 2.15E-38 | 80.94097004 |
| LRRTM4 | -1.850570978 | 8.125916139 | -14.54617301 | 2.38E-40 | 2.23E-38 | 80.89741467 |
| HMP19 | -2.450653464 | 11.31393164 | -14.54542557 | 2.40E-40 | 2.24E-38 | 80.88976042 |
| NCRNA00093 | -2.501004387 | 4.651959177 | -14.52941622 | 2.83E-40 | 2.61E-38 | 80.72585286 |
| RANBP17 | -2.138614145 | 5.786012024 | -14.52502393 | 2.96E-40 | 2.72E-38 | 80.68089656 |
| KCNK3 | -2.409002297 | 7.801686567 | -14.49367439 | 4.10E-40 | 3.74E-38 | 80.36018781 |
| MMP14 | 1.617848011 | 9.849242787 | 14.48762624 | 4.36E-40 | 3.94E-38 | 80.29834752 |
| WEE1 | 1.511947179 | 7.891846354 | 14.48536319 | 4.46E-40 | 4.02E-38 | 80.27521146 |
| SLC2A10 | 1.679312938 | 7.202883699 | 14.46972945 | 5.24E-40 | 4.66E-38 | 80.11542174 |
| FBXO39 | 1.590898848 | 1.200472801 | 14.46170929 | 5.70E-40 | 5.00E-38 | 80.03347678 |
| TCEAL2 | -1.124141072 | 10.89852382 | -14.45406813 | 6.16E-40 | 5.38E-38 | 79.95542181 |
| KSR2 | -2.49533495 | 7.921304705 | -14.44356984 | 6.87E-40 | 5.97E-38 | 79.84820899 |
| PCDH11X | -2.39437433 | 7.315846416 | -14.39152908 | 1.17E-39 | 1.01E-37 | 79.3172276 |
| LOC145837 | -1.749538147 | 4.729542431 | -14.38440566 | 1.26E-39 | 1.08E-37 | 79.24460842 |
| FAM123C | -2.912376533 | 6.991508557 | -14.37358063 | 1.41E-39 | 1.20E-37 | 79.1342823 |
| C9orf44 | 2.395667171 | 1.179523058 | 14.36890834 | 1.48E-39 | 1.25E-37 | 79.08667424 |
| INA | -2.765229465 | 9.837270827 | -14.35989076 | 1.62E-39 | 1.36E-37 | 78.99480845 |
| LOC100302640 | -1.020415449 | 4.825338764 | -14.35730429 | 1.67E-39 | 1.39E-37 | 78.96846349 |
| PARP9 | 1.122071997 | 9.404086183 | 14.35470089 | 1.71E-39 | 1.42E-37 | 78.94194817 |
| C1RL | 1.512886005 | 7.353319732 | 14.34152744 | 1.96E-39 | 1.62E-37 | 78.80780893 |
| CUX2 | -2.896480192 | 7.621512607 | -14.33614278 | 2.07E-39 | 1.70E-37 | 78.75299434 |
| PDE2A | -1.776102325 | 10.1433094 | -14.33194329 | 2.16E-39 | 1.76E-37 | 78.71025054 |
| PSRC1 | 1.387224461 | 9.166505139 | 14.32631736 | 2.29E-39 | 1.86E-37 | 78.65299623 |
| APOBEC3F | 1.295227071 | 4.818868667 | 14.32311753 | 2.37E-39 | 1.91E-37 | 78.62043624 |
| ARAP3 | 1.289056386 | 7.810491079 | 14.32288226 | 2.37E-39 | 1.91E-37 | 78.6180424 |
| SLC43A3 | 1.644855992 | 7.657841918 | 14.31143737 | 2.67E-39 | 2.14E-37 | 78.50161054 |
| RTN1 | -1.433193905 | 12.57822066 | -14.30482806 | 2.86E-39 | 2.27E-37 | 78.43439034 |
| KIAA0040 | 1.697941436 | 7.5801105 | 14.29689998 | 3.10E-39 | 2.46E-37 | 78.35377486 |
| FLNC | 2.28131114 | 9.12479385 | 14.27896892 | 3.73E-39 | 2.94E-37 | 78.17151557 |
| BCL7A | -1.084694416 | 10.13406999 | -14.26973483 | 4.10E-39 | 3.22E-37 | 78.07769414 |
| 3-Sep | -1.135549979 | 12.59238071 | -14.26165548 | 4.45E-39 | 3.49E-37 | 77.99562655 |
| PION | 1.374837658 | 6.956056533 | 14.26009115 | 4.52E-39 | 3.51E-37 | 77.97973882 |
| SLC9A2 | -1.919584918 | 4.461669906 | -14.26001855 | 4.52E-39 | 3.51E-37 | 77.97900148 |
| RGR | -2.480525678 | 6.681560173 | -14.25475796 | 4.78E-39 | 3.67E-37 | 77.92557921 |
| CYS1 | -1.490179023 | 6.769112765 | -14.25474641 | 4.78E-39 | 3.67E-37 | 77.92546191 |
| TPTE2P1 | -1.820053429 | 6.208548532 | -14.23517814 | 5.84E-39 | 4.45E-37 | 77.72681656 |
| ANTXR2 | 1.148398661 | 7.899312417 | 14.23356825 | 5.93E-39 | 4.51E-37 | 77.71047916 |
| MYBPH | 2.009565717 | 2.116757875 | 14.23217004 | 6.02E-39 | 4.56E-37 | 77.6962905 |
| TRPM8 | 2.816465084 | 2.384289929 | 14.22477424 | 6.49E-39 | 4.90E-37 | 77.62124994 |
| TNFAIP6 | 2.15441391 | 4.060850231 | 14.21322414 | 7.31E-39 | 5.49E-37 | 77.50409182 |
| UNC5A | -1.861217593 | 8.931524666 | -14.20494045 | 7.95E-39 | 5.94E-37 | 77.42009163 |
| ARPP21 | -1.642036388 | 9.389330654 | -14.18866897 | 9.39E-39 | 6.94E-37 | 77.25515297 |
| C7orf54 | -1.115869639 | 3.189822459 | -14.13858343 | 1.57E-38 | 1.14E-36 | 76.74796483 |
| DGCR9 | -1.63926431 | 6.9186628 | -14.08192144 | 2.79E-38 | 2.01E-36 | 76.17511945 |
| FMOD | 2.469367194 | 6.250228479 | 14.07870903 | 2.88E-38 | 2.06E-36 | 76.14267245 |
| ABCC8 | -2.344099143 | 8.628134726 | -14.06995414 | 3.15E-38 | 2.23E-36 | 76.05426004 |
| RNF135 | 1.20504067 | 7.158097946 | 14.05900938 | 3.53E-38 | 2.49E-36 | 75.94376672 |
| SCG3 | -1.629447355 | 13.22411909 | -14.05054224 | 3.84E-38 | 2.70E-36 | 75.85831218 |
| C2orf85 | -2.454919746 | 8.151104012 | -14.03653533 | 4.43E-38 | 3.11E-36 | 75.71699717 |
| STAR | -1.674319061 | 4.943887085 | -14.03303244 | 4.59E-38 | 3.21E-36 | 75.68166634 |
| SMOC1 | -2.258056997 | 13.14426282 | -14.0215028 | 5.16E-38 | 3.58E-36 | 75.5654036 |
| YPEL4 | -1.546707652 | 8.128795157 | -14.02098014 | 5.19E-38 | 3.59E-36 | 75.56013426 |
| LPPR3 | -2.556763121 | 6.660851102 | -14.0159843 | 5.46E-38 | 3.76E-36 | 75.50977097 |
| CSDC2 | -2.198613901 | 10.12622325 | -14.00860308 | 5.89E-38 | 4.04E-36 | 75.43537523 |
| DNAH11 | 2.015767566 | 2.416658069 | 14.00565026 | 6.07E-38 | 4.15E-36 | 75.4056184 |
| ALPK3 | 1.132798891 | 7.489290024 | 13.99885137 | 6.50E-38 | 4.42E-36 | 75.33711358 |
| HOXC13 | 1.826255842 | 0.573098415 | 13.98946141 | 7.15E-38 | 4.83E-36 | 75.24252567 |
| FLRT1 | -1.283964918 | 9.049554299 | -13.97977737 | 7.89E-38 | 5.31E-36 | 75.14500477 |
| MIR155HG | 1.423238587 | 2.32431022 | 13.97918624 | 7.94E-38 | 5.31E-36 | 75.13905285 |
| FAM114A1 | 1.340462581 | 6.306439763 | 13.97522552 | 8.27E-38 | 5.49E-36 | 75.09917678 |
| GFRA1 | -2.21568591 | 9.477597282 | -13.95934229 | 9.71E-38 | 6.35E-36 | 74.93931623 |
| CASP4 | 1.397268188 | 6.334807314 | 13.9541416 | 1.02E-37 | 6.65E-36 | 74.88699023 |
| SORCS3 | -1.524019441 | 8.701809849 | -13.94385161 | 1.14E-37 | 7.36E-36 | 74.78348441 |
| DUSP9 | -2.111187837 | 5.856417944 | -13.87088742 | 2.38E-37 | 1.53E-35 | 74.05052424 |
| ENPP1 | 1.452202597 | 4.975243959 | 13.85538495 | 2.79E-37 | 1.76E-35 | 73.89501652 |
| HOXB4 | 2.246728193 | 1.755446187 | 13.8494797 | 2.96E-37 | 1.86E-35 | 73.83580058 |
| SERPINA5 | 2.843569421 | 3.392034401 | 13.84501383 | 3.09E-37 | 1.94E-35 | 73.7910259 |
| LOC254559 | -1.720767467 | 10.72611374 | -13.82953795 | 3.62E-37 | 2.26E-35 | 73.6359154 |
| SOD2 | 1.423524582 | 11.74604211 | 13.80766646 | 4.51E-37 | 2.80E-35 | 73.41683712 |
| GPR1 | 1.990156213 | 1.255963525 | 13.80539276 | 4.62E-37 | 2.86E-35 | 73.39407138 |
| CCDC18 | 1.446146118 | 4.644336494 | 13.80182335 | 4.79E-37 | 2.95E-35 | 73.35833554 |
| PTPRT | -2.217401881 | 9.619826419 | -13.77092625 | 6.54E-37 | 3.97E-35 | 73.04917845 |
| PSD | -1.905541575 | 10.51777956 | -13.76944709 | 6.64E-37 | 4.01E-35 | 73.03438586 |
| LEPREL1 | 1.57280493 | 6.678568262 | 13.75620257 | 7.59E-37 | 4.56E-35 | 72.90196382 |
| CARD16 | 1.827861263 | 4.78349467 | 13.75547123 | 7.64E-37 | 4.58E-35 | 72.89465343 |
| C1R | 1.801491492 | 9.895176834 | 13.75091546 | 8.00E-37 | 4.78E-35 | 72.84911827 |
| PTPN22 | 1.650615866 | 2.243892004 | 13.74285749 | 8.68E-37 | 5.15E-35 | 72.76859518 |
| ANKRD53 | 1.44179772 | 3.346896022 | 13.74020471 | 8.92E-37 | 5.28E-35 | 72.74209072 |
| FAIM2 | -1.194135716 | 12.83560971 | -13.7271765 | 1.02E-36 | 5.96E-35 | 72.61195734 |
| KDELC2 | 1.186000271 | 8.243579363 | 13.71505997 | 1.15E-36 | 6.70E-35 | 72.49098098 |
| RBMS1 | 1.002824582 | 8.324601405 | 13.70584866 | 1.26E-36 | 7.33E-35 | 72.39904425 |
| SH3GL2 | -1.947074616 | 10.43173337 | -13.69614841 | 1.39E-36 | 8.06E-35 | 72.30225817 |
| HAR1A | -2.111436346 | 5.118583206 | -13.69465924 | 1.41E-36 | 8.16E-35 | 72.28740249 |
| NNMT | 2.320276774 | 5.325545269 | 13.67851556 | 1.66E-36 | 9.52E-35 | 72.12640366 |
| COL4A2 | 1.868152892 | 10.04859443 | 13.6642456 | 1.92E-36 | 1.09E-34 | 71.98416406 |
| EMILIN1 | 1.194938809 | 8.852425618 | 13.65539973 | 2.09E-36 | 1.19E-34 | 71.89602482 |
| NEK6 | 1.020928975 | 9.589294245 | 13.6504327 | 2.20E-36 | 1.25E-34 | 71.84654545 |
| MCF2L2 | -1.65901346 | 8.196539265 | -13.64388095 | 2.35E-36 | 1.33E-34 | 71.78129254 |
| CCDC46 | 1.194451349 | 6.199576109 | 13.63831406 | 2.49E-36 | 1.40E-34 | 71.7258598 |
| EMILIN3 | 3.130883695 | 6.044715737 | 13.63506191 | 2.57E-36 | 1.44E-34 | 71.69348116 |
| IQGAP2 | 2.116804627 | 7.088557718 | 13.62797993 | 2.76E-36 | 1.54E-34 | 71.62298467 |
| PLEKHA4 | 1.933885127 | 8.595060597 | 13.62389283 | 2.87E-36 | 1.60E-34 | 71.58230804 |
| COL8A1 | 2.576025679 | 3.79612917 | 13.61531813 | 3.13E-36 | 1.73E-34 | 71.49698714 |
| TET1 | -1.339656413 | 6.81928774 | -13.60637092 | 3.43E-36 | 1.88E-34 | 71.40798626 |
| C13orf18 | 1.507648841 | 6.599141277 | 13.59800045 | 3.73E-36 | 2.04E-34 | 71.32474701 |
| ALDOC | -1.444307013 | 14.14697322 | -13.5962087 | 3.79E-36 | 2.07E-34 | 71.30693224 |
| PDCD1LG2 | 1.875279574 | 4.486985383 | 13.57670357 | 4.61E-36 | 2.49E-34 | 71.11306979 |
| S100A11 | 1.527322808 | 8.943383286 | 13.55186644 | 5.92E-36 | 3.16E-34 | 70.86640038 |
| RAD54B | 1.187792259 | 5.504008728 | 13.53718346 | 6.86E-36 | 3.64E-34 | 70.72067591 |
| APOBEC3G | 1.360233723 | 6.024225586 | 13.52940138 | 7.41E-36 | 3.91E-34 | 70.64347097 |
| BEX1 | -1.095478173 | 11.53940726 | -13.52905428 | 7.44E-36 | 3.91E-34 | 70.64002789 |
| SEMA6B | -1.276993548 | 10.29399588 | -13.51818991 | 8.29E-36 | 4.35E-34 | 70.53228013 |
| MEOX2 | 3.102230396 | 3.119956323 | 13.51633003 | 8.45E-36 | 4.42E-34 | 70.51383882 |
| C9orf125 | -1.50330787 | 8.332094797 | -13.50984616 | 9.01E-36 | 4.71E-34 | 70.44955826 |
| WDR86 | -1.655349549 | 6.129563293 | -13.49953074 | 9.99E-36 | 5.19E-34 | 70.34732202 |
| LSP1 | 1.902710299 | 6.610529118 | 13.48851671 | 1.12E-35 | 5.77E-34 | 70.23820239 |
| DSCAML1 | -1.657116874 | 9.966394034 | -13.48825121 | 1.12E-35 | 5.77E-34 | 70.23557251 |
| SSTR1 | -2.606640885 | 7.353803904 | -13.47168316 | 1.32E-35 | 6.77E-34 | 70.07150803 |
| HMX1 | -2.435216286 | 6.633100634 | -13.46828188 | 1.37E-35 | 6.99E-34 | 70.0378388 |
| GRID1 | -1.06948075 | 9.855374294 | -13.46045167 | 1.48E-35 | 7.54E-34 | 69.9603428 |
| GBP1 | 1.862505298 | 8.438445871 | 13.46002454 | 1.48E-35 | 7.55E-34 | 69.95611606 |
| SRPX2 | 2.399334694 | 4.901797394 | 13.4524693 | 1.60E-35 | 8.13E-34 | 69.88136279 |
| STIL | 1.146899513 | 5.950726754 | 13.43170283 | 1.97E-35 | 9.95E-34 | 69.67599709 |
| SAMD9 | 1.292221194 | 7.679690301 | 13.42362207 | 2.13E-35 | 1.07E-33 | 69.59612468 |
| CDR1 | -1.983522486 | 6.212122219 | -13.41939342 | 2.22E-35 | 1.12E-33 | 69.55433672 |
| LRGUK | 1.63878511 | 2.482508203 | 13.38859883 | 3.02E-35 | 1.50E-33 | 69.25021018 |
| NFE2L3 | 1.018793845 | 6.201457338 | 13.37240765 | 3.55E-35 | 1.75E-33 | 69.09044024 |
| GNG5 | 1.154830434 | 9.464588608 | 13.37119745 | 3.60E-35 | 1.77E-33 | 69.07850202 |
| KIAA0495 | 1.41384416 | 7.746237808 | 13.36832939 | 3.70E-35 | 1.81E-33 | 69.05021171 |
| CMYA5 | 2.069039086 | 6.153555182 | 13.3610043 | 3.98E-35 | 1.94E-33 | 68.97797067 |
| SP140L | 1.260439733 | 6.217660218 | 13.3602065 | 4.01E-35 | 1.95E-33 | 68.97010383 |
| SYT15 | -1.184591935 | 6.626004422 | -13.34173315 | 4.82E-35 | 2.32E-33 | 68.78800655 |
| B3GNT5 | 1.443299249 | 7.278666383 | 13.3348095 | 5.16E-35 | 2.48E-33 | 68.71978928 |
| F11R | 1.074831668 | 8.02250412 | 13.30039792 | 7.27E-35 | 3.46E-33 | 68.38099157 |
| FRRS1 | 1.562738492 | 2.22540264 | 13.2913396 | 7.95E-35 | 3.78E-33 | 68.29187841 |
| ATOH8 | -1.664429152 | 10.25162968 | -13.28400983 | 8.55E-35 | 4.06E-33 | 68.21979177 |
| CAPN5 | 1.065534724 | 10.05754372 | 13.27322479 | 9.52E-35 | 4.50E-33 | 68.11375811 |
| REPS2 | -1.56231678 | 9.69273513 | -13.26609771 | 1.02E-34 | 4.80E-33 | 68.04371071 |
| ELFN2 | -1.90094364 | 10.52840631 | -13.26601179 | 1.02E-34 | 4.80E-33 | 68.04286642 |
| RAB33A | -1.274715187 | 8.274192827 | -13.26528215 | 1.03E-34 | 4.83E-33 | 68.0356963 |
| KIAA1409 | -1.780437135 | 9.110555723 | -13.26383029 | 1.05E-34 | 4.89E-33 | 68.02142958 |
| CHGB | -2.102849111 | 10.64364054 | -13.26095001 | 1.08E-34 | 5.02E-33 | 67.9931287 |
| COL4A1 | 2.0853649 | 9.532178143 | 13.23058686 | 1.45E-34 | 6.76E-33 | 67.69496992 |
| LOC541471 | 1.608412984 | 5.027035629 | 13.22921491 | 1.47E-34 | 6.84E-33 | 67.68150557 |
| PABPC5 | -1.665037063 | 6.814592985 | -13.21700263 | 1.66E-34 | 7.69E-33 | 67.56168363 |
| PARP12 | 1.1231717 | 7.769450426 | 13.21040874 | 1.77E-34 | 8.17E-33 | 67.49700945 |
| CHRM4 | -1.761670425 | 4.960583543 | -13.20010739 | 1.96E-34 | 8.98E-33 | 67.39600334 |
| XKR8 | 1.371699209 | 6.325880968 | 13.1884278 | 2.21E-34 | 1.00E-32 | 67.2815298 |
| CABP4 | 1.248919251 | 2.683028655 | 13.16003988 | 2.92E-34 | 1.32E-32 | 67.00350205 |
| HOXC8 | 1.837021832 | 0.811902819 | 13.15931762 | 2.94E-34 | 1.32E-32 | 66.99643216 |
| CRISPLD1 | 1.253773486 | 9.77763798 | 13.15399777 | 3.10E-34 | 1.39E-32 | 66.94436423 |
| PLP2 | 1.526633588 | 7.42205946 | 13.14628682 | 3.34E-34 | 1.49E-32 | 66.86891182 |
| ACTL6B | -2.400803869 | 8.103668318 | -13.12329228 | 4.20E-34 | 1.86E-32 | 66.64403715 |
| FBLL1 | -1.56015696 | 8.069654893 | -13.12063646 | 4.31E-34 | 1.91E-32 | 66.61807708 |
| UPP1 | 1.241752421 | 7.369663508 | 13.10960632 | 4.80E-34 | 2.13E-32 | 66.51028761 |
| HSPA7 | 2.093307805 | 4.376813823 | 13.10151385 | 5.20E-34 | 2.29E-32 | 66.43123426 |
| DOK6 | -1.783686897 | 8.350402253 | -13.09606797 | 5.49E-34 | 2.40E-32 | 66.37804831 |
| CAPG | 1.581783903 | 9.226533777 | 13.08915511 | 5.88E-34 | 2.56E-32 | 66.31055124 |
| TEAD2 | 1.22348333 | 6.899137679 | 13.08674376 | 6.02E-34 | 2.62E-32 | 66.28701095 |
| FUT9 | -1.395657405 | 10.23039939 | -13.08258598 | 6.27E-34 | 2.72E-32 | 66.24642667 |
| NAMPT | 1.12655154 | 10.25002884 | 13.07776895 | 6.57E-34 | 2.84E-32 | 66.19941533 |
| SCD | -1.178989678 | 14.58912621 | -13.07078161 | 7.04E-34 | 3.04E-32 | 66.13123825 |
| CLEC18A | 1.485654495 | 5.37781106 | 13.06682 | 7.32E-34 | 3.15E-32 | 66.09259201 |
| VIM | 1.83473109 | 13.64357017 | 13.06283241 | 7.62E-34 | 3.26E-32 | 66.05369813 |
| CASKIN1 | -1.366106632 | 9.546186897 | -13.06251874 | 7.64E-34 | 3.26E-32 | 66.0506389 |
| RAB42 | 1.429199948 | 3.176069971 | 13.04849586 | 8.77E-34 | 3.73E-32 | 65.91391196 |
| LTF | 3.781536853 | 4.662319886 | 13.04700322 | 8.90E-34 | 3.77E-32 | 65.89936257 |
| GJC1 | 1.270330791 | 7.258989357 | 13.04154899 | 9.39E-34 | 3.96E-32 | 65.8462051 |
| APOBEC3C | 1.36068085 | 6.038029105 | 13.03516091 | 1.00E-33 | 4.21E-32 | 65.78396023 |
| FAM110B | -1.209160604 | 10.71520097 | -13.02426106 | 1.11E-33 | 4.66E-32 | 65.67778812 |
| CIITA | 1.993031142 | 6.374072048 | 13.012319 | 1.25E-33 | 5.21E-32 | 65.56151493 |
| HFE | 1.22365789 | 4.795506046 | 12.99624935 | 1.47E-33 | 6.08E-32 | 65.40513774 |
| RINL | 1.034175347 | 6.063421674 | 12.98394754 | 1.65E-33 | 6.83E-32 | 65.28549144 |
| FCGR2C | 1.88788432 | 3.333685432 | 12.96786823 | 1.94E-33 | 7.95E-32 | 65.12919107 |
| HRH3 | -2.508918873 | 6.648921866 | -12.9635803 | 2.02E-33 | 8.28E-32 | 65.08752631 |
| GBP5 | 2.051436703 | 4.144205479 | 12.94266829 | 2.48E-33 | 1.01E-31 | 64.88442834 |
| FLNA | 1.14268136 | 12.48243749 | 12.93784505 | 2.60E-33 | 1.06E-31 | 64.83760836 |
| MIPOL1 | -1.508614252 | 5.507276639 | -12.93305474 | 2.72E-33 | 1.10E-31 | 64.79111663 |
| ADPRH | 1.000431058 | 6.692594213 | 12.93150513 | 2.77E-33 | 1.12E-31 | 64.77607898 |
| HSF2BP | -1.349320731 | 6.019006304 | -12.93132295 | 2.77E-33 | 1.12E-31 | 64.77431111 |
| ISL2 | 1.959555524 | 1.745576886 | 12.92234083 | 3.03E-33 | 1.22E-31 | 64.6871657 |
| CDNF | -1.09860991 | 4.773428114 | -12.9181912 | 3.15E-33 | 1.27E-31 | 64.64691583 |
| SMPD3 | -1.327889772 | 8.655103546 | -12.90747818 | 3.50E-33 | 1.40E-31 | 64.54303382 |
| SWAP70 | 1.017552834 | 8.975565819 | 12.90318773 | 3.65E-33 | 1.46E-31 | 64.50144233 |
| GABRA3 | -2.11036738 | 7.907874211 | -12.89218048 | 4.06E-33 | 1.61E-31 | 64.39477039 |
| MTMR7 | -1.357902723 | 8.209395601 | -12.88279204 | 4.46E-33 | 1.76E-31 | 64.30382294 |
| CGREF1 | -1.247232041 | 8.879819767 | -12.87306927 | 4.90E-33 | 1.93E-31 | 64.20967203 |
| AQP5 | 2.668628991 | 2.315059451 | 12.86073917 | 5.53E-33 | 2.17E-31 | 64.0903248 |
| ADHFE1 | -1.097805711 | 8.844500982 | -12.85234147 | 6.00E-33 | 2.35E-31 | 64.00907384 |
| DMRTA2 | 2.65601162 | 3.381388638 | 12.84481015 | 6.46E-33 | 2.52E-31 | 63.93622837 |
| LRRC16B | -1.14663106 | 8.238842023 | -12.84147919 | 6.67E-33 | 2.60E-31 | 63.90401706 |
| HPD | 2.377410468 | 2.157216868 | 12.82631471 | 7.74E-33 | 3.00E-31 | 63.75742642 |
| C3orf59 | -1.047206923 | 7.908284393 | -12.81627244 | 8.53E-33 | 3.30E-31 | 63.6603992 |
| GRIP1 | -1.757244975 | 5.411626436 | -12.81020636 | 9.05E-33 | 3.49E-31 | 63.60180833 |
| KCNB1 | -1.598364444 | 7.910816451 | -12.78581441 | 1.15E-32 | 4.41E-31 | 63.36635498 |
| CENPE | 1.831395804 | 5.345538888 | 12.78270813 | 1.18E-32 | 4.53E-31 | 63.33638682 |
| GSDMD | 1.147488091 | 7.887227111 | 12.78134613 | 1.20E-32 | 4.58E-31 | 63.32324787 |
| RPS2P32 | 1.323775074 | 2.960812517 | 12.77771607 | 1.24E-32 | 4.73E-31 | 63.28823314 |
| LATS2 | 1.145489545 | 6.812679328 | 12.77256437 | 1.31E-32 | 4.97E-31 | 63.23854976 |
| RNF165 | -1.45446155 | 8.2526761 | -12.76281092 | 1.44E-32 | 5.44E-31 | 63.14451485 |
| PLCE1 | 1.19705381 | 8.514687196 | 12.74178684 | 1.76E-32 | 6.64E-31 | 62.94194307 |
| SERPINA1 | 1.785928053 | 7.904203377 | 12.73956087 | 1.80E-32 | 6.77E-31 | 62.9205054 |
| ACTN1 | 1.40146302 | 10.2897164 | 12.72302817 | 2.11E-32 | 7.91E-31 | 62.76134414 |
| CLCF1 | 1.774925173 | 4.030595027 | 12.72189979 | 2.14E-32 | 7.98E-31 | 62.75048499 |
| CD302 | 1.05228922 | 8.944410012 | 12.7160778 | 2.26E-32 | 8.43E-31 | 62.69446427 |
| SLC25A27 | -1.082139069 | 8.760127711 | -12.71239715 | 2.34E-32 | 8.72E-31 | 62.65905498 |
| KLRC3 | -2.088845715 | 7.391539011 | -12.70931304 | 2.42E-32 | 8.95E-31 | 62.62938868 |
| TMEM151B | -1.858050101 | 9.801803149 | -12.70823559 | 2.44E-32 | 9.03E-31 | 62.61902545 |
| GGT8P | 1.595973815 | 1.157570637 | 12.70132565 | 2.61E-32 | 9.62E-31 | 62.55257452 |
| BCL2L12 | 1.260040469 | 5.403615982 | 12.69135193 | 2.88E-32 | 1.06E-30 | 62.45669313 |
| TNC | 1.694442344 | 11.62213904 | 12.68603094 | 3.03E-32 | 1.11E-30 | 62.40555619 |
| LOC284276 | 1.147658653 | 0.659253424 | 12.67671997 | 3.31E-32 | 1.21E-30 | 62.31610065 |
| VSTM2A | -2.729560999 | 7.63383229 | -12.66651394 | 3.66E-32 | 1.33E-30 | 62.21808494 |
| IGF2BP2 | 2.205521716 | 4.013639189 | 12.6485605 | 4.36E-32 | 1.56E-30 | 62.04576472 |
| FAM19A2 | -1.995496849 | 7.242099801 | -12.64810598 | 4.37E-32 | 1.57E-30 | 62.04140385 |
| GLRA3 | -2.624271031 | 4.579396037 | -12.64628523 | 4.45E-32 | 1.59E-30 | 62.02393537 |
| IFI44 | 1.173776057 | 8.008329832 | 12.64438777 | 4.53E-32 | 1.62E-30 | 62.00573239 |
| FAM13C | -1.086658859 | 8.985905006 | -12.63514199 | 4.96E-32 | 1.76E-30 | 61.91705456 |
| SIT1 | 1.35519126 | 1.392225205 | 12.61182872 | 6.22E-32 | 2.20E-30 | 61.69360326 |
| NRSN1 | -1.841562359 | 9.226869389 | -12.60506898 | 6.63E-32 | 2.35E-30 | 61.62885333 |
| HOXD10 | 2.446283127 | 1.535723131 | 12.5976845 | 7.13E-32 | 2.52E-30 | 61.55813985 |
| C21orf7 | 1.769031616 | 5.3633512 | 12.59107356 | 7.60E-32 | 2.68E-30 | 61.49485211 |
| VGLL2 | 1.578851112 | 0.791791495 | 12.58733255 | 7.88E-32 | 2.77E-30 | 61.45904644 |
| ENHO | -1.338570186 | 11.40008212 | -12.57690066 | 8.71E-32 | 3.06E-30 | 61.359231 |
| CKMT1A | -2.109357228 | 6.58362794 | -12.56776264 | 9.51E-32 | 3.33E-30 | 61.27183133 |
| EPHA10 | -1.81246598 | 6.689718274 | -12.54664198 | 1.17E-31 | 4.07E-30 | 61.06995253 |
| PID1 | -1.210440649 | 10.70231311 | -12.53446369 | 1.31E-31 | 4.55E-30 | 60.95362917 |
| DEPDC1 | 1.941418783 | 4.001442233 | 12.52449473 | 1.44E-31 | 4.99E-30 | 60.85845296 |
| LOC285696 | -1.718841902 | 3.507444508 | -12.52242623 | 1.47E-31 | 5.08E-30 | 60.8387095 |
| CDK2 | 1.091067338 | 7.858395837 | 12.51583702 | 1.57E-31 | 5.41E-30 | 60.77582804 |
| LOC157627 | -2.111654405 | 7.139771219 | -12.49365236 | 1.94E-31 | 6.66E-30 | 60.56424607 |
| ATCAY | -1.8796354 | 11.69850226 | -12.48526277 | 2.11E-31 | 7.21E-30 | 60.48428373 |
| PLAU | 1.878193023 | 6.489970554 | 12.48131232 | 2.19E-31 | 7.47E-30 | 60.44664128 |
| SHCBP1 | 1.460891267 | 5.70482793 | 12.47986368 | 2.22E-31 | 7.56E-30 | 60.43283929 |
| NUAK2 | 1.2549741 | 5.53062013 | 12.47143535 | 2.41E-31 | 8.14E-30 | 60.35255471 |
| NAPSB | 2.639146358 | 6.791418521 | 12.46499154 | 2.56E-31 | 8.63E-30 | 60.29119322 |
| CXCL10 | 2.564140014 | 3.760332659 | 12.4629627 | 2.61E-31 | 8.79E-30 | 60.27187689 |
| MSR1 | 1.75061909 | 7.682978299 | 12.45600825 | 2.79E-31 | 9.38E-30 | 60.20567742 |
| STXBP6 | -1.403775951 | 6.507674017 | -12.45080616 | 2.94E-31 | 9.85E-30 | 60.1561715 |
| MN1 | -1.261433503 | 9.442237117 | -12.44911498 | 2.98E-31 | 9.99E-30 | 60.1400797 |
| OSR2 | 2.214657548 | 1.909467454 | 12.44535895 | 3.09E-31 | 1.03E-29 | 60.10434461 |
| PDZD4 | -1.107906716 | 12.16832203 | -12.4451085 | 3.10E-31 | 1.03E-29 | 60.10196205 |
| TXLNB | 1.854831758 | 5.186502058 | 12.44196425 | 3.20E-31 | 1.06E-29 | 60.07205221 |
| IL13RA2 | 2.927400419 | 3.877903702 | 12.43901504 | 3.29E-31 | 1.09E-29 | 60.0440014 |
| HECW1 | -1.997781619 | 7.266614896 | -12.43878781 | 3.29E-31 | 1.09E-29 | 60.04184028 |
| FABP5 | 2.094937895 | 4.570252981 | 12.4384163 | 3.31E-31 | 1.09E-29 | 60.03830704 |
| GLUD2 | -1.051801469 | 10.165851 | -12.43202061 | 3.52E-31 | 1.16E-29 | 59.97748947 |
| VSTM1 | 1.163542796 | 0.653784552 | 12.42976932 | 3.59E-31 | 1.18E-29 | 59.95608551 |
| ELAVL2 | -1.842806269 | 7.847627325 | -12.42827371 | 3.64E-31 | 1.20E-29 | 59.94186733 |
| KIAA1644 | -2.301410502 | 8.387298261 | -12.42502767 | 3.76E-31 | 1.23E-29 | 59.91101154 |
| OSMR | 1.51367228 | 7.388384356 | 12.39724985 | 4.91E-31 | 1.59E-29 | 59.64714069 |
| HLA-DRA | 1.889075198 | 11.57484595 | 12.37819289 | 5.89E-31 | 1.90E-29 | 59.4662948 |
| ST8SIA3 | -2.2247903 | 9.330325843 | -12.37780669 | 5.91E-31 | 1.91E-29 | 59.46263137 |
| RASEF | 1.657697261 | 1.386485256 | 12.377013 | 5.96E-31 | 1.92E-29 | 59.45510285 |
| HAR1B | -1.64176184 | 2.905714404 | -12.37493978 | 6.08E-31 | 1.95E-29 | 59.43543846 |
| FGF13 | -1.82298825 | 8.093822566 | -12.37003612 | 6.37E-31 | 2.04E-29 | 59.38893454 |
| PPIC | 1.177273205 | 6.546526458 | 12.35927621 | 7.06E-31 | 2.26E-29 | 59.28692751 |
| LCTL | 1.659768186 | 3.357360751 | 12.35816413 | 7.14E-31 | 2.28E-29 | 59.2763874 |
| FERMT1 | -2.170810791 | 9.944015508 | -12.35677637 | 7.23E-31 | 2.31E-29 | 59.26323518 |
| LOC440040 | -2.096657577 | 3.916621148 | -12.35611136 | 7.28E-31 | 2.32E-29 | 59.25693293 |
| HOXA9 | 2.047099807 | 1.065985443 | 12.34770399 | 7.89E-31 | 2.50E-29 | 59.17727273 |
| SLC25A21 | -1.575731832 | 3.09183864 | -12.34563468 | 8.05E-31 | 2.54E-29 | 59.15767038 |
| BEND4 | -1.943665359 | 4.667495706 | -12.33388488 | 9.00E-31 | 2.84E-29 | 59.04639945 |
| KCNIP3 | -1.310861063 | 10.58309312 | -12.32907712 | 9.43E-31 | 2.96E-29 | 59.00088619 |
| CD74 | 1.667007553 | 13.36568794 | 12.3263912 | 9.67E-31 | 3.03E-29 | 58.97546375 |
| KHDRBS2 | -2.109213109 | 4.594521697 | -12.32057057 | 1.02E-30 | 3.20E-29 | 58.92038134 |
| C21orf63 | 1.250003561 | 7.245962091 | 12.31816821 | 1.05E-30 | 3.26E-29 | 58.89765119 |
| GIMAP2 | 1.018213082 | 6.702845834 | 12.31545094 | 1.07E-30 | 3.33E-29 | 58.87194436 |
| GAS2L3 | 1.804003852 | 3.475221188 | 12.31315891 | 1.10E-30 | 3.40E-29 | 58.85026291 |
| NPNT | 2.089530275 | 7.405080886 | 12.31262386 | 1.10E-30 | 3.41E-29 | 58.8452019 |
| BGN | 1.372090675 | 10.39769243 | 12.30387974 | 1.20E-30 | 3.70E-29 | 58.76250874 |
| OCIAD2 | 1.716753985 | 6.422964718 | 12.28913542 | 1.38E-30 | 4.25E-29 | 58.62314353 |
| CDCA8 | 1.650745636 | 5.627979764 | 12.28277128 | 1.47E-30 | 4.51E-29 | 58.56301678 |
| CLEC18C | 1.608660901 | 2.781124869 | 12.28152278 | 1.48E-30 | 4.55E-29 | 58.5512233 |
| NKX2-5 | 2.11682709 | 1.524101622 | 12.28018364 | 1.50E-30 | 4.60E-29 | 58.5385743 |
| ATP8A1 | -1.126360894 | 10.87492757 | -12.27914841 | 1.52E-30 | 4.63E-29 | 58.52879644 |
| DDX60L | 1.041362957 | 7.583826298 | 12.27074078 | 1.65E-30 | 5.00E-29 | 58.44940179 |
| VASN | 1.847870508 | 6.192157558 | 12.26909494 | 1.67E-30 | 5.08E-29 | 58.43386331 |
| DACH2 | -2.455753985 | 6.282637502 | -12.26739848 | 1.70E-30 | 5.14E-29 | 58.41784816 |
| SPTBN2 | -1.268713304 | 10.96259752 | -12.26301272 | 1.77E-30 | 5.36E-29 | 58.37645048 |
| SFRP4 | 1.939861339 | 7.07756378 | 12.26229854 | 1.78E-30 | 5.38E-29 | 58.36971008 |
| LY6K | -1.165655258 | 5.033819225 | -12.23610788 | 2.29E-30 | 6.87E-29 | 58.12266889 |
| TRIP6 | 1.178718849 | 9.448449968 | 12.23495498 | 2.31E-30 | 6.94E-29 | 58.11180088 |
| CLEC5A | 2.501122884 | 3.643796227 | 12.22778757 | 2.48E-30 | 7.41E-29 | 58.04424839 |
| CCNB1 | 1.132535402 | 7.396308365 | 12.22215544 | 2.61E-30 | 7.80E-29 | 57.99118096 |
| TPRG1 | 1.170637896 | 2.30020446 | 12.22055676 | 2.65E-30 | 7.91E-29 | 57.97612017 |
| SERPINA3 | 2.667355219 | 11.02828832 | 12.21517056 | 2.79E-30 | 8.31E-29 | 57.92538609 |
| RAB32 | 1.1338813 | 6.752877086 | 12.20630984 | 3.04E-30 | 9.03E-29 | 57.84195096 |
| AOX1 | 1.762757907 | 3.307199896 | 12.20561142 | 3.06E-30 | 9.08E-29 | 57.83537584 |
| CKMT1B | -1.915160738 | 8.207981247 | -12.20349143 | 3.12E-30 | 9.24E-29 | 57.81541888 |
| C5orf62 | 1.036778629 | 8.170226452 | 12.19524274 | 3.38E-30 | 9.96E-29 | 57.73778637 |
| CHI3L2 | 2.704717919 | 7.875770758 | 12.18312043 | 3.79E-30 | 1.10E-28 | 57.62374916 |
| SMCR5 | -1.459209126 | 2.863044132 | -12.18081275 | 3.87E-30 | 1.12E-28 | 57.6020474 |
| HOXA10 | 3.071573622 | 2.682185941 | 12.17881908 | 3.95E-30 | 1.14E-28 | 57.58330041 |
| PLA2G2A | 2.394212802 | 1.160870153 | 12.16592157 | 4.46E-30 | 1.29E-28 | 57.46206235 |
| DGCR10 | -1.470152526 | 3.891562667 | -12.16286899 | 4.59E-30 | 1.32E-28 | 57.43337802 |
| GSX2 | 1.808605861 | 1.536762708 | 12.15561907 | 4.92E-30 | 1.41E-28 | 57.36526828 |
| DCTD | 1.090808134 | 9.199290226 | 12.15174442 | 5.10E-30 | 1.46E-28 | 57.32887675 |
| SNX32 | -1.13044048 | 7.153726228 | -12.14867391 | 5.25E-30 | 1.50E-28 | 57.30004238 |
| RDH10 | 1.177655257 | 8.110188531 | 12.14841883 | 5.27E-30 | 1.50E-28 | 57.29764718 |
| HMGA2 | 1.872086088 | 1.069223277 | 12.14670584 | 5.35E-30 | 1.52E-28 | 57.28156287 |
| XAF1 | 1.269809261 | 8.143733332 | 12.13768184 | 5.83E-30 | 1.65E-28 | 57.19685163 |
| KLRC2 | -2.931540509 | 8.071806212 | -12.12285483 | 6.71E-30 | 1.89E-28 | 57.05774078 |
| HPSE2 | -2.830470673 | 5.88101264 | -12.122369 | 6.74E-30 | 1.90E-28 | 57.05318416 |
| C2orf27A | -1.545091056 | 6.854670083 | -12.12156622 | 6.79E-30 | 1.91E-28 | 57.04565513 |
| LOC283392 | -1.798070908 | 3.025480817 | -12.11569623 | 7.18E-30 | 2.02E-28 | 56.99061044 |
| APOBEC3D | 1.138361344 | 3.187590143 | 12.11408717 | 7.29E-30 | 2.05E-28 | 56.9755243 |
| SAA1 | 1.964873088 | 0.901055416 | 12.11156245 | 7.47E-30 | 2.09E-28 | 56.95185553 |
| BANK1 | 1.597820829 | 2.816116073 | 12.10994208 | 7.58E-30 | 2.12E-28 | 56.93666626 |
| INSM2 | -2.324182809 | 4.057416748 | -12.10027586 | 8.31E-30 | 2.32E-28 | 56.84607885 |
| SP7 | -1.062051604 | 2.689726406 | -12.08750727 | 9.38E-30 | 2.61E-28 | 56.72647863 |
| IFI30 | 1.700004787 | 8.549284796 | 12.08563336 | 9.55E-30 | 2.65E-28 | 56.70893207 |
| GRIN3A | -2.265678791 | 7.152101217 | -12.08315562 | 9.77E-30 | 2.71E-28 | 56.68573373 |
| S100A4 | 1.899806987 | 5.489259539 | 12.07829426 | 1.02E-29 | 2.83E-28 | 56.64022595 |
| NMNAT2 | -1.565214421 | 10.31008741 | -12.07715452 | 1.03E-29 | 2.86E-28 | 56.62955823 |
| RND1 | -1.012334401 | 8.877816541 | -12.07278208 | 1.08E-29 | 2.97E-28 | 56.58863806 |
| ARHGAP18 | 1.091988506 | 7.410268127 | 12.06808274 | 1.13E-29 | 3.10E-28 | 56.54466773 |
| EMILIN2 | 1.617602401 | 6.130438497 | 12.06577748 | 1.15E-29 | 3.16E-28 | 56.52310154 |
| AIM1 | 1.542684066 | 5.028776368 | 12.05043483 | 1.33E-29 | 3.63E-28 | 56.37962611 |
| OLFML3 | 1.192220389 | 9.071013612 | 12.04828962 | 1.36E-29 | 3.70E-28 | 56.35957342 |
| CXorf1 | -1.240000277 | 6.609123454 | -12.0479657 | 1.36E-29 | 3.71E-28 | 56.35654562 |
| NAP1L2 | -1.366001379 | 9.086077193 | -12.04366673 | 1.42E-29 | 3.86E-28 | 56.31636689 |
| ITGA5 | 1.193688713 | 8.456034113 | 12.04308102 | 1.43E-29 | 3.88E-28 | 56.3108934 |
| CELSR1 | 1.974683917 | 5.276586664 | 12.04166394 | 1.45E-29 | 3.92E-28 | 56.29765131 |
| CNTN3 | -2.320566162 | 7.183949222 | -12.03980545 | 1.47E-29 | 3.99E-28 | 56.28028572 |
| NCRNA00152 | 1.544086734 | 4.42567329 | 12.03339981 | 1.56E-29 | 4.21E-28 | 56.22044316 |
| CDH18 | -2.249696537 | 6.45766803 | -12.0227507 | 1.73E-29 | 4.64E-28 | 56.12099662 |
| GPR82 | 2.029638573 | 2.759885376 | 12.01354114 | 1.89E-29 | 5.03E-28 | 56.03503272 |
| FCGR3A | 1.780701306 | 9.565580061 | 12.00652766 | 2.01E-29 | 5.37E-28 | 55.96959209 |
| MYD88 | 1.05749799 | 8.496996534 | 12.00590496 | 2.03E-29 | 5.40E-28 | 55.96378291 |
| PLBD1 | 1.437946103 | 5.215205476 | 12.00471111 | 2.05E-29 | 5.44E-28 | 55.95264594 |
| GPR65 | 1.789901177 | 4.991970966 | 12.00181345 | 2.11E-29 | 5.58E-28 | 55.92561727 |
| WDR78 | 1.490006625 | 5.845601718 | 11.99166576 | 2.32E-29 | 6.10E-28 | 55.83099076 |
| CXCL9 | 2.2038206 | 3.469284459 | 11.99021109 | 2.35E-29 | 6.18E-28 | 55.81742964 |
| ANXA2P1 | 1.153886325 | 2.714860243 | 11.98645866 | 2.43E-29 | 6.37E-28 | 55.78245222 |
| KCNJ11 | -1.849195433 | 8.469223572 | -11.9785013 | 2.62E-29 | 6.82E-28 | 55.70829956 |
| MOXD1 | 2.987600744 | 5.722465167 | 11.97367028 | 2.75E-29 | 7.13E-28 | 55.66329392 |
| PLK5P | -1.679418173 | 4.41009558 | -11.97131759 | 2.81E-29 | 7.27E-28 | 55.64138001 |
| SLC8A3 | -1.54037377 | 8.812470841 | -11.96665534 | 2.93E-29 | 7.57E-28 | 55.59796095 |
| RGS22 | 2.149413587 | 2.771416807 | 11.96498435 | 2.98E-29 | 7.68E-28 | 55.58240154 |
| KIF2C | 1.888673144 | 6.072624527 | 11.96173928 | 3.07E-29 | 7.90E-28 | 55.55218856 |
| FBLN5 | 1.403466291 | 8.020902375 | 11.9564762 | 3.23E-29 | 8.29E-28 | 55.5031967 |
| CDKN2C | 1.402718711 | 8.586397362 | 11.95505447 | 3.27E-29 | 8.38E-28 | 55.48996446 |
| ARL9 | 1.5510594 | 1.510589191 | 11.95152955 | 3.38E-29 | 8.64E-28 | 55.45716146 |
| GDF10 | -2.326966458 | 6.429582231 | -11.94940542 | 3.45E-29 | 8.81E-28 | 55.43739678 |
| SLITRK1 | -1.756498114 | 8.570820222 | -11.94885695 | 3.47E-29 | 8.83E-28 | 55.4322937 |
| HLA-DPA1 | 1.782026703 | 10.57288035 | 11.94837222 | 3.48E-29 | 8.86E-28 | 55.42778377 |
| E2F7 | 1.960638389 | 4.251699663 | 11.94253074 | 3.68E-29 | 9.33E-28 | 55.37344261 |
| SLC22A6 | -2.130586458 | 5.723908116 | -11.93476638 | 3.96E-29 | 9.99E-28 | 55.30123671 |
| AKR1C3 | -1.460164051 | 9.062998147 | -11.9340771 | 3.98E-29 | 1.00E-27 | 55.29482797 |
| ADARB2 | -1.763911973 | 7.216910791 | -11.92490367 | 4.34E-29 | 1.09E-27 | 55.20955492 |
| RGMB | -1.065931576 | 11.07539839 | -11.91690083 | 4.68E-29 | 1.17E-27 | 55.13519336 |
| TYMP | 1.835215393 | 6.958520611 | 11.90712396 | 5.13E-29 | 1.28E-27 | 55.04438581 |
| CELF3 | -1.717487829 | 9.395847408 | -11.90272075 | 5.35E-29 | 1.33E-27 | 55.00350249 |
| HS3ST3B1 | 1.682664406 | 1.808638457 | 11.90122758 | 5.42E-29 | 1.35E-27 | 54.98964057 |
| SLC1A1 | -1.281703153 | 9.46545269 | -11.90009758 | 5.48E-29 | 1.36E-27 | 54.97915073 |
| EFEMP2 | 1.420254513 | 8.495955558 | 11.89284071 | 5.87E-29 | 1.45E-27 | 54.91179842 |
| TMEFF2 | -1.725107867 | 8.967069063 | -11.89277837 | 5.87E-29 | 1.45E-27 | 54.91121998 |
| ALDH3A1 | 1.677943242 | 2.333343021 | 11.89002986 | 6.02E-29 | 1.49E-27 | 54.88571664 |
| SOX1 | -1.379161084 | 7.508458112 | -11.88804142 | 6.14E-29 | 1.51E-27 | 54.86726808 |
| USP43 | -1.628110008 | 7.467893892 | -11.8804692 | 6.59E-29 | 1.62E-27 | 54.79702967 |
| FAM46A | 1.105390432 | 7.624371659 | 11.8775635 | 6.77E-29 | 1.66E-27 | 54.77008374 |
| CD97 | 1.168539802 | 8.248602242 | 11.85474028 | 8.38E-29 | 2.05E-27 | 54.55856214 |
| STX1B | -1.328563883 | 10.38270241 | -11.85357703 | 8.47E-29 | 2.07E-27 | 54.54778749 |
| NRG3 | -1.345991931 | 8.424436088 | -11.84200754 | 9.44E-29 | 2.30E-27 | 54.44065739 |
| RUNX1 | 1.528116737 | 7.606888182 | 11.84193752 | 9.45E-29 | 2.30E-27 | 54.44000925 |
| CBFA2T3 | -1.005764255 | 6.653375914 | -11.8391432 | 9.70E-29 | 2.36E-27 | 54.41414363 |
| FN1 | 1.180904692 | 12.46224915 | 11.83668517 | 9.92E-29 | 2.41E-27 | 54.3913937 |
| KCNK4 | -1.603112732 | 5.767110584 | -11.83127113 | 1.04E-28 | 2.53E-27 | 54.3412944 |
| CCDC19 | 1.918455381 | 1.8125597 | 11.82634673 | 1.09E-28 | 2.65E-27 | 54.29573732 |
| ALPK1 | 1.064872213 | 6.716178136 | 11.82293283 | 1.13E-28 | 2.73E-27 | 54.26416058 |
| LOC100130776 | 1.478913593 | 5.176875222 | 11.82210396 | 1.14E-28 | 2.75E-27 | 54.25649475 |
| C9orf64 | 1.283395393 | 6.722860919 | 11.82184576 | 1.14E-28 | 2.75E-27 | 54.25410685 |
| KIF4A | 1.784268984 | 6.193424435 | 11.81959225 | 1.16E-28 | 2.80E-27 | 54.23326707 |
| OAS2 | 1.375965648 | 7.861224766 | 11.81957332 | 1.16E-28 | 2.80E-27 | 54.23309204 |
| DGCR6 | -1.271963497 | 10.36350712 | -11.8193569 | 1.17E-28 | 2.81E-27 | 54.23109071 |
| ULBP3 | 1.40175861 | 2.113404323 | 11.80924974 | 1.28E-28 | 3.07E-27 | 54.13765151 |
| HLA-DOA | 1.848762466 | 7.939360433 | 11.80924974 | 1.28E-28 | 3.07E-27 | 54.13765146 |
| RIMS2 | -2.230986037 | 7.363778785 | -11.80857784 | 1.29E-28 | 3.08E-27 | 54.1314415 |
| RAB3C | -1.918459013 | 5.190662203 | -11.80663293 | 1.31E-28 | 3.13E-27 | 54.11346687 |
| CYP2E1 | -1.451606804 | 6.142997786 | -11.80281352 | 1.36E-28 | 3.24E-27 | 54.07817324 |
| PAX3 | 1.935115342 | 0.786473088 | 11.80155247 | 1.38E-28 | 3.27E-27 | 54.06652175 |
| LOC100128977 | -1.463434304 | 5.122046243 | -11.79880541 | 1.41E-28 | 3.35E-27 | 54.04114291 |
| CECR1 | 1.09326455 | 8.960777741 | 11.77934283 | 1.70E-28 | 4.01E-27 | 53.86143273 |
| PCDH7 | -1.259895193 | 9.817333705 | -11.77028763 | 1.84E-28 | 4.35E-27 | 53.77787805 |
| SFRP2 | -3.115111072 | 9.469981877 | -11.76984305 | 1.85E-28 | 4.36E-27 | 53.77377679 |
| CBLN2 | -2.167525922 | 6.178350073 | -11.76947715 | 1.86E-28 | 4.37E-27 | 53.7704013 |
| CD96 | 1.497817357 | 2.188581192 | 11.76774898 | 1.89E-28 | 4.44E-27 | 53.75445987 |
| LPAR6 | 1.049370569 | 8.257818875 | 11.7617303 | 2.00E-28 | 4.68E-27 | 53.69895106 |
| MAPK8IP2 | -1.106064356 | 10.69241705 | -11.75860529 | 2.06E-28 | 4.81E-27 | 53.67013622 |
| MYL3 | -1.471253743 | 4.681846085 | -11.75459141 | 2.14E-28 | 4.98E-27 | 53.63313184 |
| CDC20 | 1.902936965 | 5.929638425 | 11.75321167 | 2.16E-28 | 5.03E-27 | 53.62041357 |
| ARSD | 1.179128734 | 7.946722291 | 11.75244691 | 2.18E-28 | 5.06E-27 | 53.61336442 |
| FRMPD1 | -1.322244071 | 6.582965785 | -11.75058651 | 2.22E-28 | 5.15E-27 | 53.59621742 |
| DOK3 | 1.051273269 | 6.80955186 | 11.74850442 | 2.26E-28 | 5.24E-27 | 53.57702903 |
| GABRG1 | -2.26402456 | 8.719171502 | -11.74799928 | 2.27E-28 | 5.26E-27 | 53.57237395 |
| C11orf63 | 1.813762414 | 3.771885198 | 11.74453186 | 2.35E-28 | 5.43E-27 | 53.54042358 |
| SPRY1 | 1.26711595 | 7.305985248 | 11.74224955 | 2.40E-28 | 5.53E-27 | 53.51939622 |
| COL5A1 | 1.993501714 | 6.202762529 | 11.74115477 | 2.42E-28 | 5.58E-27 | 53.50931061 |
| CHRNA9 | 1.879817949 | 1.83260671 | 11.73446881 | 2.58E-28 | 5.91E-27 | 53.4477284 |
| ZDHHC12 | 1.001734465 | 7.002911402 | 11.73178681 | 2.64E-28 | 6.06E-27 | 53.42303103 |
| PTX3 | 1.643742177 | 4.973945756 | 11.73062608 | 2.67E-28 | 6.11E-27 | 53.41234335 |
| C6orf15 | 1.961194788 | 0.664375298 | 11.72512068 | 2.81E-28 | 6.40E-27 | 53.36165941 |
| AGR3 | 1.204370364 | 0.363805288 | 11.71734994 | 3.02E-28 | 6.87E-27 | 53.2901436 |
| KIF18A | 1.68523899 | 4.117694471 | 11.7155237 | 3.07E-28 | 6.98E-27 | 53.27334021 |
| CDK5R1 | -1.16716622 | 10.751471 | -11.69697057 | 3.65E-28 | 8.25E-27 | 53.10271691 |
| TOM1L1 | 2.094590259 | 4.576419014 | 11.69132972 | 3.85E-28 | 8.68E-27 | 53.05087187 |
| ARHGDIG | -1.864830692 | 8.024317583 | -11.69011964 | 3.89E-28 | 8.76E-27 | 53.03975184 |
| CASP1 | 1.297569422 | 6.946321786 | 11.68684967 | 4.01E-28 | 9.03E-27 | 53.00970596 |
| SKAP2 | 1.248463794 | 8.483023633 | 11.67489231 | 4.48E-28 | 1.01E-26 | 52.89987755 |
| FXYD6 | -1.080591389 | 14.23267855 | -11.66979955 | 4.70E-28 | 1.05E-26 | 52.85312024 |
| HLA-DPB1 | 1.762764176 | 9.815819046 | 11.66679242 | 4.83E-28 | 1.08E-26 | 52.82551685 |
| F2RL2 | 1.988540191 | 3.314408711 | 11.66166816 | 5.06E-28 | 1.13E-26 | 52.7784892 |
| P2RX6 | -1.221852433 | 7.648885476 | -11.64381352 | 5.98E-28 | 1.33E-26 | 52.61472209 |
| ECM2 | 1.664650808 | 7.919395752 | 11.62207188 | 7.31E-28 | 1.62E-26 | 52.41549833 |
| LOC283314 | 1.061805411 | 2.719251831 | 11.61743504 | 7.63E-28 | 1.68E-26 | 52.37303776 |
| NDN | -1.092423835 | 10.17741918 | -11.60802291 | 8.32E-28 | 1.83E-26 | 52.28687909 |
| GRIK2 | -1.353739361 | 9.795902345 | -11.60693382 | 8.41E-28 | 1.84E-26 | 52.27691213 |
| EPHB1 | -1.373560009 | 10.48239239 | -11.59485744 | 9.40E-28 | 2.06E-26 | 52.1664302 |
| HOXC4 | 2.024736631 | 4.080668433 | 11.5855555 | 1.02E-27 | 2.24E-26 | 52.08137598 |
| CCDC8 | 1.566058747 | 5.522596142 | 11.57989256 | 1.08E-27 | 2.35E-26 | 52.02961516 |
| ALOX5AP | 1.588037539 | 7.961696898 | 11.57987754 | 1.08E-27 | 2.35E-26 | 52.02947785 |
| PTRF | 1.187619467 | 10.10269733 | 11.57883684 | 1.09E-27 | 2.37E-26 | 52.01996715 |
| BCAT1 | 1.503847383 | 8.403467241 | 11.57714198 | 1.11E-27 | 2.40E-26 | 52.00447935 |
| TCTEX1D4 | 1.031921604 | 1.005517372 | 11.57665009 | 1.11E-27 | 2.41E-26 | 51.9999847 |
| BRSK2 | -1.119186551 | 9.51504059 | -11.57360535 | 1.14E-27 | 2.48E-26 | 51.97216552 |
| MAST1 | -1.302756417 | 9.616518706 | -11.56687362 | 1.22E-27 | 2.63E-26 | 51.91067427 |
| GPR61 | -1.548814749 | 4.630924506 | -11.55745191 | 1.33E-27 | 2.86E-26 | 51.82464623 |
| CALN1 | -2.202509615 | 9.300183253 | -11.55678934 | 1.34E-27 | 2.87E-26 | 51.81859802 |
| PLEK2 | 1.447752692 | 2.125425723 | 11.55371799 | 1.37E-27 | 2.95E-26 | 51.7905638 |
| CDH22 | -1.900818011 | 7.575315179 | -11.54818744 | 1.45E-27 | 3.10E-26 | 51.7400938 |
| SAMD11 | -1.861220852 | 6.802115891 | -11.54795055 | 1.45E-27 | 3.10E-26 | 51.73793236 |
| SLC11A1 | 1.833248761 | 7.697548447 | 11.5476089 | 1.45E-27 | 3.11E-26 | 51.73481508 |
| SVOP | -2.655574954 | 7.949635774 | -11.54295587 | 1.52E-27 | 3.24E-26 | 51.69236523 |
| BZRAP1 | -1.086500307 | 10.64589511 | -11.54174618 | 1.54E-27 | 3.27E-26 | 51.68133078 |
| PCOLCE | 1.333407805 | 6.800200662 | 11.52919418 | 1.72E-27 | 3.66E-26 | 51.56687505 |
| COL6A2 | 1.621381467 | 8.374349085 | 11.5241494 | 1.81E-27 | 3.82E-26 | 51.52089453 |
| TRIM17 | -1.289093001 | 5.177895284 | -11.52181741 | 1.84E-27 | 3.90E-26 | 51.4996437 |
| CISH | 1.245865156 | 4.714632428 | 11.51847443 | 1.90E-27 | 4.01E-26 | 51.46918432 |
| SHD | -2.081272066 | 10.54200579 | -11.51564455 | 1.95E-27 | 4.11E-26 | 51.44340403 |
| LOC441666 | -1.749675641 | 5.912700391 | -11.51555536 | 1.95E-27 | 4.11E-26 | 51.44259163 |
| COL1A2 | 1.531043762 | 9.291494347 | 11.50671401 | 2.12E-27 | 4.44E-26 | 51.362071 |
| LOC400759 | 1.503095964 | 2.67723106 | 11.50148904 | 2.22E-27 | 4.65E-26 | 51.31450281 |
| MARVELD1 | 1.125536177 | 6.911519184 | 11.49530959 | 2.35E-27 | 4.91E-26 | 51.25826135 |
| CMTM5 | -1.273928506 | 9.764293818 | -11.49473653 | 2.37E-27 | 4.93E-26 | 51.2530466 |
| HOXD9 | 2.646878962 | 2.368084653 | 11.49116741 | 2.45E-27 | 5.09E-26 | 51.22057171 |
| KIF21B | -1.070035862 | 11.35885275 | -11.48545101 | 2.58E-27 | 5.36E-26 | 51.16857148 |
| HTRA3 | 1.717109647 | 4.181471914 | 11.48187614 | 2.66E-27 | 5.53E-26 | 51.13605972 |
| C1S | 1.444990935 | 9.795920567 | 11.48006595 | 2.71E-27 | 5.62E-26 | 51.11959916 |
| PRKCE | -1.04358243 | 9.449983413 | -11.46477386 | 3.12E-27 | 6.43E-26 | 50.98060479 |
| RGS11 | -1.359874929 | 9.083744919 | -11.46439382 | 3.13E-27 | 6.45E-26 | 50.97715191 |
| VDR | 1.338487786 | 2.993745624 | 11.4594842 | 3.27E-27 | 6.73E-26 | 50.93255091 |
| APOL4 | 2.154550002 | 6.467512747 | 11.45582699 | 3.38E-27 | 6.95E-26 | 50.8993346 |
| GABRD | -2.051240312 | 8.161180933 | -11.4369007 | 4.03E-27 | 8.25E-26 | 50.72753793 |
| S100A6 | 1.234232083 | 11.38227848 | 11.43514077 | 4.09E-27 | 8.37E-26 | 50.71157133 |
| CYP1A1 | -1.349935057 | 2.627313005 | -11.43506734 | 4.09E-27 | 8.37E-26 | 50.71090516 |
| C9orf122 | -1.68596203 | 4.778558141 | -11.43199396 | 4.21E-27 | 8.58E-26 | 50.68302611 |
| SPAG17 | 1.793539986 | 1.331889666 | 11.41460286 | 4.94E-27 | 1.00E-25 | 50.52535234 |
| SLC8A2 | -1.968263851 | 8.409500315 | -11.40904382 | 5.20E-27 | 1.05E-25 | 50.47498205 |
| AVIL | 1.257764116 | 5.352225547 | 11.40789483 | 5.25E-27 | 1.06E-25 | 50.46457291 |
| MYOF | 1.203998304 | 8.511427935 | 11.38758267 | 6.32E-27 | 1.27E-25 | 50.28065942 |
| TNNT1 | -2.241413666 | 4.749500794 | -11.38286147 | 6.60E-27 | 1.32E-25 | 50.23793987 |
| FAM159A | 1.198452661 | 2.763242806 | 11.37537927 | 7.07E-27 | 1.41E-25 | 50.17025897 |
| RTN4RL2 | -1.409060391 | 6.784069196 | -11.37230032 | 7.27E-27 | 1.45E-25 | 50.14241573 |
| PTGS1 | 1.314134658 | 8.263083065 | 11.37146392 | 7.33E-27 | 1.46E-25 | 50.13485287 |
| MYT1 | -1.806236239 | 9.437336473 | -11.36867467 | 7.52E-27 | 1.49E-25 | 50.10963433 |
| HOXB2 | 1.911022192 | 2.871355706 | 11.35789507 | 8.30E-27 | 1.65E-25 | 50.01220709 |
| BASP1 | -1.085515342 | 11.1612964 | -11.35752832 | 8.32E-27 | 1.65E-25 | 50.00889335 |
| CELF5 | -1.575908103 | 9.264448247 | -11.34901641 | 9.00E-27 | 1.78E-25 | 49.93200201 |
| TRIM5 | 1.018712974 | 7.517117272 | 11.34684237 | 9.18E-27 | 1.81E-25 | 49.91236855 |
| ZNF474 | 1.447034536 | 2.569532975 | 11.34455408 | 9.37E-27 | 1.84E-25 | 49.89170578 |
| TNK2 | -1.027614701 | 12.58140167 | -11.33857043 | 9.90E-27 | 1.94E-25 | 49.83768629 |
| CD2 | 1.802332902 | 2.862268182 | 11.33217311 | 1.05E-26 | 2.05E-25 | 49.77995111 |
| MICALL2 | 1.078760488 | 8.363051854 | 11.32806936 | 1.09E-26 | 2.12E-25 | 49.74292535 |
| EVC | 1.413054101 | 6.523622153 | 11.32051493 | 1.17E-26 | 2.27E-25 | 49.67478704 |
| KCNQ5 | -1.618940201 | 8.341537076 | -11.31413868 | 1.24E-26 | 2.40E-25 | 49.61729661 |
| WISP1 | 1.848101691 | 3.239915538 | 11.31214845 | 1.26E-26 | 2.45E-25 | 49.59935602 |
| CD109 | 1.199125404 | 7.240671874 | 11.30559376 | 1.34E-26 | 2.59E-25 | 49.54028304 |
| TSPYL2 | -1.031244229 | 11.1505802 | -11.30292053 | 1.37E-26 | 2.65E-25 | 49.51619689 |
| MGAT4C | -1.712826595 | 6.486780822 | -11.30007009 | 1.41E-26 | 2.71E-25 | 49.49051789 |
| ADAM29 | -1.508472513 | 2.245499314 | -11.29424444 | 1.48E-26 | 2.85E-25 | 49.43804775 |
| SLC25A41 | -1.048502816 | 4.543338576 | -11.2927654 | 1.50E-26 | 2.89E-25 | 49.42472901 |
| LINGO1 | -1.204346356 | 11.34148349 | -11.28927533 | 1.55E-26 | 2.98E-25 | 49.39330502 |
| HLF | -1.031841691 | 9.75107515 | -11.28200841 | 1.66E-26 | 3.17E-25 | 49.32789362 |
| RBP3 | -1.646314417 | 3.850744167 | -11.2771585 | 1.73E-26 | 3.30E-25 | 49.28425246 |
| FCGBP | 2.218750778 | 10.05142057 | 11.2614746 | 2.00E-26 | 3.81E-25 | 49.14320007 |
| SOX8 | -1.519992918 | 13.53435065 | -11.25831646 | 2.06E-26 | 3.91E-25 | 49.11481174 |
| HLA-DRB1 | 1.777768454 | 9.942186528 | 11.25705209 | 2.08E-26 | 3.95E-25 | 49.10344767 |
| LGR5 | -1.577488529 | 7.232796554 | -11.24661645 | 2.29E-26 | 4.33E-25 | 49.00968258 |
| ADCY5 | -1.04368822 | 8.689419672 | -11.24032213 | 2.42E-26 | 4.57E-25 | 48.95315286 |
| B3GNT7 | 1.460133175 | 5.559557831 | 11.23999675 | 2.43E-26 | 4.58E-25 | 48.95023112 |
| DGKK | -2.584066415 | 3.304773874 | -11.2354403 | 2.53E-26 | 4.76E-25 | 48.9093219 |
| DNALI1 | 1.041596696 | 9.101306301 | 11.23232934 | 2.60E-26 | 4.89E-25 | 48.88139651 |
| HMGCLL1 | -1.805457723 | 6.165217453 | -11.22822424 | 2.70E-26 | 5.07E-25 | 48.8445543 |
| LOXL4 | 1.478616349 | 3.382249303 | 11.21241777 | 3.12E-26 | 5.82E-25 | 48.70277118 |
| TACC3 | 1.306407246 | 7.821209605 | 11.20804762 | 3.24E-26 | 6.05E-25 | 48.66359243 |
| IL7 | 1.132436898 | 3.004221981 | 11.20548668 | 3.32E-26 | 6.18E-25 | 48.64063763 |
| TMEM100 | -1.801737685 | 10.28476875 | -11.20227396 | 3.42E-26 | 6.35E-25 | 48.61184516 |
| FUCA2 | 1.013900737 | 8.702503809 | 11.19916088 | 3.52E-26 | 6.52E-25 | 48.58395038 |
| PCDH15 | -2.176538931 | 9.487832529 | -11.19275415 | 3.73E-26 | 6.90E-25 | 48.52655763 |
| RDM1 | 1.242875248 | 1.309631375 | 11.19087787 | 3.79E-26 | 7.00E-25 | 48.50975325 |
| LRRTM3 | -1.158114238 | 9.30845782 | -11.18635564 | 3.95E-26 | 7.28E-25 | 48.4692583 |
| SRRM3 | -1.614121693 | 8.535972474 | -11.18347266 | 4.05E-26 | 7.47E-25 | 48.44344735 |
| C2orf70 | 1.128825109 | 0.804083252 | 11.17992096 | 4.18E-26 | 7.70E-25 | 48.411655 |
| TNFSF14 | 1.106313626 | 0.933955618 | 11.17738261 | 4.28E-26 | 7.87E-25 | 48.38893715 |
| TCIRG1 | 1.007459015 | 7.911190173 | 11.16628242 | 4.73E-26 | 8.66E-25 | 48.28962894 |
| TDH | -1.533955428 | 4.605537815 | -11.16525808 | 4.78E-26 | 8.73E-25 | 48.28046767 |
| 11-Mar | -1.853069561 | 2.876236626 | -11.15566288 | 5.21E-26 | 9.49E-25 | 48.19467676 |
| FAM101A | -1.098067577 | 6.08890591 | -11.14762212 | 5.60E-26 | 1.02E-24 | 48.12281859 |
| RP1-177G6.2 | -1.40240329 | 4.621611743 | -11.14461964 | 5.76E-26 | 1.04E-24 | 48.09599426 |
| ZNF804A | -1.456475458 | 7.244823259 | -11.13596632 | 6.23E-26 | 1.13E-24 | 48.01870949 |
| TNFRSF19 | 1.287901338 | 8.563978567 | 11.13377631 | 6.35E-26 | 1.15E-24 | 47.99915573 |
| SEC61G | 1.206007456 | 9.315120773 | 11.13250166 | 6.42E-26 | 1.16E-24 | 47.98777606 |
| RSPH4A | 1.396065245 | 4.605768418 | 11.12133292 | 7.10E-26 | 1.28E-24 | 47.88809821 |
| TNFAIP8 | 1.208919006 | 4.822784386 | 11.12124166 | 7.11E-26 | 1.28E-24 | 47.88728396 |
| XKR7 | -2.313479197 | 3.815342303 | -11.11794967 | 7.33E-26 | 1.32E-24 | 47.85791559 |
| DUSP10 | 1.036609826 | 6.881435435 | 11.11394704 | 7.59E-26 | 1.36E-24 | 47.82221465 |
| MAGEL2 | -1.185398042 | 6.559887358 | -11.11221561 | 7.71E-26 | 1.38E-24 | 47.80677384 |
| MAB21L2 | 1.362126102 | 0.971779192 | 11.1108571 | 7.81E-26 | 1.40E-24 | 47.79465968 |
| F3 | 1.448426076 | 9.951258335 | 11.10519955 | 8.22E-26 | 1.47E-24 | 47.74421978 |
| MCF2 | -1.757384359 | 6.206459703 | -11.100315 | 8.59E-26 | 1.53E-24 | 47.70068419 |
| HLA-DMA | 1.329717791 | 9.017170441 | 11.07553392 | 1.07E-25 | 1.90E-24 | 47.47999197 |
| DPEP1 | 2.470060742 | 2.884513309 | 11.06837255 | 1.14E-25 | 2.02E-24 | 47.41627125 |
| AMZ1 | -1.403575723 | 7.516849216 | -11.0677544 | 1.15E-25 | 2.03E-24 | 47.41077224 |
| EYA4 | 2.142296051 | 4.001593637 | 11.06488551 | 1.18E-25 | 2.08E-24 | 47.38525317 |
| ACCN2 | -1.018510495 | 11.42530572 | -11.06217407 | 1.21E-25 | 2.13E-24 | 47.36113843 |
| FBXO17 | 1.635547578 | 6.273127319 | 11.0611821 | 1.22E-25 | 2.15E-24 | 47.35231703 |
| FKBP9L | 1.059313392 | 4.235839788 | 11.05466778 | 1.30E-25 | 2.27E-24 | 47.2943985 |
| CNTNAP2 | -1.684925288 | 9.362539651 | -11.0522953 | 1.32E-25 | 2.32E-24 | 47.27331004 |
| ELN | 1.090611513 | 10.17984716 | 11.04979007 | 1.35E-25 | 2.37E-24 | 47.25104462 |
| GPR158 | -1.226847332 | 10.03497852 | -11.04885301 | 1.36E-25 | 2.39E-24 | 47.24271728 |
| G0S2 | 1.934073352 | 3.231161838 | 11.04821388 | 1.37E-25 | 2.40E-24 | 47.23703773 |
| RTN4R | -1.449452673 | 7.607052235 | -11.04502307 | 1.41E-25 | 2.47E-24 | 47.20868615 |
| UPK2 | -1.329179392 | 3.918873627 | -11.04226983 | 1.45E-25 | 2.53E-24 | 47.18422648 |
| PTPN7 | 1.712034675 | 4.602586191 | 11.03925543 | 1.49E-25 | 2.59E-24 | 47.15745107 |
| PLAC8 | 1.915395246 | 3.090758903 | 11.03076032 | 1.61E-25 | 2.79E-24 | 47.08201724 |
| HOXB8 | 1.474859151 | 0.565020421 | 11.03040262 | 1.61E-25 | 2.80E-24 | 47.07884177 |
| DENND2D | 1.424679382 | 4.990233297 | 11.02896266 | 1.63E-25 | 2.83E-24 | 47.06605918 |
| CFI | 1.627236892 | 7.621287434 | 11.02204594 | 1.74E-25 | 2.99E-24 | 47.00467332 |
| S1PR3 | 1.391667551 | 9.103586133 | 11.0132809 | 1.88E-25 | 3.23E-24 | 46.92691763 |
| IGFBP5 | 1.293487022 | 12.30099337 | 11.00731578 | 1.98E-25 | 3.40E-24 | 46.87402207 |
| AEBP1 | 2.102647178 | 10.36683626 | 11.00600561 | 2.00E-25 | 3.44E-24 | 46.86240652 |
| C6orf150 | 1.004063696 | 3.880646384 | 10.99995525 | 2.12E-25 | 3.63E-24 | 46.80877699 |
| C12orf34 | -1.110797901 | 10.14308746 | -10.99402902 | 2.23E-25 | 3.82E-24 | 46.7562653 |
| CDCA7L | 1.197607809 | 8.262793447 | 10.98635455 | 2.39E-25 | 4.08E-24 | 46.68828854 |
| ICOS | 1.016310913 | 0.819463351 | 10.98601783 | 2.40E-25 | 4.09E-24 | 46.68530673 |
| TNFRSF1A | 1.01966073 | 10.01509026 | 10.9860026 | 2.40E-25 | 4.09E-24 | 46.68517179 |
| TRIM22 | 1.067793373 | 9.379656657 | 10.98151393 | 2.50E-25 | 4.25E-24 | 46.64542768 |
| USH1C | -2.36461247 | 8.658467669 | -10.9734184 | 2.68E-25 | 4.56E-24 | 46.57377244 |
| CFH | 1.197341041 | 7.790383579 | 10.97222674 | 2.71E-25 | 4.61E-24 | 46.56322757 |
| FKBP10 | 1.063827106 | 10.3557289 | 10.97060312 | 2.75E-25 | 4.67E-24 | 46.5488614 |
| TMEM63C | -1.104001692 | 8.567345017 | -10.96995942 | 2.77E-25 | 4.69E-24 | 46.54316619 |
| PTCRA | 1.451317326 | 2.104784998 | 10.96472849 | 2.90E-25 | 4.91E-24 | 46.4968925 |
| HTR1A | -2.108772633 | 3.166040738 | -10.95642222 | 3.12E-25 | 5.28E-24 | 46.42344171 |
| HLA-B | 1.030250984 | 12.94738042 | 10.94724068 | 3.39E-25 | 5.71E-24 | 46.34229101 |
| RPRM | -1.588228361 | 8.495427948 | -10.94413433 | 3.49E-25 | 5.86E-24 | 46.31484517 |
| LOC100133545 | -1.351632191 | 4.323422565 | -10.93468458 | 3.80E-25 | 6.35E-24 | 46.23138249 |
| TCTEX1D1 | 2.225858093 | 3.665051231 | 10.93117441 | 3.92E-25 | 6.55E-24 | 46.20039112 |
| RBP1 | 1.797856149 | 7.637862201 | 10.92844689 | 4.01E-25 | 6.71E-24 | 46.17631398 |
| POSTN | 3.120047265 | 3.110253959 | 10.92721586 | 4.06E-25 | 6.78E-24 | 46.16544835 |
| MMP2 | 1.116133066 | 9.74359556 | 10.92594951 | 4.10E-25 | 6.85E-24 | 46.15427169 |
| CHAF1B | 1.20853074 | 5.734744118 | 10.9185882 | 4.38E-25 | 7.27E-24 | 46.08931769 |
| ATP1A3 | -1.356538566 | 12.2271995 | -10.91801815 | 4.40E-25 | 7.30E-24 | 46.08428882 |
| C7orf57 | 2.197594276 | 2.631215608 | 10.91674178 | 4.45E-25 | 7.37E-24 | 46.07302964 |
| GRIA2 | -1.116360215 | 11.69034323 | -10.90995768 | 4.73E-25 | 7.82E-24 | 46.01319905 |
| FSTL5 | -2.168037251 | 7.093297319 | -10.90915505 | 4.77E-25 | 7.87E-24 | 46.006122 |
| PARP15 | 1.185456145 | 2.082778188 | 10.90774265 | 4.83E-25 | 7.97E-24 | 45.99366915 |
| MAGEE1 | -1.109477784 | 8.650414386 | -10.90069237 | 5.14E-25 | 8.48E-24 | 45.93152325 |
| HSPG2 | 1.338338628 | 9.056835363 | 10.89159017 | 5.57E-25 | 9.17E-24 | 45.85132727 |
| BST2 | 1.168241262 | 9.182379905 | 10.8900435 | 5.65E-25 | 9.29E-24 | 45.83770426 |
| KCTD16 | -1.306223242 | 5.881719357 | -10.88361718 | 5.98E-25 | 9.83E-24 | 45.78111447 |
| CDC42BPG | 1.004799357 | 1.735718413 | 10.88130256 | 6.11E-25 | 1.00E-23 | 45.76073718 |
| NKAIN1 | -1.427680295 | 8.405655145 | -10.88050894 | 6.15E-25 | 1.01E-23 | 45.75375096 |
| PON1 | -1.695052495 | 3.618234164 | -10.88004203 | 6.18E-25 | 1.01E-23 | 45.74964091 |
| UNC93B1 | 1.06968592 | 8.126674559 | 10.87801331 | 6.29E-25 | 1.03E-23 | 45.73178407 |
| PABPC4L | 1.043166761 | 3.356826461 | 10.87769302 | 6.31E-25 | 1.03E-23 | 45.72896503 |
| FAM19A3 | 1.305349767 | 1.782964426 | 10.8772923 | 6.33E-25 | 1.03E-23 | 45.72543825 |
| LYVE1 | -1.954183384 | 6.829715013 | -10.87665496 | 6.37E-25 | 1.04E-23 | 45.71982899 |
| LOX | 1.22732617 | 5.402818368 | 10.87622138 | 6.39E-25 | 1.04E-23 | 45.71601321 |
| AGBL2 | 1.383396763 | 2.507521678 | 10.8761365 | 6.40E-25 | 1.04E-23 | 45.71526618 |
| GAPT | 1.648148847 | 4.243296118 | 10.86614775 | 6.99E-25 | 1.14E-23 | 45.62738462 |
| CCDC85A | -1.221449204 | 7.821377556 | -10.86126876 | 7.30E-25 | 1.18E-23 | 45.58447728 |
| ASF1B | 1.617453156 | 5.859155183 | 10.85587644 | 7.66E-25 | 1.24E-23 | 45.5370695 |
| KCNH1 | -1.488072982 | 7.937645818 | -10.85479588 | 7.73E-25 | 1.25E-23 | 45.52757123 |
| CCR5 | 1.563385297 | 5.00298936 | 10.84593752 | 8.37E-25 | 1.35E-23 | 45.44972765 |
| HTR2A | -1.811448826 | 5.845219779 | -10.8450288 | 8.44E-25 | 1.36E-23 | 45.4417444 |
| KIF23 | 1.774017302 | 5.21292781 | 10.83962654 | 8.85E-25 | 1.42E-23 | 45.39429357 |
| NEGR1 | -1.346184854 | 9.007704538 | -10.83448883 | 9.26E-25 | 1.48E-23 | 45.34917998 |
| SPATA17 | 1.394411751 | 3.250160585 | 10.83390745 | 9.31E-25 | 1.49E-23 | 45.34407581 |
| SYN3 | -1.429024797 | 6.575911128 | -10.83306402 | 9.38E-25 | 1.50E-23 | 45.33667128 |
| PRSS35 | -1.709422103 | 7.581381563 | -10.83140381 | 9.52E-25 | 1.52E-23 | 45.32209725 |
| KCNJ9 | -1.346194042 | 8.940927603 | -10.83067852 | 9.58E-25 | 1.53E-23 | 45.31573077 |
| FAM183A | 1.822918052 | 1.009207793 | 10.82743559 | 9.86E-25 | 1.57E-23 | 45.28726819 |
| GATA3 | 1.434046037 | 2.156250952 | 10.82191655 | 1.04E-24 | 1.65E-23 | 45.23884081 |
| KLHL35 | -1.462457884 | 4.886383325 | -10.8212928 | 1.04E-24 | 1.65E-23 | 45.23336867 |
| SLC34A2 | 2.004652136 | 2.575084338 | 10.82085551 | 1.05E-24 | 1.66E-23 | 45.22953244 |
| EMR2 | 1.322715694 | 5.783704715 | 10.81646467 | 1.09E-24 | 1.72E-23 | 45.19101786 |
| OAS1 | 1.352820096 | 7.483687437 | 10.80782309 | 1.17E-24 | 1.85E-23 | 45.11524632 |
| HLA-DQA1 | 2.267026414 | 7.327436293 | 10.80275644 | 1.23E-24 | 1.94E-23 | 45.07083834 |
| SCN11A | -1.015195926 | 3.930289777 | -10.80039907 | 1.25E-24 | 1.97E-23 | 45.05018091 |
| FCGR2A | 1.329861041 | 8.689902602 | 10.79764708 | 1.28E-24 | 2.02E-23 | 45.02606912 |
| LAMB4 | 1.13532677 | 1.855481257 | 10.78777539 | 1.40E-24 | 2.20E-23 | 44.93960899 |
| ACCN4 | -1.950894757 | 10.26697386 | -10.78570102 | 1.43E-24 | 2.24E-23 | 44.92144716 |
| PDLIM4 | 1.893659191 | 6.111062031 | 10.78545689 | 1.43E-24 | 2.24E-23 | 44.9193099 |
| TEAD3 | 1.195175206 | 5.715266063 | 10.77183549 | 1.61E-24 | 2.53E-23 | 44.80010583 |
| RUNX1T1 | -1.304150567 | 6.245237533 | -10.76838118 | 1.66E-24 | 2.60E-23 | 44.76989141 |
| ARL4C | 1.221563047 | 9.480540888 | 10.76502366 | 1.71E-24 | 2.68E-23 | 44.7405294 |
| PPP1R1A | -1.696303244 | 8.194141865 | -10.76479324 | 1.72E-24 | 2.68E-23 | 44.73851449 |
| RGS16 | 1.46932794 | 7.070649784 | 10.76461188 | 1.72E-24 | 2.68E-23 | 44.7369287 |
| E2F8 | 1.6119083 | 3.391641365 | 10.76266512 | 1.75E-24 | 2.73E-23 | 44.71990704 |
| C13orf26 | 1.922253414 | 2.437580112 | 10.74986979 | 1.96E-24 | 3.04E-23 | 44.60807809 |
| PLAUR | 1.245818751 | 6.368842194 | 10.74676278 | 2.02E-24 | 3.12E-23 | 44.58093605 |
| SOCS2 | 1.28602295 | 7.460634172 | 10.74562362 | 2.04E-24 | 3.15E-23 | 44.57098582 |
| PLCXD2 | -1.199240019 | 7.263594445 | -10.74547486 | 2.04E-24 | 3.16E-23 | 44.56968649 |
| C20orf203 | -1.262428405 | 4.239410545 | -10.74368156 | 2.07E-24 | 3.20E-23 | 44.55402408 |
| CDCA2 | 1.649821929 | 4.09299812 | 10.73571792 | 2.22E-24 | 3.43E-23 | 44.48449072 |
| LAMA2 | 1.178605165 | 8.174116906 | 10.72923679 | 2.35E-24 | 3.62E-23 | 44.42792563 |
| C3 | 1.591959492 | 12.66413264 | 10.69901024 | 3.07E-24 | 4.68E-23 | 44.16440311 |
| DBC1 | -1.594741406 | 9.525723743 | -10.69829296 | 3.09E-24 | 4.71E-23 | 44.15815537 |
| LOC158696 | -1.649897453 | 4.376596957 | -10.69511467 | 3.18E-24 | 4.84E-23 | 44.13047468 |
| CELSR3 | -1.31261667 | 9.844254996 | -10.69376547 | 3.22E-24 | 4.89E-23 | 44.11872566 |
| PGCP | 1.02292274 | 7.793637881 | 10.68647097 | 3.43E-24 | 5.20E-23 | 44.05522037 |
| SPOCD1 | 2.477929665 | 6.607040679 | 10.68449325 | 3.49E-24 | 5.28E-23 | 44.03800728 |
| HLA-DMB | 1.332594249 | 9.009489867 | 10.6834182 | 3.52E-24 | 5.33E-23 | 44.02865137 |
| ABCA13 | 1.599015473 | 2.829809911 | 10.68058226 | 3.61E-24 | 5.46E-23 | 44.00397375 |
| DERL3 | 1.146655984 | 4.380399704 | 10.6698118 | 3.97E-24 | 5.97E-23 | 43.91028991 |
| 4-Mar | -2.323549739 | 6.829711784 | -10.66189762 | 4.26E-24 | 6.40E-23 | 43.84148875 |
| TMEM61 | 1.197576107 | 1.306559098 | 10.66122392 | 4.28E-24 | 6.43E-23 | 43.83563352 |
| DDX25 | -1.3405853 | 8.071359868 | -10.6611918 | 4.28E-24 | 6.43E-23 | 43.8353544 |
| OPHN1 | -1.034615442 | 10.95447543 | -10.65941714 | 4.35E-24 | 6.53E-23 | 43.81993163 |
| PNCK | -1.406403245 | 7.642142375 | -10.65344131 | 4.59E-24 | 6.86E-23 | 43.76801039 |
| LRFN5 | -1.836410455 | 6.667638423 | -10.64316526 | 5.02E-24 | 7.49E-23 | 43.67876979 |
| MEGF11 | -1.500920586 | 9.773507876 | -10.6416133 | 5.09E-24 | 7.58E-23 | 43.66529678 |
| EPSTI1 | 1.271216818 | 5.604421092 | 10.64011103 | 5.16E-24 | 7.67E-23 | 43.65225644 |
| PDGFA | 1.124714023 | 8.741729171 | 10.63059574 | 5.61E-24 | 8.32E-23 | 43.56968631 |
| RASGEF1C | -1.138663795 | 8.163812147 | -10.62114325 | 6.09E-24 | 9.03E-23 | 43.48770757 |
| SLIT1 | -1.537959241 | 11.85344802 | -10.62029745 | 6.14E-24 | 9.09E-23 | 43.4803745 |
| MX1 | 1.210054775 | 9.293356824 | 10.6181682 | 6.25E-24 | 9.25E-23 | 43.46191543 |
| NDST4 | -2.219083127 | 3.377720542 | -10.61771174 | 6.28E-24 | 9.28E-23 | 43.45795863 |
| TUBB6 | 1.102703074 | 8.737905768 | 10.61576983 | 6.38E-24 | 9.44E-23 | 43.44112619 |
| RARRES2 | 1.948274612 | 5.847168423 | 10.61229864 | 6.58E-24 | 9.71E-23 | 43.41104291 |
| KLHL32 | -1.12979738 | 8.96727513 | -10.60821361 | 6.82E-24 | 1.00E-22 | 43.37564778 |
| SLC7A14 | -1.630387953 | 9.289004597 | -10.59923496 | 7.38E-24 | 1.08E-22 | 43.29788184 |
| CPLX1 | -1.60333734 | 9.189821451 | -10.59595092 | 7.60E-24 | 1.11E-22 | 43.26944857 |
| CTF1 | 1.078854518 | 5.500838125 | 10.59229734 | 7.84E-24 | 1.15E-22 | 43.23782245 |
| SPP1 | 1.679520514 | 12.7611572 | 10.58788959 | 8.15E-24 | 1.19E-22 | 43.19967737 |
| ELF4 | 1.104494854 | 6.177255985 | 10.58622896 | 8.27E-24 | 1.21E-22 | 43.1853087 |
| ATRNL1 | -1.249558273 | 9.317583458 | -10.575501 | 9.09E-24 | 1.32E-22 | 43.09251945 |
| ABCA17P | -1.073578354 | 5.648767324 | -10.57440406 | 9.17E-24 | 1.33E-22 | 43.0830351 |
| ASPM | 2.031104823 | 5.557361633 | 10.57146576 | 9.41E-24 | 1.37E-22 | 43.057633 |
| GDF15 | 1.910362168 | 3.720245447 | 10.57049305 | 9.49E-24 | 1.38E-22 | 43.04922479 |
| GPR27 | -1.521382215 | 4.445812979 | -10.56897334 | 9.62E-24 | 1.39E-22 | 43.0360892 |
| WDR62 | 1.412816546 | 5.868594026 | 10.56863571 | 9.65E-24 | 1.40E-22 | 43.03317105 |
| IL1RAP | 1.281801011 | 8.149111106 | 10.56245356 | 1.02E-23 | 1.47E-22 | 42.97974931 |
| AURKA | 1.055412533 | 6.022509412 | 10.56199681 | 1.02E-23 | 1.48E-22 | 42.97580318 |
| VSTM2B | -1.463907701 | 7.177376128 | -10.55831053 | 1.06E-23 | 1.52E-22 | 42.94395931 |
| SLC16A4 | 1.057771776 | 7.544105788 | 10.55360244 | 1.10E-23 | 1.58E-22 | 42.903299 |
| FZD2 | 1.044694741 | 5.887645662 | 10.54724201 | 1.16E-23 | 1.67E-22 | 42.848387 |
| CRHR1 | -1.524714912 | 5.526426946 | -10.54311054 | 1.21E-23 | 1.73E-22 | 42.81272975 |
| GRIA4 | -1.182441252 | 11.61276311 | -10.5331753 | 1.32E-23 | 1.88E-22 | 42.72701895 |
| PHACTR3 | -1.101749802 | 10.42484321 | -10.53257702 | 1.32E-23 | 1.89E-22 | 42.7218593 |
| WIT1 | 1.02962863 | 0.417081572 | 10.53089744 | 1.34E-23 | 1.92E-22 | 42.70737529 |
| DEGS2 | -1.027452043 | 5.671828271 | -10.52904443 | 1.36E-23 | 1.94E-22 | 42.69139745 |
| LAMB1 | 1.339263792 | 8.59324897 | 10.5276254 | 1.38E-23 | 1.97E-22 | 42.67916286 |
| CXCR6 | 1.456916089 | 2.009645655 | 10.52400311 | 1.42E-23 | 2.02E-22 | 42.64793695 |
| CLEC17A | 1.744820211 | 2.807804012 | 10.51776385 | 1.50E-23 | 2.13E-22 | 42.59416776 |
| CENPF | 1.690880855 | 8.081176377 | 10.51075084 | 1.60E-23 | 2.26E-22 | 42.53375488 |
| CABP1 | -1.882603442 | 7.315864471 | -10.51070235 | 1.60E-23 | 2.26E-22 | 42.53333728 |
| GCOM1 | 1.611402354 | 4.770169509 | 10.50591112 | 1.67E-23 | 2.35E-22 | 42.49207869 |
| MORN3 | 1.433415648 | 3.153450307 | 10.50065975 | 1.75E-23 | 2.46E-22 | 42.44687162 |
| SOCS1 | 1.14351686 | 3.983674311 | 10.49068165 | 1.90E-23 | 2.68E-22 | 42.3610139 |
| RICH2 | -1.447694171 | 7.835952936 | -10.48842659 | 1.94E-23 | 2.73E-22 | 42.34161724 |
| CEP55 | 1.709662429 | 4.541181703 | 10.48765963 | 1.96E-23 | 2.74E-22 | 42.33502097 |
| C11orf21 | 1.048810462 | 2.196013682 | 10.48682392 | 1.97E-23 | 2.76E-22 | 42.32783374 |
| FOXJ1 | 1.669825535 | 7.261642979 | 10.48460612 | 2.01E-23 | 2.81E-22 | 42.30876205 |
| MKX | -1.904224193 | 6.588696833 | -10.48311412 | 2.03E-23 | 2.85E-22 | 42.29593322 |
| CCDC103 | 1.041618478 | 8.939764661 | 10.4787422 | 2.11E-23 | 2.95E-22 | 42.25834857 |
| LHX5 | -2.77446109 | 3.475159543 | -10.47300802 | 2.22E-23 | 3.10E-22 | 42.20906812 |
| CUBN | 1.033301596 | 5.666277381 | 10.45288722 | 2.65E-23 | 3.67E-22 | 42.03628412 |
| DLGAP3 | -1.428307423 | 7.854947544 | -10.45143182 | 2.68E-23 | 3.71E-22 | 42.02379448 |
| CENPA | 1.659657809 | 3.772624653 | 10.45084406 | 2.69E-23 | 3.73E-22 | 42.01875087 |
| C9orf47 | 1.042569795 | 3.578456245 | 10.44036547 | 2.95E-23 | 4.08E-22 | 41.92886405 |
| PTTG1 | 1.370495046 | 6.459645321 | 10.4397422 | 2.97E-23 | 4.09E-22 | 41.92351934 |
| PROC | 1.132217223 | 2.206945832 | 10.43599536 | 3.06E-23 | 4.23E-22 | 41.89139383 |
| MYO1F | 1.090291341 | 8.555613791 | 10.42576084 | 3.35E-23 | 4.61E-22 | 41.80368067 |
| CHGA | -2.256220047 | 9.265572667 | -10.42569873 | 3.35E-23 | 4.61E-22 | 41.80314852 |
| COL1A1 | 2.222201501 | 7.79968844 | 10.41878227 | 3.56E-23 | 4.88E-22 | 41.74390396 |
| KLHDC7A | 1.381448663 | 0.974350248 | 10.41010213 | 3.83E-23 | 5.25E-22 | 41.66958826 |
| CACNA2D3 | -1.819992509 | 6.253858704 | -10.40909481 | 3.87E-23 | 5.29E-22 | 41.66096658 |
| ALOX12B | -1.553941604 | 2.775404256 | -10.40101218 | 4.15E-23 | 5.66E-22 | 41.59180686 |
| RBM47 | 1.230851797 | 6.649980122 | 10.3955843 | 4.35E-23 | 5.92E-22 | 41.54538229 |
| C1orf88 | 1.370991474 | 7.428966266 | 10.37964646 | 4.99E-23 | 6.76E-22 | 41.40915719 |
| CPVL | 1.240278135 | 8.430627849 | 10.37873296 | 5.03E-23 | 6.81E-22 | 41.4013533 |
| CLEC4F | -1.791865881 | 4.313431226 | -10.37789705 | 5.07E-23 | 6.85E-22 | 41.39421275 |
| CXCR3 | 1.267844792 | 1.427382738 | 10.37074846 | 5.39E-23 | 7.28E-22 | 41.33316252 |
| FGF12 | -1.126903969 | 9.003070211 | -10.36921935 | 5.46E-23 | 7.36E-22 | 41.32010716 |
| RPS6KA6 | -1.56603233 | 4.773566025 | -10.36391088 | 5.72E-23 | 7.68E-22 | 41.27479401 |
| GJD3 | 1.346530434 | 3.525116112 | 10.36181667 | 5.82E-23 | 7.82E-22 | 41.25692193 |
| APOC2 | 1.451265012 | 8.553655173 | 10.36004388 | 5.91E-23 | 7.93E-22 | 41.24179465 |
| ETV7 | 1.319914214 | 3.103639267 | 10.35882066 | 5.98E-23 | 8.01E-22 | 41.23135794 |
| CD248 | 1.356225369 | 6.410338618 | 10.35504004 | 6.17E-23 | 8.27E-22 | 41.19910595 |
| RDH5 | 1.051749675 | 5.767423099 | 10.3515957 | 6.36E-23 | 8.51E-22 | 41.16972946 |
| CTSC | 1.061864103 | 9.037189643 | 10.35026088 | 6.43E-23 | 8.60E-22 | 41.15834654 |
| HSPA12A | -1.064655175 | 9.808492713 | -10.33395262 | 7.41E-23 | 9.88E-22 | 41.01935274 |
| SCN3B | -1.683613921 | 10.1682992 | -10.33271745 | 7.49E-23 | 9.98E-22 | 41.00883131 |
| PDIA5 | 1.085211392 | 6.012856255 | 10.32658407 | 7.89E-23 | 1.05E-21 | 40.95659813 |
| CCNA2 | 1.317802274 | 6.226785701 | 10.32149836 | 8.25E-23 | 1.10E-21 | 40.91330245 |
| ULBP1 | 1.376009048 | 2.170685191 | 10.31924876 | 8.41E-23 | 1.12E-21 | 40.89415565 |
| DDIT4L | 2.258427675 | 4.526922662 | 10.31825535 | 8.48E-23 | 1.12E-21 | 40.8857014 |
| MYCBPAP | 1.25891983 | 2.906774358 | 10.3174812 | 8.54E-23 | 1.13E-21 | 40.87911341 |
| HCP5 | 1.078327489 | 6.790534592 | 10.29113716 | 1.07E-22 | 1.41E-21 | 40.6551215 |
| ITGAL | 1.374233807 | 6.843881842 | 10.28994896 | 1.08E-22 | 1.42E-21 | 40.64502755 |
| OTOS | 1.861852367 | 2.160663806 | 10.28458429 | 1.13E-22 | 1.48E-21 | 40.59946344 |
| C2orf66 | 1.086085799 | 1.824865499 | 10.2818813 | 1.16E-22 | 1.52E-21 | 40.57651188 |
| SLC47A2 | 2.377013819 | 4.552696422 | 10.28104047 | 1.17E-22 | 1.53E-21 | 40.56937307 |
| VAX2 | -1.380735925 | 7.089996378 | -10.28102326 | 1.17E-22 | 1.53E-21 | 40.56922696 |
| TOX | -1.143446581 | 9.489284589 | -10.27924402 | 1.19E-22 | 1.55E-21 | 40.5541221 |
| HBQ1 | -1.351499527 | 2.750964331 | -10.27877311 | 1.19E-22 | 1.55E-21 | 40.55012457 |
| PRAM1 | 1.296321346 | 6.364260195 | 10.27528073 | 1.23E-22 | 1.60E-21 | 40.52048185 |
| ASB5 | 1.572446573 | 2.629030449 | 10.27205483 | 1.26E-22 | 1.64E-21 | 40.49310681 |
| LOC153328 | -1.899575949 | 7.290674969 | -10.27203797 | 1.26E-22 | 1.64E-21 | 40.49296373 |
| PCSK2 | -2.130800818 | 8.420572305 | -10.25379967 | 1.48E-22 | 1.91E-21 | 40.3382996 |
| CDC6 | 1.203591382 | 5.733622954 | 10.25353726 | 1.48E-22 | 1.91E-21 | 40.3360756 |
| FGL2 | 1.223888492 | 7.871365886 | 10.25031947 | 1.52E-22 | 1.97E-21 | 40.30880735 |
| EMR1 | 1.943556966 | 2.822112351 | 10.24687621 | 1.57E-22 | 2.02E-21 | 40.27963462 |
| C17orf102 | -1.429325819 | 4.148536025 | -10.24632362 | 1.57E-22 | 2.03E-21 | 40.27495341 |
| GPR179 | -1.335230388 | 5.779910573 | -10.23852194 | 1.68E-22 | 2.16E-21 | 40.20888051 |
| KIF20A | 2.033753637 | 4.953400003 | 10.23818605 | 1.69E-22 | 2.17E-21 | 40.20603654 |
| ACCS | 1.2587878 | 5.78459524 | 10.23788783 | 1.69E-22 | 2.17E-21 | 40.20351161 |
| FNDC7 | 1.304007347 | 2.216705234 | 10.23613811 | 1.72E-22 | 2.20E-21 | 40.18869825 |
| NECAB2 | -1.326638201 | 8.213390161 | -10.23310183 | 1.76E-22 | 2.26E-21 | 40.16299675 |
| ERP27 | 1.240090958 | 2.528862819 | 10.22855738 | 1.83E-22 | 2.34E-21 | 40.12453812 |
| C20orf26 | 1.366371348 | 4.392961921 | 10.22169988 | 1.94E-22 | 2.48E-21 | 40.066526 |
| SUSD5 | -1.961117159 | 8.595673336 | -10.21872881 | 1.99E-22 | 2.54E-21 | 40.0413997 |
| DKFZp779M0652 | -1.176967333 | 4.591236771 | -10.21594867 | 2.04E-22 | 2.59E-21 | 40.01789252 |
| LRRC36 | 1.253386748 | 2.874130239 | 10.21516312 | 2.06E-22 | 2.61E-21 | 40.0112511 |
| PDLIM1 | 1.389911921 | 6.494650671 | 10.1986112 | 2.37E-22 | 2.99E-21 | 39.87139213 |
| CORO6 | -1.644299156 | 6.159158274 | -10.19845855 | 2.37E-22 | 2.99E-21 | 39.87010299 |
| HOXC5 | 1.519978295 | 1.118188438 | 10.18998796 | 2.55E-22 | 3.21E-21 | 39.79858748 |
| LPPR1 | -1.40185404 | 10.39332305 | -10.18218651 | 2.72E-22 | 3.42E-21 | 39.73275605 |
| PDGFD | 1.847489399 | 5.361481559 | 10.18037414 | 2.77E-22 | 3.47E-21 | 39.71746744 |
| SAA2 | 1.244830996 | 0.70814193 | 10.17686008 | 2.85E-22 | 3.57E-21 | 39.68782893 |
| HERC5 | 1.263227058 | 7.398194111 | 10.17611095 | 2.87E-22 | 3.59E-21 | 39.68151141 |
| FEZF1 | 1.467449214 | 0.900988936 | 10.16692285 | 3.10E-22 | 3.86E-21 | 39.60405228 |
| PCDH20 | -1.657899115 | 7.175333398 | -10.16662926 | 3.11E-22 | 3.87E-21 | 39.60157802 |
| ITK | 1.61302696 | 2.678378549 | 10.1660956 | 3.13E-22 | 3.88E-21 | 39.59708056 |
| GRM4 | -1.750294163 | 4.603980916 | -10.16334263 | 3.20E-22 | 3.97E-21 | 39.57388225 |
| HTR2C | -1.828524615 | 4.204304637 | -10.16153294 | 3.25E-22 | 4.03E-21 | 39.55863486 |
| HSPB1 | 1.125506518 | 10.8584633 | 10.1572752 | 3.37E-22 | 4.17E-21 | 39.52276883 |
| MYH7B | -1.225145027 | 6.567848761 | -10.1534376 | 3.48E-22 | 4.30E-21 | 39.49045042 |
| TRIM67 | -2.619530621 | 8.081787341 | -10.15169325 | 3.53E-22 | 4.36E-21 | 39.47576304 |
| MYOD1 | -2.084006713 | 2.361627531 | -10.14922677 | 3.61E-22 | 4.45E-21 | 39.45499816 |
| TRIM6 | 1.089120604 | 4.417460823 | 10.14586532 | 3.71E-22 | 4.58E-21 | 39.42670411 |
| TTC9B | -1.515944104 | 7.034441917 | -10.14212635 | 3.83E-22 | 4.72E-21 | 39.39523962 |
| TGM5 | 1.435340256 | 1.88198544 | 10.14199193 | 3.84E-22 | 4.72E-21 | 39.39410856 |
| PDZD7 | -1.205245588 | 5.210448041 | -10.14062929 | 3.88E-22 | 4.77E-21 | 39.3826436 |
| CD69 | 1.61217336 | 4.360225424 | 10.14054191 | 3.89E-22 | 4.77E-21 | 39.38190848 |
| CD3D | 1.489315341 | 1.646643986 | 10.13997569 | 3.91E-22 | 4.78E-21 | 39.37714469 |
| RPL21 | -1.48760622 | 3.501520814 | -10.12957151 | 4.27E-22 | 5.21E-21 | 39.28964347 |
| ESPNL | 1.29623167 | 2.115506307 | 10.12951665 | 4.27E-22 | 5.21E-21 | 39.28918225 |
| IL1RAPL1 | -1.272844513 | 4.539283243 | -10.12073239 | 4.60E-22 | 5.61E-21 | 39.21535153 |
| CCDC89 | 1.15653811 | 4.497608214 | 10.11937667 | 4.65E-22 | 5.67E-21 | 39.20396063 |
| FGFRL1 | 1.108171229 | 8.593286226 | 10.11510014 | 4.83E-22 | 5.87E-21 | 39.16803545 |
| SPATA6 | 1.469140204 | 6.512539917 | 10.11113267 | 4.99E-22 | 6.05E-21 | 39.1347156 |
| RAD54L | 1.331266143 | 5.142365963 | 10.10459555 | 5.28E-22 | 6.39E-21 | 39.0798341 |
| FAM196B | -1.675086878 | 6.280668227 | -10.10399796 | 5.30E-22 | 6.42E-21 | 39.07481825 |
| SYP | -1.129481699 | 11.41627392 | -10.09664883 | 5.65E-22 | 6.83E-21 | 39.01315023 |
| KCNN1 | -1.136761684 | 7.921128755 | -10.09503834 | 5.72E-22 | 6.91E-21 | 38.99964025 |
| WSCD2 | -2.123098005 | 6.578525779 | -10.09210716 | 5.87E-22 | 7.08E-21 | 38.97505503 |
| DNAJC22 | 1.077321144 | 4.09017705 | 10.09201853 | 5.87E-22 | 7.08E-21 | 38.97431177 |
| IGFALS | -1.053361071 | 4.068760872 | -10.09135197 | 5.91E-22 | 7.12E-21 | 38.96872166 |
| PADI1 | 1.00269893 | 1.000615495 | 10.08684543 | 6.14E-22 | 7.39E-21 | 38.93093437 |
| KRT222 | -1.034592556 | 6.307082555 | -10.08299928 | 6.34E-22 | 7.61E-21 | 38.89869323 |
| JAKMIP3 | -1.095846661 | 7.233554478 | -10.06836026 | 7.18E-22 | 8.57E-21 | 38.77605381 |
| CLEC12A | 1.48500864 | 1.796851149 | 10.06745006 | 7.23E-22 | 8.63E-21 | 38.76843247 |
| ITPRIPL1 | 1.336881868 | 4.946621324 | 10.06705145 | 7.26E-22 | 8.65E-21 | 38.76509499 |
| CD3E | 1.650916937 | 3.243618364 | 10.05972661 | 7.72E-22 | 9.19E-21 | 38.7037805 |
| TACR1 | -1.807055552 | 6.646915895 | -10.05021958 | 8.37E-22 | 9.91E-21 | 38.62424392 |
| DSG2 | 2.009928417 | 2.285212631 | 10.04762222 | 8.56E-22 | 1.01E-20 | 38.60252297 |
| GALNT14 | -1.456376555 | 5.990454907 | -10.04576052 | 8.69E-22 | 1.03E-20 | 38.58695639 |
| NXPH1 | -1.457606525 | 9.583272431 | -10.04287797 | 8.91E-22 | 1.05E-20 | 38.56285774 |
| SEMA4A | -1.04728099 | 8.214290683 | -10.03983363 | 9.14E-22 | 1.08E-20 | 38.53741163 |
| GABRG2 | -2.503147438 | 7.95718469 | -10.03927996 | 9.18E-22 | 1.08E-20 | 38.53278426 |
| NCF1 | 1.289235314 | 5.589282921 | 10.03864172 | 9.23E-22 | 1.09E-20 | 38.52745043 |
| PVT1 | 1.267181416 | 3.706617261 | 10.03344802 | 9.65E-22 | 1.13E-20 | 38.48405414 |
| NTN4 | -1.333258821 | 8.650373006 | -10.03287682 | 9.70E-22 | 1.14E-20 | 38.47928237 |
| UNC80 | -1.108413074 | 9.976068398 | -10.0321522 | 9.76E-22 | 1.15E-20 | 38.47322918 |
| DLGAP5 | 2.091328136 | 4.165346886 | 10.02716936 | 1.02E-21 | 1.19E-20 | 38.4316125 |
| MYCNOS | -1.040769536 | 3.29985689 | -10.02584209 | 1.03E-21 | 1.20E-20 | 38.42052947 |
| TREM1 | 1.72946381 | 2.556426294 | 10.02306704 | 1.05E-21 | 1.23E-20 | 38.39736028 |
| CNNM1 | -1.775549687 | 6.588342395 | -10.02131554 | 1.07E-21 | 1.24E-20 | 38.38273899 |
| TMEM176B | 1.254115464 | 9.098439059 | 10.01364333 | 1.14E-21 | 1.33E-20 | 38.31871279 |
| RERGL | -1.028942572 | 5.969142988 | -10.01176864 | 1.16E-21 | 1.35E-20 | 38.3030731 |
| ZAP70 | 1.165218542 | 3.529937424 | 10.00979052 | 1.18E-21 | 1.37E-20 | 38.28657264 |
| COL3A1 | 2.122292274 | 7.681956144 | 9.995944312 | 1.32E-21 | 1.53E-20 | 38.17113602 |
| KMO | 1.357489793 | 2.919912457 | 9.994034451 | 1.35E-21 | 1.55E-20 | 38.15522182 |
| HGF | 1.433158145 | 4.653684548 | 9.993547917 | 1.35E-21 | 1.56E-20 | 38.15116802 |
| IL2RB | 1.496365415 | 3.577477282 | 9.993284242 | 1.35E-21 | 1.56E-20 | 38.14897115 |
| IRX5 | 1.905373419 | 2.726564807 | 9.990124372 | 1.39E-21 | 1.60E-20 | 38.12264684 |
| MAN1C1 | 1.185657804 | 9.055517937 | 9.989175073 | 1.40E-21 | 1.61E-20 | 38.1147395 |
| GPR21 | -1.293346881 | 2.762845506 | -9.981489425 | 1.50E-21 | 1.72E-20 | 38.0507392 |
| ECHDC2 | 1.093286842 | 7.005166217 | 9.978112804 | 1.54E-21 | 1.76E-20 | 38.02263169 |
| DLGAP1 | -1.265108433 | 9.230802993 | -9.969486597 | 1.66E-21 | 1.89E-20 | 37.95085485 |
| LOXL1 | 1.573765527 | 4.707709363 | 9.962711309 | 1.75E-21 | 2.00E-20 | 37.89450838 |
| UBE2QL1 | -1.306467281 | 8.60440599 | -9.955434138 | 1.86E-21 | 2.12E-20 | 37.83401672 |
| C21orf62 | 1.761782379 | 7.568554445 | 9.952839562 | 1.91E-21 | 2.17E-20 | 37.81245644 |
| LPIN3 | 1.091030807 | 4.585442876 | 9.949838328 | 1.95E-21 | 2.22E-20 | 37.78752165 |
| CHRM1 | -1.946370734 | 7.473732066 | -9.943967453 | 2.05E-21 | 2.33E-20 | 37.73876 |
| PIPOX | 1.161401314 | 8.469725938 | 9.942243011 | 2.08E-21 | 2.37E-20 | 37.72444101 |
| APOB48R | 1.023087625 | 6.87003334 | 9.941791421 | 2.09E-21 | 2.37E-20 | 37.72069148 |
| KIF14 | 1.593276179 | 4.975576041 | 9.938490219 | 2.15E-21 | 2.44E-20 | 37.6932853 |
| IFI6 | 1.246221582 | 10.36560793 | 9.932218553 | 2.27E-21 | 2.56E-20 | 37.64123557 |
| CHRNB2 | -1.346803736 | 8.823899467 | -9.923827476 | 2.43E-21 | 2.74E-20 | 37.5716311 |
| FAM70A | 1.013685411 | 9.463799537 | 9.922800834 | 2.45E-21 | 2.76E-20 | 37.56311778 |
| TWIST1 | 1.180679772 | 4.355990871 | 9.91814145 | 2.55E-21 | 2.86E-20 | 37.52448776 |
| IBSP | 2.015528231 | 1.484464306 | 9.918072027 | 2.55E-21 | 2.86E-20 | 37.52391228 |
| SHROOM3 | 1.562615711 | 6.58215273 | 9.917187127 | 2.57E-21 | 2.88E-20 | 37.51657717 |
| KDELR3 | 1.018847386 | 4.27502008 | 9.915351697 | 2.61E-21 | 2.93E-20 | 37.50136434 |
| DCX | -1.589669269 | 10.17538862 | -9.912077799 | 2.68E-21 | 3.00E-20 | 37.47423359 |
| PNMA3 | -1.316191521 | 8.097346964 | -9.908998509 | 2.75E-21 | 3.07E-20 | 37.44872108 |
| KIAA2022 | -1.151938537 | 7.913749253 | -9.908459943 | 2.77E-21 | 3.08E-20 | 37.4442595 |
| SERPINE1 | 1.956147848 | 7.810304545 | 9.907785873 | 2.78E-21 | 3.10E-20 | 37.43867562 |
| SGCD | -1.117399491 | 8.39567944 | -9.906682967 | 2.81E-21 | 3.13E-20 | 37.42953989 |
| BTBD17 | -1.418554285 | 8.882668337 | -9.89931154 | 2.99E-21 | 3.32E-20 | 37.3684976 |
| FHAD1 | 1.259715716 | 3.472381123 | 9.892677677 | 3.16E-21 | 3.50E-20 | 37.31358929 |
| PTCHD2 | -1.144886284 | 9.389330033 | -9.887604306 | 3.30E-21 | 3.65E-20 | 37.27161393 |
| GPR123 | -1.00221162 | 9.712166072 | -9.887556872 | 3.30E-21 | 3.65E-20 | 37.27122155 |
| GATA5 | -1.567665326 | 1.751918924 | -9.887444431 | 3.30E-21 | 3.65E-20 | 37.27029142 |
| ZSCAN1 | -1.136246767 | 5.662819602 | -9.884518826 | 3.38E-21 | 3.74E-20 | 37.24609285 |
| CLGN | -1.354523451 | 7.059731526 | -9.87671105 | 3.61E-21 | 3.98E-20 | 37.18153607 |
| KLHDC8A | 1.252231073 | 8.907671168 | 9.872155259 | 3.75E-21 | 4.13E-20 | 37.14388353 |
| NEXN | 1.069117345 | 6.174772424 | 9.869768533 | 3.83E-21 | 4.20E-20 | 37.12416251 |
| HDC | 1.295293512 | 0.956878612 | 9.863237543 | 4.04E-21 | 4.44E-20 | 37.07021481 |
| SGMS2 | 1.001909617 | 4.872314989 | 9.861674713 | 4.09E-21 | 4.49E-20 | 37.05730901 |
| FAM190A | -1.394766969 | 3.497954639 | -9.858110169 | 4.22E-21 | 4.62E-20 | 37.02787831 |
| CXCL11 | 1.687326078 | 2.801519321 | 9.857256317 | 4.25E-21 | 4.65E-20 | 37.02082955 |
| GPR55 | 1.04269685 | 2.979862905 | 9.853186964 | 4.40E-21 | 4.81E-20 | 36.98724169 |
| MMD2 | -1.53095801 | 8.832469513 | -9.850323516 | 4.50E-21 | 4.93E-20 | 36.96361284 |
| PRKG2 | -1.991340742 | 4.371972256 | -9.847885558 | 4.59E-21 | 5.02E-20 | 36.94349876 |
| TCTE1 | -1.043672204 | 3.588591807 | -9.842640852 | 4.80E-21 | 5.24E-20 | 36.9002394 |
| NT5C1A | -1.492293571 | 3.495035638 | -9.837532472 | 5.01E-21 | 5.46E-20 | 36.85811953 |
| CXorf57 | -1.214975333 | 8.032003485 | -9.833879497 | 5.16E-21 | 5.63E-20 | 36.82800896 |
| GZMK | 1.543518997 | 1.938833993 | 9.83234967 | 5.23E-21 | 5.69E-20 | 36.81540122 |
| UNC13A | -1.192388081 | 10.6537621 | -9.83166358 | 5.26E-21 | 5.72E-20 | 36.8097474 |
| APCDD1L | 1.640141219 | 1.131685609 | 9.828160671 | 5.42E-21 | 5.88E-20 | 36.78088534 |
| C21orf125 | -1.383706972 | 3.294227295 | -9.825159369 | 5.55E-21 | 6.02E-20 | 36.75616182 |
| ALOX5 | 1.177616869 | 7.462117341 | 9.82432376 | 5.59E-21 | 6.06E-20 | 36.74927931 |
| ADAMTS19 | -1.529949161 | 4.022553074 | -9.821501153 | 5.73E-21 | 6.20E-20 | 36.72603384 |
| OAS3 | 1.143659669 | 9.058372241 | 9.819304687 | 5.83E-21 | 6.31E-20 | 36.70794806 |
| KCNQ2 | -1.121626349 | 10.2365714 | -9.807689077 | 6.43E-21 | 6.92E-20 | 36.61235055 |
| MX2 | 1.31598378 | 5.950901025 | 9.800773927 | 6.81E-21 | 7.32E-20 | 36.55547484 |
| F7 | -1.394074105 | 3.584238135 | -9.799408771 | 6.89E-21 | 7.40E-20 | 36.54424994 |
| HOXB13 | 1.487897546 | 0.897133817 | 9.797292107 | 7.01E-21 | 7.52E-20 | 36.52684792 |
| PCDHB1 | 1.010757537 | 1.52100732 | 9.796182223 | 7.07E-21 | 7.59E-20 | 36.5177241 |
| RHBDL1 | -1.156961386 | 6.604494941 | -9.787921659 | 7.58E-21 | 8.11E-20 | 36.44984018 |
| ORC1L | 1.387319241 | 4.395579091 | 9.786429231 | 7.67E-21 | 8.20E-20 | 36.43757983 |
| HIST3H2A | -1.194764717 | 6.24763858 | -9.778561135 | 8.19E-21 | 8.73E-20 | 36.3729642 |
| HOXB7 | 1.630129189 | 3.089555125 | 9.773442622 | 8.55E-21 | 9.10E-20 | 36.33094819 |
| PODNL1 | 1.326933058 | 3.873861166 | 9.767192159 | 9.00E-21 | 9.57E-20 | 36.27966078 |
| GALNT9 | -1.803332324 | 8.029084724 | -9.737474072 | 1.15E-20 | 1.22E-19 | 36.0361197 |
| COL8A2 | 1.313524442 | 7.025892132 | 9.737397601 | 1.15E-20 | 1.22E-19 | 36.03549368 |
| FANCI | 1.078313606 | 7.908898677 | 9.726921781 | 1.26E-20 | 1.33E-19 | 35.94976547 |
| MMP11 | 1.146426127 | 4.201030402 | 9.724412539 | 1.28E-20 | 1.35E-19 | 35.92924063 |
| ANG | 1.125352898 | 4.843706739 | 9.710781836 | 1.44E-20 | 1.51E-19 | 35.8178091 |
| ADAMTS7 | 1.258493123 | 5.292916147 | 9.704872461 | 1.51E-20 | 1.58E-19 | 35.76953308 |
| KIAA1486 | -1.74651687 | 3.334317686 | -9.700777654 | 1.56E-20 | 1.63E-19 | 35.7360928 |
| IDO1 | 1.5369343 | 1.167485725 | 9.699073613 | 1.58E-20 | 1.65E-19 | 35.7221796 |
| HSF4 | -1.258485869 | 7.134131264 | -9.69776634 | 1.60E-20 | 1.67E-19 | 35.71150708 |
| LIF | 1.722394864 | 4.463753992 | 9.692409169 | 1.67E-20 | 1.74E-19 | 35.66778167 |
| C9orf4 | -1.659299409 | 5.007268987 | -9.690192461 | 1.70E-20 | 1.77E-19 | 35.6496937 |
| IQGAP3 | 1.62602032 | 5.736915967 | 9.688443717 | 1.73E-20 | 1.80E-19 | 35.63542623 |
| LUZP2 | -1.515837585 | 10.74040287 | -9.686258871 | 1.76E-20 | 1.83E-19 | 35.61760323 |
| CDK5R2 | -1.687277029 | 8.094067135 | -9.685592812 | 1.77E-20 | 1.84E-19 | 35.61217037 |
| KCTD4 | -1.282295075 | 6.520862777 | -9.678691589 | 1.87E-20 | 1.94E-19 | 35.55589409 |
| PIK3R6 | 1.044572119 | 4.669363304 | 9.677087295 | 1.90E-20 | 1.96E-19 | 35.54281578 |
| PKIB | 1.415311071 | 5.452969615 | 9.674378443 | 1.94E-20 | 2.00E-19 | 35.52073641 |
| GBP3 | 1.559730678 | 7.926998936 | 9.672788055 | 1.97E-20 | 2.03E-19 | 35.50777543 |
| RAB38 | 1.008810571 | 2.697248761 | 9.664524161 | 2.11E-20 | 2.17E-19 | 35.44045183 |
| ITGB2 | 1.181068853 | 10.23474188 | 9.660959013 | 2.17E-20 | 2.23E-19 | 35.41141982 |
| RAD51AP1 | 1.091463362 | 5.772610548 | 9.659945434 | 2.19E-20 | 2.25E-19 | 35.40316732 |
| CHST6 | 1.32643016 | 7.992974239 | 9.651705484 | 2.34E-20 | 2.40E-19 | 35.33610018 |
| CA10 | -1.765518806 | 9.039049721 | -9.648752777 | 2.40E-20 | 2.46E-19 | 35.31207692 |
| TPX2 | 1.417841548 | 7.887977836 | 9.648591511 | 2.40E-20 | 2.46E-19 | 35.310765 |
| CRLF1 | -2.356066361 | 7.951816698 | -9.647711161 | 2.42E-20 | 2.47E-19 | 35.30360353 |
| TLR2 | 1.337394875 | 7.717404812 | 9.644025427 | 2.50E-20 | 2.55E-19 | 35.27362571 |
| FHDC1 | -1.383769811 | 8.08581595 | -9.643080713 | 2.51E-20 | 2.57E-19 | 35.26594317 |
| KCNJ12 | -1.340054434 | 5.348620705 | -9.64172756 | 2.54E-20 | 2.59E-19 | 35.25494006 |
| KYNU | 1.333815839 | 3.948595106 | 9.640694671 | 2.56E-20 | 2.61E-19 | 35.24654189 |
| MEX3B | -1.049070249 | 7.522694957 | -9.639403437 | 2.59E-20 | 2.64E-19 | 35.23604403 |
| ANK1 | -1.340617731 | 7.796054511 | -9.63746728 | 2.63E-20 | 2.68E-19 | 35.22030472 |
| FGF14 | -1.065639204 | 8.244121705 | -9.636911941 | 2.65E-20 | 2.69E-19 | 35.2157907 |
| THSD4 | -1.182161672 | 8.178250789 | -9.636506056 | 2.65E-20 | 2.70E-19 | 35.2124916 |
| PTPRC | 1.203485922 | 8.169296844 | 9.634284801 | 2.70E-20 | 2.74E-19 | 35.19443863 |
| MICB | 1.006697165 | 4.8640112 | 9.625199608 | 2.91E-20 | 2.95E-19 | 35.12062975 |
| SRRM4 | -1.862370765 | 7.363191486 | -9.622296652 | 2.98E-20 | 3.02E-19 | 35.09705603 |
| G6PC2 | -1.847740982 | 2.552602626 | -9.62028396 | 3.03E-20 | 3.07E-19 | 35.08071467 |
| TTC12 | 1.362149075 | 4.781890155 | 9.618436038 | 3.08E-20 | 3.11E-19 | 35.06571318 |
| GNLY | 1.615095035 | 3.54398702 | 9.611898831 | 3.25E-20 | 3.28E-19 | 35.01265993 |
| LOC100270710 | 1.026749216 | 4.849064144 | 9.607402428 | 3.37E-20 | 3.39E-19 | 34.97618348 |
| PRPS2 | 1.047355646 | 7.225498104 | 9.606406654 | 3.40E-20 | 3.42E-19 | 34.968107 |
| ARHGAP11A | 1.129458859 | 6.729509814 | 9.604896474 | 3.44E-20 | 3.46E-19 | 34.9558594 |
| LRTM2 | -2.045730961 | 6.205381564 | -9.594402834 | 3.75E-20 | 3.76E-19 | 34.87079253 |
| EMID2 | -1.535683789 | 6.380926707 | -9.594225568 | 3.76E-20 | 3.77E-19 | 34.86935607 |
| CPNE5 | -1.242419983 | 10.05579993 | -9.592532734 | 3.81E-20 | 3.82E-19 | 34.8556393 |
| GRIN1 | -2.38422969 | 9.346847649 | -9.590536379 | 3.87E-20 | 3.88E-19 | 34.83946529 |
| MXRA5 | 1.50048558 | 6.525458796 | 9.589369927 | 3.91E-20 | 3.91E-19 | 34.83001604 |
| PARVG | 1.066135668 | 7.866331516 | 9.588586672 | 3.94E-20 | 3.94E-19 | 34.82367146 |
| CRB2 | 1.460304546 | 8.515091735 | 9.587553995 | 3.97E-20 | 3.97E-19 | 34.81530704 |
| HSPB6 | 1.515985968 | 8.602006962 | 9.584049576 | 4.09E-20 | 4.07E-19 | 34.78692681 |
| MFAP2 | 1.778523587 | 3.534147546 | 9.582325648 | 4.14E-20 | 4.13E-19 | 34.77296837 |
| ARHGAP15 | 1.256279927 | 4.947840861 | 9.563481173 | 4.84E-20 | 4.79E-19 | 34.62050049 |
| C11orf82 | 1.060702747 | 5.363647655 | 9.55954056 | 5.00E-20 | 4.93E-19 | 34.58864392 |
| FAM83D | 1.252966922 | 5.035200518 | 9.55915986 | 5.01E-20 | 4.95E-19 | 34.58556675 |
| AQP1 | 1.911466573 | 12.12676143 | 9.556799223 | 5.11E-20 | 5.04E-19 | 34.56648786 |
| UPP2 | -1.041726442 | 4.021885648 | -9.549889734 | 5.41E-20 | 5.32E-19 | 34.51066355 |
| RAC2 | 1.102055892 | 6.430101409 | 9.547484657 | 5.51E-20 | 5.42E-19 | 34.49123862 |
| C1orf87 | 1.546462409 | 1.669282655 | 9.546945936 | 5.54E-20 | 5.44E-19 | 34.48688803 |
| SLC16A3 | 1.010725488 | 7.898890532 | 9.546582616 | 5.56E-20 | 5.45E-19 | 34.48395404 |
| GRB14 | 1.723366981 | 3.162495128 | 9.543410192 | 5.70E-20 | 5.59E-19 | 34.45833843 |
| SLAMF6 | 1.211334167 | 2.43213785 | 9.539689049 | 5.88E-20 | 5.75E-19 | 34.42829975 |
| PNMA5 | -1.669578995 | 4.75654903 | -9.539666449 | 5.88E-20 | 5.75E-19 | 34.42811734 |
| LOC285954 | -1.611138883 | 3.792266965 | -9.537589838 | 5.98E-20 | 5.85E-19 | 34.41135761 |
| CCDC65 | 1.165228225 | 3.948472552 | 9.525727953 | 6.59E-20 | 6.43E-19 | 34.31567242 |
| CLSPN | 1.521103425 | 4.460509596 | 9.512921971 | 7.32E-20 | 7.11E-19 | 34.21246468 |
| GZMA | 1.535492149 | 2.358329781 | 9.510684301 | 7.45E-20 | 7.24E-19 | 34.19444048 |
| TC2N | 1.550535046 | 5.242071776 | 9.508163297 | 7.61E-20 | 7.38E-19 | 34.17413759 |
| CALCRL | -1.296208507 | 10.73386594 | -9.501208202 | 8.05E-20 | 7.78E-19 | 34.11814424 |
| TAS1R1 | 1.25170113 | 3.011271942 | 9.499380768 | 8.17E-20 | 7.90E-19 | 34.10343687 |
| FBP1 | 1.116478575 | 5.650343784 | 9.489088882 | 8.89E-20 | 8.57E-19 | 34.02064364 |
| MADCAM1 | -1.544916771 | 3.693576916 | -9.482842285 | 9.35E-20 | 9.01E-19 | 33.97042336 |
| FGFBP3 | -1.093091061 | 8.696833097 | -9.474726517 | 9.99E-20 | 9.61E-19 | 33.90521018 |
| FGF9 | -1.41508208 | 5.765680034 | -9.469422423 | 1.04E-19 | 9.99E-19 | 33.86261094 |
| SLC7A7 | 1.006185112 | 7.712546448 | 9.46875973 | 1.05E-19 | 1.00E-18 | 33.85728976 |
| MFAP4 | 1.20128367 | 8.122597202 | 9.456442985 | 1.16E-19 | 1.11E-18 | 33.7584384 |
| FAM163B | -1.908945628 | 5.502182338 | -9.455400135 | 1.17E-19 | 1.12E-18 | 33.75007287 |
| ITGA2 | 1.193259235 | 7.206696451 | 9.452635144 | 1.20E-19 | 1.14E-18 | 33.72789579 |
| LTBP1 | 1.055534953 | 9.194536057 | 9.442633365 | 1.30E-19 | 1.23E-18 | 33.64771275 |
| IL2RG | 1.188473482 | 4.759231679 | 9.442366928 | 1.30E-19 | 1.23E-18 | 33.64557757 |
| MUC6 | -1.078214994 | 3.109922968 | -9.441507677 | 1.31E-19 | 1.24E-18 | 33.63869197 |
| APOL1 | 1.019002794 | 7.516605872 | 9.440728076 | 1.32E-19 | 1.25E-18 | 33.63244502 |
| IL15 | 1.142183152 | 2.918929349 | 9.423692398 | 1.51E-19 | 1.43E-18 | 33.49602846 |
| TCHH | -1.093108939 | 3.999130255 | -9.416686149 | 1.60E-19 | 1.51E-18 | 33.43997471 |
| KCNK12 | -1.399367985 | 4.703611633 | -9.414041774 | 1.64E-19 | 1.54E-18 | 33.41882591 |
| SASH3 | 1.069497303 | 7.906436552 | 9.412689231 | 1.65E-19 | 1.55E-18 | 33.40801035 |
| CD5 | 1.087272929 | 2.656851382 | 9.409319044 | 1.70E-19 | 1.59E-18 | 33.38106552 |
| FBXL16 | -1.178766482 | 11.16596623 | -9.405742875 | 1.75E-19 | 1.64E-18 | 33.35248127 |
| ANGPT1 | 1.395672421 | 6.285248041 | 9.403287426 | 1.78E-19 | 1.67E-18 | 33.33285933 |
| BRCA2 | 1.218255772 | 4.869475189 | 9.387161024 | 2.03E-19 | 1.89E-18 | 33.20407992 |
| C6orf147 | 1.021763106 | 3.120358731 | 9.380586183 | 2.14E-19 | 2.00E-18 | 33.15162035 |
| NDC80 | 1.762847148 | 5.193959605 | 9.37616627 | 2.22E-19 | 2.06E-18 | 33.11636914 |
| SEL1L3 | 1.568813322 | 7.436276105 | 9.367412041 | 2.39E-19 | 2.21E-18 | 33.04658397 |
| CTNNA3 | -1.330094093 | 5.512441004 | -9.358600981 | 2.56E-19 | 2.37E-18 | 32.97639218 |
| TCEA3 | 1.305391614 | 5.183685641 | 9.350927719 | 2.73E-19 | 2.52E-18 | 32.91530243 |
| FAM70B | 1.019127317 | 4.287495663 | 9.350148533 | 2.74E-19 | 2.53E-18 | 32.90910101 |
| CNTN4 | -1.443134186 | 6.09059433 | -9.348103258 | 2.79E-19 | 2.57E-18 | 32.89282473 |
| KIF26A | -1.30494051 | 7.939617069 | -9.347225794 | 2.81E-19 | 2.59E-18 | 32.88584264 |
| SCNN1B | 1.535754453 | 1.568061018 | 9.343213112 | 2.90E-19 | 2.67E-18 | 32.85391918 |
| ABI3 | 1.036028005 | 7.777922055 | 9.333403567 | 3.14E-19 | 2.89E-18 | 32.7759187 |
| DKFZp434J0226 | -1.868354664 | 3.925249147 | -9.332676784 | 3.16E-19 | 2.90E-18 | 32.77014199 |
| DGKI | -1.05318015 | 6.884827662 | -9.3320663 | 3.17E-19 | 2.92E-18 | 32.76528992 |
| L1CAM | -2.182800992 | 9.464423193 | -9.329912385 | 3.23E-19 | 2.96E-18 | 32.74817258 |
| SKA1 | 1.387653475 | 4.266747546 | 9.325733882 | 3.34E-19 | 3.06E-18 | 32.71497364 |
| GPR45 | -1.211183561 | 4.351467823 | -9.322383762 | 3.43E-19 | 3.14E-18 | 32.68836394 |
| LRRTM1 | -1.115271202 | 7.519414207 | -9.317629196 | 3.57E-19 | 3.25E-18 | 32.65061047 |
| PLA2G5 | 1.896446758 | 5.505933819 | 9.315996404 | 3.61E-19 | 3.29E-18 | 32.63764849 |
| TSTD1 | 1.6147624 | 4.809121933 | 9.315230651 | 3.63E-19 | 3.30E-18 | 32.63157009 |
| SLC35D3 | -1.224338487 | 2.080223285 | -9.313356952 | 3.69E-19 | 3.35E-18 | 32.6166985 |
| FOXM1 | 1.528348376 | 7.895388036 | 9.29952388 | 4.12E-19 | 3.73E-18 | 32.50697068 |
| SOCS3 | 1.726761404 | 7.444751752 | 9.288586165 | 4.50E-19 | 4.06E-18 | 32.42029148 |
| MYO5C | 1.015396974 | 6.915900445 | 9.284394076 | 4.66E-19 | 4.19E-18 | 32.3870892 |
| SEZ6L | -1.511025042 | 10.57388234 | -9.283553935 | 4.69E-19 | 4.21E-18 | 32.38043637 |
| CPLX2 | -2.119980731 | 10.85923508 | -9.280606681 | 4.80E-19 | 4.31E-18 | 32.35710132 |
| CTSS | 1.039394837 | 9.082248596 | 9.278618385 | 4.88E-19 | 4.37E-18 | 32.34136185 |
| C14orf50 | 1.064989028 | 2.601450032 | 9.275323249 | 5.01E-19 | 4.49E-18 | 32.31528262 |
| CYP2A6 | -1.023447725 | 2.066986157 | -9.274810531 | 5.03E-19 | 4.51E-18 | 32.31122532 |
| VWC2 | -1.365236495 | 5.636833382 | -9.272434824 | 5.13E-19 | 4.59E-18 | 32.2924277 |
| CDCP1 | 1.080850689 | 5.569750618 | 9.26949094 | 5.25E-19 | 4.70E-18 | 32.26913915 |
| HAMP | 1.825489468 | 4.570556607 | 9.267545168 | 5.33E-19 | 4.77E-18 | 32.25374938 |
| PPP4R4 | -1.711435328 | 6.76765768 | -9.263504029 | 5.51E-19 | 4.91E-18 | 32.22179396 |
| PAPPA | -1.495482035 | 5.105395395 | -9.256816949 | 5.81E-19 | 5.18E-18 | 32.16893747 |
| GREB1L | -1.623618338 | 5.099805036 | -9.256169313 | 5.84E-19 | 5.20E-18 | 32.16381981 |
| ISG20 | 1.073621721 | 5.213671704 | 9.251673824 | 6.06E-19 | 5.39E-18 | 32.12830326 |
| SH2D1A | 1.010724716 | 1.920551259 | 9.25145267 | 6.07E-19 | 5.40E-18 | 32.12655635 |
| LAIR1 | 1.118223201 | 8.328667206 | 9.245169493 | 6.38E-19 | 5.66E-18 | 32.07693761 |
| SLC2A4 | -1.335591301 | 5.839493668 | -9.237431831 | 6.79E-19 | 6.01E-18 | 32.01586564 |
| RASGRF1 | -1.577205815 | 8.94286769 | -9.234285814 | 6.96E-19 | 6.16E-18 | 31.99104512 |
| NUP62CL | 1.093976445 | 1.301660728 | 9.218536927 | 7.89E-19 | 6.97E-18 | 31.86688468 |
| IL18 | 1.12834766 | 7.086061016 | 9.212347074 | 8.29E-19 | 7.31E-18 | 31.81812673 |
| FAM46B | 1.396423704 | 3.97954139 | 9.210604137 | 8.41E-19 | 7.40E-18 | 31.80440169 |
| RNASE2 | 1.551933222 | 4.289643318 | 9.206753877 | 8.67E-19 | 7.63E-18 | 31.77408878 |
| NCRNA00176 | -1.359711134 | 5.295732996 | -9.201282018 | 9.06E-19 | 7.96E-18 | 31.73102465 |
| CSTA | 1.601008088 | 2.93791314 | 9.198930049 | 9.23E-19 | 8.10E-18 | 31.71252002 |
| CACNA1I | -1.598962984 | 6.687083102 | -9.195331817 | 9.50E-19 | 8.32E-18 | 31.68421667 |
| PGR | -1.064824516 | 4.570551477 | -9.184320704 | 1.04E-18 | 9.05E-18 | 31.59765346 |
| C19orf51 | 1.493380894 | 3.858198846 | 9.183421725 | 1.04E-18 | 9.12E-18 | 31.59058946 |
| ZNF488 | -1.398606352 | 9.549734401 | -9.183061159 | 1.05E-18 | 9.14E-18 | 31.58775635 |
| NMUR2 | -1.585987372 | 3.152386262 | -9.181258785 | 1.06E-18 | 9.26E-18 | 31.57359554 |
| CD300LG | -1.613164772 | 3.617605166 | -9.177670241 | 1.09E-18 | 9.52E-18 | 31.54540715 |
| NEIL3 | 1.543640554 | 3.2292798 | 9.173079344 | 1.13E-18 | 9.87E-18 | 31.50935667 |
| EYA1 | -1.582364489 | 8.467853143 | -9.172583072 | 1.14E-18 | 9.90E-18 | 31.50546042 |
| RNASE4 | 1.074667276 | 6.738408193 | 9.170372653 | 1.16E-18 | 1.01E-17 | 31.48810816 |
| FAM115C | 1.065261457 | 6.124779568 | 9.164667176 | 1.21E-18 | 1.05E-17 | 31.44333277 |
| C2 | 1.233456209 | 7.574833154 | 9.161564761 | 1.24E-18 | 1.08E-17 | 31.41899404 |
| C7orf52 | -1.493206877 | 6.490651043 | -9.15765515 | 1.28E-18 | 1.11E-17 | 31.38833119 |
| MSX2P1 | -1.098870296 | 1.803340618 | -9.150803021 | 1.35E-18 | 1.17E-17 | 31.33461296 |
| LOC389332 | -1.727468959 | 3.53169573 | -9.150729762 | 1.36E-18 | 1.17E-17 | 31.33403879 |
| EMP1 | 1.353834791 | 10.41763963 | 9.148728044 | 1.38E-18 | 1.19E-17 | 31.31835157 |
| MATN2 | 1.02038821 | 10.74956859 | 9.133087241 | 1.56E-18 | 1.34E-17 | 31.19586123 |
| DKFZp686O24166 | -1.3221899 | 6.455235757 | -9.12918253 | 1.61E-18 | 1.38E-17 | 31.16530508 |
| SPON2 | 1.364000583 | 5.218504452 | 9.124472522 | 1.67E-18 | 1.43E-17 | 31.12845962 |
| WWTR1 | 1.211745592 | 8.028811583 | 9.124380242 | 1.67E-18 | 1.43E-17 | 31.12773786 |
| S100A3 | 1.805359927 | 3.731442043 | 9.123136337 | 1.69E-18 | 1.45E-17 | 31.11800939 |
| POLQ | 1.294775789 | 4.20886402 | 9.12228997 | 1.70E-18 | 1.46E-17 | 31.11139057 |
| LOC644165 | -1.132976884 | 4.382683026 | -9.120660555 | 1.72E-18 | 1.47E-17 | 31.09864934 |
| GRID2 | -1.488314588 | 7.162462137 | -9.116415204 | 1.78E-18 | 1.52E-17 | 31.06546045 |
| HCLS1 | 1.046393389 | 9.53584942 | 9.114416052 | 1.81E-18 | 1.55E-17 | 31.04983552 |
| BUB1 | 1.553520742 | 5.877327149 | 9.113587446 | 1.82E-18 | 1.56E-17 | 31.04336004 |
| ZFR2 | -1.940995361 | 5.74016526 | -9.113249626 | 1.83E-18 | 1.56E-17 | 31.04072013 |
| OASL | 1.383936776 | 4.74463088 | 9.111339999 | 1.85E-18 | 1.58E-17 | 31.02579858 |
| WDR63 | 1.518036576 | 2.528813395 | 9.107254369 | 1.91E-18 | 1.63E-17 | 30.99388161 |
| MAK | 1.170360937 | 2.575623019 | 9.099366703 | 2.04E-18 | 1.73E-17 | 30.93229222 |
| GBP6 | 1.154968903 | 2.200359144 | 9.09457681 | 2.12E-18 | 1.80E-17 | 30.89490995 |
| LOC388387 | -1.181906159 | 1.825691426 | -9.093330991 | 2.14E-18 | 1.81E-17 | 30.8851894 |
| NPPA | -1.771930311 | 6.908681529 | -9.090633268 | 2.18E-18 | 1.85E-17 | 30.86414359 |
| HOXB5 | 1.094877407 | 0.729197811 | 9.083048399 | 2.32E-18 | 1.96E-17 | 30.80499565 |
| ZNF98 | -1.46988523 | 3.196976688 | -9.082633855 | 2.33E-18 | 1.97E-17 | 30.801764 |
| LOC374491 | -1.149501169 | 2.485751947 | -9.071836564 | 2.53E-18 | 2.13E-17 | 30.71762924 |
| MYH7 | -1.338096681 | 9.050946271 | -9.071631001 | 2.54E-18 | 2.13E-17 | 30.71602815 |
| MLF1IP | 1.275790862 | 6.37511146 | 9.070694561 | 2.56E-18 | 2.15E-17 | 30.70873473 |
| GATA4 | 1.390385733 | 0.893226666 | 9.066859819 | 2.64E-18 | 2.21E-17 | 30.67887369 |
| OTX1 | 1.40615907 | 3.85096431 | 9.058846598 | 2.81E-18 | 2.34E-17 | 30.61650432 |
| ELAVL4 | -1.453938164 | 7.808798412 | -9.058018182 | 2.83E-18 | 2.36E-17 | 30.61005878 |
| ACPP | 1.314741259 | 2.600544766 | 9.056751827 | 2.85E-18 | 2.38E-17 | 30.60020664 |
| SH2D2A | 1.038038806 | 2.186290757 | 9.052491054 | 2.95E-18 | 2.46E-17 | 30.56706547 |
| DLL3 | -1.753205823 | 11.47252758 | -9.047978619 | 3.06E-18 | 2.54E-17 | 30.53197909 |
| UCP2 | 1.034010179 | 9.167503002 | 9.032803005 | 3.45E-18 | 2.85E-17 | 30.41407403 |
| HLA-DRB5 | 1.648499245 | 8.272325907 | 9.028336344 | 3.57E-18 | 2.95E-17 | 30.3793981 |
| CHRNA1 | 1.903319178 | 3.100772125 | 9.027179935 | 3.60E-18 | 2.97E-17 | 30.37042261 |
| TLR1 | 1.022610322 | 6.479248144 | 9.022574666 | 3.74E-18 | 3.08E-17 | 30.33468693 |
| TMEM179 | -1.494377235 | 4.894504507 | -9.020329145 | 3.80E-18 | 3.13E-17 | 30.31726706 |
| BSN | -1.109280144 | 10.19149496 | -9.018950947 | 3.84E-18 | 3.17E-17 | 30.30657709 |
| GRIN2C | -1.217267036 | 8.44575377 | -9.016189931 | 3.93E-18 | 3.23E-17 | 30.28516489 |
| C10orf55 | 1.027352454 | 1.90498451 | 9.014151717 | 3.99E-18 | 3.28E-17 | 30.2693612 |
| LCK | 1.208567956 | 2.783048119 | 9.008300747 | 4.18E-18 | 3.43E-17 | 30.22400891 |
| GZMH | 1.237878846 | 1.863278268 | 9.004740068 | 4.30E-18 | 3.52E-17 | 30.19641967 |
| LCNL1 | -1.654479491 | 5.696663978 | -8.992915409 | 4.72E-18 | 3.85E-17 | 30.10485525 |
| CACNA1H | -1.111906226 | 7.889794425 | -8.986707447 | 4.95E-18 | 4.03E-17 | 30.05681873 |
| MOCOS | 1.012300601 | 2.046622055 | 8.986321476 | 4.97E-18 | 4.04E-17 | 30.05383292 |
| MELK | 1.764387839 | 4.918508954 | 8.983989514 | 5.06E-18 | 4.11E-17 | 30.03579523 |
| HLA-DQB1 | 1.763893383 | 8.047952838 | 8.978747186 | 5.27E-18 | 4.28E-17 | 29.99525831 |
| SIX6 | 1.712589419 | 1.156941832 | 8.978160947 | 5.30E-18 | 4.29E-17 | 29.99072622 |
| GCK | 1.265569014 | 4.924243021 | 8.978091289 | 5.30E-18 | 4.29E-17 | 29.99018772 |
| C6orf118 | 1.287024196 | 3.333692114 | 8.965890575 | 5.83E-18 | 4.71E-17 | 29.89591578 |
| ERC2 | -1.015973483 | 7.607758172 | -8.961094003 | 6.06E-18 | 4.88E-17 | 29.85887934 |
| TOX3 | -1.275947819 | 8.435636995 | -8.955721265 | 6.32E-18 | 5.08E-17 | 29.81741117 |
| DLK2 | -1.06152746 | 4.983530376 | -8.955422547 | 6.33E-18 | 5.09E-17 | 29.81510612 |
| FCER1G | 1.046431185 | 8.365069143 | 8.951972989 | 6.51E-18 | 5.22E-17 | 29.78849172 |
| HLA-DQB2 | 1.704023061 | 2.931879875 | 8.945941583 | 6.82E-18 | 5.46E-17 | 29.74197549 |
| IGFN1 | -2.316293935 | 6.521157697 | -8.923592679 | 8.12E-18 | 6.46E-17 | 29.56981219 |
| CENPK | 1.427040337 | 4.485917198 | 8.912537184 | 8.85E-18 | 7.02E-17 | 29.48476291 |
| CCL5 | 1.400207805 | 4.935587834 | 8.911709451 | 8.91E-18 | 7.06E-17 | 29.4783983 |
| GAD1 | -1.299844633 | 9.843534116 | -8.906868034 | 9.26E-18 | 7.31E-17 | 29.4411803 |
| KCNK7 | -1.090529338 | 4.996820147 | -8.902531361 | 9.57E-18 | 7.56E-17 | 29.40785499 |
| MRC1 | -1.575422305 | 6.520600005 | -8.902436683 | 9.58E-18 | 7.56E-17 | 29.40712757 |
| SUSD2 | 1.019961835 | 5.772356464 | 8.897587011 | 9.95E-18 | 7.85E-17 | 29.36987443 |
| CNGA3 | 1.770193642 | 6.843547104 | 8.896082407 | 1.01E-17 | 7.93E-17 | 29.3583197 |
| FCGR2B | 1.611345181 | 3.665810213 | 8.892425701 | 1.04E-17 | 8.15E-17 | 29.3302437 |
| FAM181A | 1.781695226 | 6.130214899 | 8.88409144 | 1.11E-17 | 8.67E-17 | 29.26628515 |
| AMIGO2 | 1.229945778 | 5.960637245 | 8.880097804 | 1.14E-17 | 8.94E-17 | 29.23565284 |
| C21orf131 | -1.222472052 | 7.479837196 | -8.878838107 | 1.15E-17 | 9.02E-17 | 29.2259927 |
| LAMP3 | 1.083330701 | 3.07104599 | 8.876321653 | 1.17E-17 | 9.19E-17 | 29.20669795 |
| MME | -1.514375564 | 4.123693102 | -8.871732524 | 1.22E-17 | 9.51E-17 | 29.17152142 |
| FLJ16779 | -1.36908988 | 11.52142537 | -8.868839334 | 1.24E-17 | 9.72E-17 | 29.14935141 |
| ACBD7 | -1.375692388 | 9.364222941 | -8.861441504 | 1.32E-17 | 1.03E-16 | 29.09268719 |
| CD44 | 1.299122196 | 11.61159996 | 8.858798546 | 1.35E-17 | 1.05E-16 | 29.07245166 |
| MAOB | 1.86639188 | 9.958470437 | 8.858036255 | 1.35E-17 | 1.05E-16 | 29.06661607 |
| TK1 | 1.21607063 | 6.051512881 | 8.854710907 | 1.39E-17 | 1.08E-16 | 29.04116377 |
| C3orf52 | 1.033313142 | 1.796183278 | 8.854342228 | 1.39E-17 | 1.08E-16 | 29.03834232 |
| NUSAP1 | 1.45076736 | 7.49665612 | 8.847818677 | 1.47E-17 | 1.14E-16 | 28.98843274 |
| WNT16 | 1.474781636 | 2.284943454 | 8.844161378 | 1.51E-17 | 1.17E-16 | 28.96046371 |
| RHBDL3 | -1.011975269 | 10.04161776 | -8.843178122 | 1.52E-17 | 1.18E-16 | 28.95294575 |
| PTK6 | -1.011430333 | 5.012887414 | -8.84149023 | 1.54E-17 | 1.19E-16 | 28.94004158 |
| DAPP1 | 1.11212121 | 4.301522743 | 8.84075115 | 1.55E-17 | 1.20E-16 | 28.93439179 |
| CLEC7A | 1.145936347 | 6.326999463 | 8.837253377 | 1.59E-17 | 1.23E-16 | 28.90765821 |
| C11orf20 | -1.127629892 | 3.965780699 | -8.830473306 | 1.68E-17 | 1.29E-16 | 28.85586005 |
| TRAF3IP3 | 1.099836024 | 5.020522927 | 8.829333513 | 1.69E-17 | 1.31E-16 | 28.84715516 |
| BRIP1 | 1.370892422 | 4.024372074 | 8.817848388 | 1.85E-17 | 1.42E-16 | 28.75948635 |
| LDLRAD2 | 1.087141471 | 3.738548747 | 8.817613989 | 1.85E-17 | 1.42E-16 | 28.75769799 |
| KLRC4 | -1.688649007 | 4.577259976 | -8.8174979 | 1.85E-17 | 1.42E-16 | 28.7568123 |
| KRT80 | 1.049499964 | 1.164478569 | 8.81395142 | 1.91E-17 | 1.46E-16 | 28.72975886 |
| LGR6 | 2.294250541 | 4.171208768 | 8.812668069 | 1.92E-17 | 1.48E-16 | 28.7199711 |
| MYH15 | -1.186617739 | 6.528548474 | -8.804000316 | 2.06E-17 | 1.57E-16 | 28.65389201 |
| CD7 | 1.047090319 | 3.650906461 | 8.802936636 | 2.08E-17 | 1.59E-16 | 28.64578628 |
| DAPL1 | -1.752365587 | 4.410677682 | -8.798856968 | 2.14E-17 | 1.63E-16 | 28.61470401 |
| PABPC1L | 1.122514183 | 6.945039704 | 8.794351469 | 2.22E-17 | 1.69E-16 | 28.58038971 |
| GLIS3 | 1.105341368 | 8.898232356 | 8.78702236 | 2.35E-17 | 1.79E-16 | 28.52459817 |
| DNAH9 | 1.214210822 | 6.98725482 | 8.781296527 | 2.45E-17 | 1.86E-16 | 28.48103509 |
| ROR2 | 1.270603088 | 3.504208912 | 8.776538171 | 2.55E-17 | 1.93E-16 | 28.44484863 |
| SLC14A2 | -1.783438142 | 3.634759786 | -8.770496312 | 2.67E-17 | 2.02E-16 | 28.39892219 |
| DSCAM | -1.03575377 | 10.00979867 | -8.768336734 | 2.71E-17 | 2.05E-16 | 28.38251208 |
| C1QB | 1.123535703 | 11.36516067 | 8.767215349 | 2.74E-17 | 2.07E-16 | 28.37399212 |
| SSTR5 | -1.22173193 | 2.319411139 | -8.766792878 | 2.74E-17 | 2.07E-16 | 28.37078251 |
| FYB | 1.032255325 | 8.451284681 | 8.766648901 | 2.75E-17 | 2.07E-16 | 28.36968871 |
| SFTPC | -1.223785584 | 4.651061006 | -8.752784394 | 3.06E-17 | 2.30E-16 | 28.26442156 |
| SVOPL | 1.028182826 | 1.014245475 | 8.747256741 | 3.19E-17 | 2.40E-16 | 28.22248676 |
| MYBL2 | 1.92571538 | 5.905235594 | 8.743817352 | 3.28E-17 | 2.46E-16 | 28.19640416 |
| KRT75 | 1.020380598 | 0.659181589 | 8.735223685 | 3.50E-17 | 2.62E-16 | 28.13126721 |
| WIPF3 | 1.372520066 | 4.52152448 | 8.730962672 | 3.62E-17 | 2.71E-16 | 28.09898778 |
| PIWIL4 | 1.127056497 | 4.892778413 | 8.726138422 | 3.75E-17 | 2.81E-16 | 28.06245558 |
| PTGER4 | 1.085052094 | 5.102558867 | 8.723310508 | 3.84E-17 | 2.87E-16 | 28.04104781 |
| GJD2 | -1.611624845 | 2.415909524 | -8.719667419 | 3.94E-17 | 2.95E-16 | 28.01347659 |
| SH3GL3 | -1.310494069 | 7.917855491 | -8.718855855 | 3.97E-17 | 2.96E-16 | 28.00733576 |
| MYO1G | 1.260827743 | 4.246113994 | 8.716726545 | 4.03E-17 | 3.01E-16 | 27.991226 |
| CYTIP | 1.02961405 | 5.251949722 | 8.714630858 | 4.10E-17 | 3.06E-16 | 27.97537347 |
| RHOH | 1.056786453 | 4.052529702 | 8.713950057 | 4.12E-17 | 3.07E-16 | 27.97022425 |
| CCR2 | 1.333287959 | 2.166948834 | 8.702766713 | 4.49E-17 | 3.34E-16 | 27.88568196 |
| NMNAT3 | 1.324204387 | 5.874433103 | 8.69926486 | 4.61E-17 | 3.42E-16 | 27.85922565 |
| TNR | -1.791335181 | 10.51323805 | -8.698855471 | 4.63E-17 | 3.43E-16 | 27.85613326 |
| NEUROD1 | -1.476636345 | 6.046431085 | -8.692070987 | 4.88E-17 | 3.61E-16 | 27.80490109 |
| SLC25A43 | 1.083126758 | 6.740454509 | 8.688913413 | 4.99E-17 | 3.70E-16 | 27.78106717 |
| CYP27B1 | 1.199800654 | 3.351441741 | 8.687142826 | 5.06E-17 | 3.75E-16 | 27.76770527 |
| CCNB2 | 1.690238987 | 5.432283661 | 8.686293896 | 5.10E-17 | 3.77E-16 | 27.76129946 |
| PRCD | -1.001319464 | 5.394054949 | -8.681198633 | 5.30E-17 | 3.91E-16 | 27.72286165 |
| WT1 | 1.219827255 | 0.797839611 | 8.67404486 | 5.60E-17 | 4.13E-16 | 27.668923 |
| ESPL1 | 1.620196613 | 5.238285687 | 8.670866248 | 5.74E-17 | 4.22E-16 | 27.64496721 |
| RAB36 | 1.323326534 | 6.483836866 | 8.668458531 | 5.84E-17 | 4.30E-16 | 27.62682564 |
| HJURP | 1.590749584 | 5.264722514 | 8.667130028 | 5.90E-17 | 4.34E-16 | 27.61681729 |
| NCAPG | 1.691216052 | 5.90792892 | 8.663892195 | 6.05E-17 | 4.44E-16 | 27.59242967 |
| VAT1L | -1.329558181 | 10.19820593 | -8.663411638 | 6.07E-17 | 4.45E-16 | 27.58881065 |
| DPP10 | -1.154664268 | 9.008002148 | -8.662162517 | 6.13E-17 | 4.49E-16 | 27.57940437 |
| CTSW | 1.216993927 | 2.780910131 | 8.660843791 | 6.19E-17 | 4.54E-16 | 27.56947502 |
| TFEC | 1.010489434 | 6.27071233 | 8.656275895 | 6.41E-17 | 4.69E-16 | 27.53508976 |
| ADAMTS20 | -2.061425117 | 3.591362817 | -8.646056705 | 6.93E-17 | 5.07E-16 | 27.45821262 |
| CD180 | 1.074857703 | 5.797456572 | 8.63889551 | 7.32E-17 | 5.34E-16 | 27.40438045 |
| LOC150622 | -1.121345229 | 9.233484044 | -8.636676754 | 7.45E-17 | 5.43E-16 | 27.38770834 |
| IL28RA | 1.161503689 | 5.660048441 | 8.635896459 | 7.49E-17 | 5.46E-16 | 27.38184583 |
| ADAM33 | 1.196536802 | 5.318463178 | 8.632844254 | 7.67E-17 | 5.58E-16 | 27.35891778 |
| SCML4 | 1.035468125 | 1.96378765 | 8.632086699 | 7.71E-17 | 5.60E-16 | 27.35322799 |
| GPR17 | -1.70919219 | 10.10606834 | -8.630951188 | 7.78E-17 | 5.65E-16 | 27.34470017 |
| SDC1 | 1.105614745 | 6.081767232 | 8.622979894 | 8.27E-17 | 5.99E-16 | 27.28485832 |
| VWA5B2 | -1.318735307 | 6.793285771 | -8.616460294 | 8.69E-17 | 6.29E-16 | 27.23594519 |
| PCDHA5 | -1.273474807 | 5.151100382 | -8.608210972 | 9.25E-17 | 6.68E-16 | 27.17409435 |
| SYNC | 1.09841269 | 7.197771694 | 8.607590355 | 9.30E-17 | 6.71E-16 | 27.16944294 |
| MS4A6A | 1.470513818 | 8.117887507 | 8.601779285 | 9.72E-17 | 7.00E-16 | 27.12590219 |
| PITX2 | 1.218028967 | 0.8093978 | 8.599192759 | 9.91E-17 | 7.14E-16 | 27.10652911 |
| F5 | -2.292760838 | 7.620327915 | -8.598310926 | 9.98E-17 | 7.17E-16 | 27.09992517 |
| RLTPR | -1.360057836 | 6.236107106 | -8.597938754 | 1.00E-16 | 7.19E-16 | 27.09713817 |
| MGP | 1.588484186 | 8.057376427 | 8.594552332 | 1.03E-16 | 7.37E-16 | 27.0717832 |
| HAPLN1 | -1.687845344 | 8.367685032 | -8.593643899 | 1.03E-16 | 7.42E-16 | 27.06498281 |
| LILRB1 | 1.090666188 | 6.853290075 | 8.588283272 | 1.08E-16 | 7.72E-16 | 27.02486487 |
| RAPGEF4 | -1.059482213 | 10.32344394 | -8.577915088 | 1.17E-16 | 8.33E-16 | 26.94732432 |
| NETO1 | -1.331711921 | 7.094357665 | -8.576745942 | 1.18E-16 | 8.40E-16 | 26.93858502 |
| ARHGAP28 | -1.102237833 | 5.320109296 | -8.57531224 | 1.19E-16 | 8.48E-16 | 26.92786939 |
| SLAMF8 | 1.256796525 | 5.68498203 | 8.570471632 | 1.23E-16 | 8.79E-16 | 26.89170007 |
| AKR1C2 | -1.202453878 | 5.609466102 | -8.568207518 | 1.25E-16 | 8.93E-16 | 26.87478771 |
| CD93 | 1.173540156 | 8.36571133 | 8.564113679 | 1.29E-16 | 9.19E-16 | 26.84421625 |
| NCF1C | 1.176717252 | 3.80592596 | 8.560060977 | 1.33E-16 | 9.46E-16 | 26.81396275 |
| LRRN4CL | 1.537008006 | 5.155102176 | 8.548178612 | 1.46E-16 | 1.03E-15 | 26.72532236 |
| CDC25C | 1.391760547 | 3.694751945 | 8.536294888 | 1.60E-16 | 1.13E-15 | 26.636764 |
| LUM | 1.308945074 | 5.53819199 | 8.534930521 | 1.61E-16 | 1.14E-15 | 26.62660254 |
| SYT9 | -1.066074094 | 7.256915122 | -8.531086479 | 1.66E-16 | 1.17E-15 | 26.59797964 |
| RRM2 | 1.78676106 | 6.578041112 | 8.520222827 | 1.80E-16 | 1.26E-15 | 26.51714065 |
| FLJ43390 | -1.557288802 | 7.003447074 | -8.513494278 | 1.90E-16 | 1.33E-15 | 26.46711066 |
| ST8SIA2 | -1.695585828 | 5.636064526 | -8.510046667 | 1.95E-16 | 1.36E-15 | 26.4414875 |
| OPLAH | 1.037368304 | 6.418491601 | 8.509606379 | 1.95E-16 | 1.36E-15 | 26.43821578 |
| EPCAM | -1.015920789 | 3.901591592 | -8.505594858 | 2.01E-16 | 1.40E-15 | 26.40841252 |
| PGAM2 | 1.181517343 | 7.364049832 | 8.498283004 | 2.13E-16 | 1.48E-15 | 26.35411684 |
| SYCE2 | -1.187815487 | 4.95013921 | -8.495180568 | 2.18E-16 | 1.52E-15 | 26.33108966 |
| CYTL1 | 1.101353199 | 5.990891389 | 8.494151694 | 2.20E-16 | 1.53E-15 | 26.32345445 |
| CKAP2L | 1.541683195 | 4.797555096 | 8.489003489 | 2.28E-16 | 1.59E-15 | 26.28526038 |
| ROR1 | 1.019114854 | 3.655920908 | 8.484136091 | 2.37E-16 | 1.64E-15 | 26.24916559 |
| KCNH8 | -1.126449133 | 7.656682985 | -8.47981039 | 2.45E-16 | 1.69E-15 | 26.21710088 |
| C17orf87 | 1.037355295 | 3.775651877 | 8.475137567 | 2.53E-16 | 1.75E-15 | 26.18247691 |
| OPCML | -1.154826491 | 9.704235522 | -8.472172654 | 2.59E-16 | 1.79E-15 | 26.16051539 |
| C8orf4 | 1.233239156 | 7.459251618 | 8.468986762 | 2.65E-16 | 1.83E-15 | 26.13692349 |
| NEU4 | -1.278983377 | 9.83113136 | -8.465487435 | 2.73E-16 | 1.88E-15 | 26.11101826 |
| EXO1 | 1.397393947 | 4.537235797 | 8.462441888 | 2.79E-16 | 1.92E-15 | 26.08847888 |
| SGOL1 | 1.391601622 | 3.58424998 | 8.46057987 | 2.83E-16 | 1.95E-15 | 26.07470153 |
| GTSE1 | 1.376294567 | 5.703484221 | 8.456002954 | 2.93E-16 | 2.01E-15 | 26.04084595 |
| PSTPIP1 | -1.129381897 | 7.344779441 | -8.454723478 | 2.96E-16 | 2.03E-15 | 26.03138409 |
| AGXT2L1 | -1.761916925 | 10.64508746 | -8.453980547 | 2.97E-16 | 2.04E-15 | 26.02589053 |
| ERCC6L | 1.060042533 | 3.694632735 | 8.447343252 | 3.12E-16 | 2.14E-15 | 25.97682761 |
| ITGB3 | 1.191415949 | 4.670613802 | 8.4464966 | 3.14E-16 | 2.15E-15 | 25.97057124 |
| SNCG | -1.467857851 | 8.052324555 | -8.445083482 | 3.18E-16 | 2.17E-15 | 25.96013 |
| ISG15 | 1.197591066 | 8.709513858 | 8.443766313 | 3.21E-16 | 2.19E-15 | 25.9503989 |
| CP | 1.927533567 | 7.683947011 | 8.442496627 | 3.24E-16 | 2.21E-15 | 25.94101967 |
| CSMD1 | -1.187930614 | 8.448149601 | -8.441057653 | 3.27E-16 | 2.24E-15 | 25.9303912 |
| HPCAL4 | -1.488104731 | 9.841653702 | -8.438960514 | 3.33E-16 | 2.27E-15 | 25.91490386 |
| GAL3ST1 | -1.099753099 | 7.704122622 | -8.430578361 | 3.54E-16 | 2.41E-15 | 25.85303077 |
| GRIA1 | -1.280705901 | 11.23319225 | -8.427949458 | 3.61E-16 | 2.46E-15 | 25.833635 |
| BIRC5 | 1.66112992 | 5.640859489 | 8.421546395 | 3.79E-16 | 2.57E-15 | 25.78641299 |
| CELF4 | -1.588253884 | 8.402377854 | -8.418783509 | 3.87E-16 | 2.62E-15 | 25.76604532 |
| C2orf39 | 1.82809605 | 2.455936858 | 8.408582857 | 4.18E-16 | 2.83E-15 | 25.69089102 |
| TREM2 | 1.13126188 | 9.395887049 | 8.401725465 | 4.40E-16 | 2.97E-15 | 25.6404072 |
| KLHDC7B | 1.073294679 | 2.826886113 | 8.394660152 | 4.64E-16 | 3.13E-15 | 25.58842524 |
| TMEM176A | 1.117197253 | 8.159392649 | 8.393837963 | 4.66E-16 | 3.14E-15 | 25.58237826 |
| SYN1 | -1.427568362 | 10.28252205 | -8.39070999 | 4.78E-16 | 3.22E-15 | 25.55937694 |
| C6orf165 | 1.007101794 | 3.800209352 | 8.384095573 | 5.02E-16 | 3.37E-15 | 25.51075966 |
| SLC37A2 | 1.016399178 | 7.247575156 | 8.377231443 | 5.28E-16 | 3.54E-15 | 25.46033762 |
| TROAP | 1.606959121 | 4.886294661 | 8.372058433 | 5.49E-16 | 3.68E-15 | 25.42235874 |
| GGTA1 | -1.02197441 | 9.604592084 | -8.358667439 | 6.07E-16 | 4.05E-15 | 25.32412814 |
| HLA-DQA2 | 1.925071538 | 4.954461891 | 8.358632182 | 6.07E-16 | 4.05E-15 | 25.32386967 |
| ITGA4 | 1.135415019 | 5.686693832 | 8.356081245 | 6.18E-16 | 4.13E-15 | 25.30517066 |
| ITGB4 | 1.35148225 | 10.28718459 | 8.352061871 | 6.37E-16 | 4.25E-15 | 25.27571642 |
| SCIN | 1.58028299 | 8.121663445 | 8.338307488 | 7.06E-16 | 4.69E-15 | 25.17500471 |
| HIST1H3E | 1.096169751 | 3.556703474 | 8.33718045 | 7.12E-16 | 4.73E-15 | 25.16675795 |
| SCN2B | -1.227312193 | 8.831391321 | -8.335206155 | 7.22E-16 | 4.79E-15 | 25.15231368 |
| EN2 | 1.378540143 | 4.381321522 | 8.332970403 | 7.34E-16 | 4.87E-15 | 25.13595968 |
| HAND2 | 1.918881706 | 2.159755312 | 8.326538902 | 7.70E-16 | 5.09E-15 | 25.08893334 |
| GLP1R | -1.846329809 | 3.16017745 | -8.324816023 | 7.80E-16 | 5.15E-15 | 25.07634054 |
| ACSL6 | -1.016543017 | 9.295254939 | -8.324358598 | 7.83E-16 | 5.17E-15 | 25.07299749 |
| FEZF2 | -1.35106352 | 5.245612275 | -8.30945724 | 8.75E-16 | 5.75E-15 | 24.96416829 |
| ATP2B3 | -1.813228258 | 6.939331421 | -8.306481748 | 8.94E-16 | 5.87E-15 | 24.94245512 |
| IQSEC3 | -1.712295886 | 7.499440733 | -8.306206353 | 8.96E-16 | 5.88E-15 | 24.94044577 |
| KIAA1244 | -1.139370997 | 8.9529446 | -8.304755488 | 9.06E-16 | 5.93E-15 | 24.92986072 |
| NEUROD4 | -1.795020952 | 3.378658534 | -8.300637706 | 9.34E-16 | 6.11E-15 | 24.89982638 |
| FAM26F | 1.095169088 | 4.638190157 | 8.299193253 | 9.44E-16 | 6.18E-15 | 24.88929349 |
| NUF2 | 1.173166767 | 5.688794023 | 8.293436274 | 9.85E-16 | 6.44E-15 | 24.84732771 |
| LOC400043 | 1.113547117 | 6.690162853 | 8.289193493 | 1.02E-15 | 6.64E-15 | 24.81641393 |
| CD6 | 1.01588428 | 3.580424166 | 8.287160374 | 1.03E-15 | 6.73E-15 | 24.80160448 |
| OLR1 | 1.329516543 | 8.52069988 | 8.283054595 | 1.06E-15 | 6.93E-15 | 24.77170599 |
| LOXL2 | 1.096905151 | 7.61000834 | 8.280348792 | 1.09E-15 | 7.06E-15 | 24.75200836 |
| NOTUM | -1.14960091 | 4.344053795 | -8.271082894 | 1.16E-15 | 7.55E-15 | 24.68459191 |
| DGKB | -1.086291008 | 9.059194732 | -8.265941112 | 1.21E-15 | 7.83E-15 | 24.64720637 |
| PDIA2 | -1.30806774 | 6.611886179 | -8.265066243 | 1.22E-15 | 7.87E-15 | 24.64084702 |
| PAK3 | -1.475591545 | 5.716934388 | -8.262312104 | 1.24E-15 | 8.02E-15 | 24.62083076 |
| TYMS | 1.09860423 | 8.039970966 | 8.25271496 | 1.33E-15 | 8.58E-15 | 24.5511214 |
| SHANK1 | -1.366645152 | 8.180302056 | -8.251057313 | 1.35E-15 | 8.68E-15 | 24.53908725 |
| SNCB | -1.768446564 | 9.163855058 | -8.245551398 | 1.40E-15 | 9.02E-15 | 24.49912879 |
| GPR156 | 1.010885455 | 3.024427315 | 8.245083141 | 1.41E-15 | 9.04E-15 | 24.49573142 |
| CD300LF | 1.077359597 | 4.432131601 | 8.239604632 | 1.47E-15 | 9.40E-15 | 24.4559938 |
| S100A10 | 1.148619997 | 9.80458321 | 8.237282454 | 1.49E-15 | 9.55E-15 | 24.43915629 |
| AJAP1 | -1.084416202 | 6.309703131 | -8.233200117 | 1.54E-15 | 9.83E-15 | 24.40956511 |
| TMEM196 | -1.797680394 | 5.405290381 | -8.231188469 | 1.56E-15 | 9.97E-15 | 24.39498762 |
| OIP5 | 1.047318594 | 4.234711002 | 8.223169797 | 1.66E-15 | 1.06E-14 | 24.33690707 |
| GRIK1 | 1.593831978 | 6.088593665 | 8.222809491 | 1.66E-15 | 1.06E-14 | 24.33429833 |
| GPIHBP1 | -1.097312657 | 7.826165518 | -8.218091733 | 1.72E-15 | 1.09E-14 | 24.30014822 |
| TLX1 | -2.32729706 | 4.410509808 | -8.213609216 | 1.78E-15 | 1.13E-14 | 24.26771481 |
| ASPHD1 | -1.007832212 | 8.80747204 | -8.212397943 | 1.79E-15 | 1.14E-14 | 24.25895293 |
| NCRNA00087 | -1.064151662 | 7.291210202 | -8.210406982 | 1.82E-15 | 1.16E-14 | 24.24455323 |
| FBLN7 | 1.467740397 | 5.200394459 | 8.196652101 | 2.01E-15 | 1.28E-14 | 24.14514361 |
| SOD3 | 1.226765438 | 6.320498179 | 8.195225276 | 2.03E-15 | 1.29E-14 | 24.13483893 |
| FAP | 1.277615346 | 4.32685185 | 8.194728833 | 2.04E-15 | 1.29E-14 | 24.13125389 |
| SOX3 | -1.254826946 | 6.824272473 | -8.192579371 | 2.07E-15 | 1.31E-14 | 24.11573358 |
| PDCD1 | 1.077046111 | 1.951286582 | 8.186105453 | 2.17E-15 | 1.37E-14 | 24.06900711 |
| HSPA6 | 1.363935384 | 6.158968075 | 8.181661786 | 2.25E-15 | 1.42E-14 | 24.03695068 |
| EIF4E1B | -1.467771363 | 2.573199211 | -8.17801628 | 2.31E-15 | 1.46E-14 | 24.01066212 |
| PCDH11Y | -1.612503801 | 3.945216749 | -8.177069041 | 2.32E-15 | 1.47E-14 | 24.00383284 |
| KLHL4 | 1.146832726 | 7.619193263 | 8.176629977 | 2.33E-15 | 1.47E-14 | 24.00066753 |
| PHEX | 1.235882537 | 3.432066449 | 8.175811007 | 2.34E-15 | 1.48E-14 | 23.99476376 |
| GPNMB | 1.509557168 | 8.644951581 | 8.158021872 | 2.67E-15 | 1.68E-14 | 23.86663785 |
| CD24 | -1.260293243 | 10.65831392 | -8.156622621 | 2.70E-15 | 1.69E-14 | 23.85656887 |
| SERPINF1 | 1.121406472 | 8.126796642 | 8.154488853 | 2.74E-15 | 1.72E-14 | 23.84121686 |
| TLX1NB | -1.94094192 | 2.606884347 | -8.142695461 | 2.99E-15 | 1.87E-14 | 23.7564216 |
| ARSJ | 1.15462341 | 5.470411621 | 8.136206407 | 3.13E-15 | 1.95E-14 | 23.70980512 |
| LCN12 | -1.043396261 | 4.422164069 | -8.1327236 | 3.21E-15 | 2.00E-14 | 23.6847969 |
| JAK3 | 1.068437653 | 6.245088538 | 8.129991874 | 3.28E-15 | 2.04E-14 | 23.66518756 |
| MMP9 | 1.826998532 | 3.163466151 | 8.11858446 | 3.56E-15 | 2.21E-14 | 23.58335574 |
| PDZK1IP1 | 1.127593296 | 2.141415358 | 8.113358626 | 3.70E-15 | 2.30E-14 | 23.54589744 |
| RYR3 | 1.454314164 | 6.875944373 | 8.107973933 | 3.85E-15 | 2.38E-14 | 23.50731988 |
| TFPI | 1.396916491 | 7.125448142 | 8.106158861 | 3.90E-15 | 2.41E-14 | 23.49432061 |
| NOX4 | 1.157656866 | 3.539608826 | 8.106033195 | 3.90E-15 | 2.42E-14 | 23.49342069 |
| RBM11 | -1.206629407 | 4.452675461 | -8.101793041 | 4.03E-15 | 2.49E-14 | 23.46306244 |
| CCDC37 | 1.294013002 | 1.27913874 | 8.101638652 | 4.03E-15 | 2.49E-14 | 23.4619573 |
| RET | -1.45075445 | 7.567578332 | -8.098813776 | 4.12E-15 | 2.54E-14 | 23.44173912 |
| DCDC2 | 1.015237395 | 4.05088121 | 8.098731154 | 4.12E-15 | 2.54E-14 | 23.44114786 |
| SNX22 | -1.113958632 | 10.43479119 | -8.081350035 | 4.67E-15 | 2.87E-14 | 23.31686853 |
| AKR1C1 | -1.280556653 | 7.964705098 | -8.06739558 | 5.17E-15 | 3.16E-14 | 23.2172399 |
| MYT1L | -2.050734733 | 7.770896298 | -8.066920577 | 5.19E-15 | 3.17E-14 | 23.21385093 |
| KIAA0101 | 1.41917319 | 5.170704047 | 8.063307679 | 5.33E-15 | 3.25E-14 | 23.18807929 |
| NEK2 | 1.381697411 | 4.731265817 | 8.063286672 | 5.33E-15 | 3.25E-14 | 23.18792946 |
| MYH6 | -1.205863754 | 4.479653491 | -8.062250334 | 5.37E-15 | 3.27E-14 | 23.18053868 |
| SNTG1 | -1.207552398 | 6.052600739 | -8.061302504 | 5.41E-15 | 3.29E-14 | 23.17377975 |
| ?\|90288 | 1.051501369 | 3.398748375 | 8.060677124 | 5.43E-15 | 3.30E-14 | 23.16932053 |
| WDR38 | 1.594572057 | 2.191395433 | 8.059962272 | 5.46E-15 | 3.32E-14 | 23.16422367 |
| IRX2 | -2.163564785 | 5.866530221 | -8.057687257 | 5.55E-15 | 3.37E-14 | 23.14800524 |
| SLCO1A2 | -1.091406289 | 10.12569339 | -8.055169184 | 5.65E-15 | 3.43E-14 | 23.13005819 |
| CTHRC1 | 1.137755784 | 4.485113442 | 8.050221213 | 5.86E-15 | 3.55E-14 | 23.09480519 |
| MUM1L1 | -1.329424866 | 5.205335999 | -8.048386495 | 5.94E-15 | 3.60E-14 | 23.08173755 |
| VWC2L | -1.491594046 | 2.35374185 | -8.048364357 | 5.94E-15 | 3.60E-14 | 23.08157989 |
| ACVR1C | -1.241281575 | 5.925917583 | -8.041633217 | 6.23E-15 | 3.77E-14 | 23.03365773 |
| CNTNAP5 | -1.539340127 | 5.494227731 | -8.017226329 | 7.44E-15 | 4.47E-14 | 22.860154 |
| HTR6 | -1.172844111 | 2.008843176 | -8.007696074 | 7.97E-15 | 4.78E-14 | 22.79251627 |
| TOP2A | 1.843213788 | 8.261305011 | 8.004216834 | 8.17E-15 | 4.90E-14 | 22.76783909 |
| ZBP1 | 1.132042835 | 2.671484097 | 7.995779202 | 8.68E-15 | 5.19E-14 | 22.70802815 |
| TTK | 1.497705033 | 4.673447629 | 7.989569768 | 9.08E-15 | 5.42E-14 | 22.66404327 |
| LHFPL3 | -1.186965369 | 11.27894255 | -7.988909996 | 9.12E-15 | 5.44E-14 | 22.6593713 |
| TLR8 | 1.159891744 | 4.236871009 | 7.986867752 | 9.26E-15 | 5.52E-14 | 22.64491168 |
| ASPDH | -1.113185322 | 5.644724294 | -7.985368451 | 9.36E-15 | 5.58E-14 | 22.63429806 |
| BSPRY | -1.029091311 | 4.294305698 | -7.971978773 | 1.03E-14 | 6.12E-14 | 22.53958059 |
| SPC24 | 1.336432583 | 2.846946567 | 7.968644215 | 1.06E-14 | 6.26E-14 | 22.51601142 |
| SYT14 | -1.024404532 | 5.803545609 | -7.961956708 | 1.11E-14 | 6.56E-14 | 22.46876621 |
| SPINK8 | 1.049586276 | 1.785149645 | 7.95775028 | 1.14E-14 | 6.75E-14 | 22.43906487 |
| HES5 | -1.575669939 | 6.946974993 | -7.956690463 | 1.15E-14 | 6.80E-14 | 22.43158349 |
| SEMA3D | -1.474456152 | 7.475164753 | -7.95340182 | 1.18E-14 | 6.96E-14 | 22.40837349 |
| SCRT2 | -1.675169679 | 5.784506926 | -7.940453009 | 1.29E-14 | 7.61E-14 | 22.31705831 |
| ATP8A2 | -1.719172999 | 5.44798592 | -7.934643761 | 1.35E-14 | 7.92E-14 | 22.27612907 |
| BAI1 | -1.158468444 | 11.10764965 | -7.930630698 | 1.39E-14 | 8.14E-14 | 22.24786855 |
| GOLGA7B | -1.088794871 | 9.042835834 | -7.927417446 | 1.42E-14 | 8.32E-14 | 22.22524843 |
| BUB1B | 1.505668379 | 5.721575958 | 7.927353108 | 1.42E-14 | 8.32E-14 | 22.22479559 |
| CHODL | 1.085362364 | 4.171723379 | 7.925703601 | 1.44E-14 | 8.41E-14 | 22.21318651 |
| DES | 1.844131862 | 3.142174312 | 7.923314739 | 1.46E-14 | 8.55E-14 | 22.19637727 |
| KCNB2 | -1.528032185 | 3.299559927 | -7.903105974 | 1.69E-14 | 9.82E-14 | 22.05433632 |
| LOC400696 | 1.013888693 | 0.852922461 | 7.902783979 | 1.69E-14 | 9.84E-14 | 22.05207542 |
| GPRC5A | 1.423807158 | 3.314743861 | 7.896444016 | 1.77E-14 | 1.03E-13 | 22.00757363 |
| CDH9 | -1.587845644 | 3.947336954 | -7.89332461 | 1.81E-14 | 1.05E-13 | 21.98568797 |
| STOX1 | -1.156465783 | 8.542986315 | -7.888297382 | 1.88E-14 | 1.09E-13 | 21.95043132 |
| GSG2 | 1.088638595 | 3.587924723 | 7.884847363 | 1.92E-14 | 1.11E-13 | 21.92624601 |
| TAGLN3 | -1.099690626 | 9.935594374 | -7.87661185 | 2.04E-14 | 1.18E-13 | 21.86854689 |
| C6orf141 | 1.201037665 | 2.233558134 | 7.873245864 | 2.09E-14 | 1.20E-13 | 21.8449779 |
| FAM177B | 1.023004801 | 2.19281889 | 7.865838012 | 2.20E-14 | 1.27E-13 | 21.79313513 |
| HOXA11 | 1.790604493 | 1.827090381 | 7.865448225 | 2.21E-14 | 1.27E-13 | 21.79040832 |
| RFPL1S | -1.371181683 | 7.523828593 | -7.859727739 | 2.30E-14 | 1.32E-13 | 21.75040202 |
| SYT4 | -1.871999122 | 7.969535896 | -7.859364152 | 2.31E-14 | 1.32E-13 | 21.74786004 |
| TNFSF12-TNFSF13 | 1.989058553 | 4.152112459 | 7.857416447 | 2.34E-14 | 1.34E-13 | 21.73424443 |
| SELL | -1.715213813 | 9.512942447 | -7.851742889 | 2.44E-14 | 1.39E-13 | 21.69459797 |
| CACNA1B | -1.598476615 | 7.044769564 | -7.84702951 | 2.52E-14 | 1.44E-13 | 21.66167823 |
| RIBC2 | 1.203161629 | 1.853769026 | 7.841890628 | 2.61E-14 | 1.49E-13 | 21.6258043 |
| LYZ | 1.441730154 | 6.780942043 | 7.837020393 | 2.71E-14 | 1.54E-13 | 21.59182278 |
| GALNTL6 | -1.258236796 | 2.674874335 | -7.828429309 | 2.88E-14 | 1.63E-13 | 21.53191979 |
| SYT10 | -1.170415679 | 1.476654392 | -7.823024408 | 2.99E-14 | 1.69E-13 | 21.49425948 |
| PDE1A | -1.192805567 | 6.804187411 | -7.81947589 | 3.06E-14 | 1.74E-13 | 21.4695452 |
| FAM111B | 1.401124556 | 5.007050943 | 7.817097967 | 3.12E-14 | 1.76E-13 | 21.45298865 |
| FAM64A | 1.579446927 | 5.960246364 | 7.81236081 | 3.22E-14 | 1.82E-13 | 21.42001748 |
| PLCH2 | -1.100267705 | 8.076938203 | -7.805441522 | 3.39E-14 | 1.91E-13 | 21.37188662 |
| ANKRD35 | 1.242686039 | 6.972185239 | 7.796472988 | 3.61E-14 | 2.03E-13 | 21.30955098 |
| OLFML2B | 1.068130124 | 7.743969149 | 7.794678842 | 3.65E-14 | 2.05E-13 | 21.29708757 |
| MMEL1 | 1.632499518 | 2.938344791 | 7.78787262 | 3.83E-14 | 2.15E-13 | 21.2498272 |
| PRF1 | 1.066450628 | 4.066569819 | 7.78234798 | 3.99E-14 | 2.23E-13 | 21.21148963 |
| FABP7 | 1.161277957 | 10.03170583 | 7.780120216 | 4.05E-14 | 2.26E-13 | 21.19603639 |
| PIK3CG | 1.053212603 | 5.177637761 | 7.778510703 | 4.10E-14 | 2.29E-13 | 21.18487391 |
| CD48 | 1.394700019 | 4.115927846 | 7.769184636 | 4.37E-14 | 2.44E-13 | 21.12023048 |
| FAM20A | 1.174256678 | 4.792593911 | 7.761981059 | 4.60E-14 | 2.56E-13 | 21.07034085 |
| DHRS2 | -1.601117524 | 4.751065341 | -7.760878053 | 4.64E-14 | 2.58E-13 | 21.062705 |
| C9orf140 | -1.093823183 | 10.0831376 | -7.757466617 | 4.75E-14 | 2.64E-13 | 21.03909388 |
| SIGLEC9 | 1.045726891 | 5.543625575 | 7.749784085 | 5.02E-14 | 2.78E-13 | 20.98595171 |
| SMCP | -1.578460781 | 2.376397361 | -7.744815514 | 5.19E-14 | 2.88E-13 | 20.95160486 |
| LILRB3 | 1.130693502 | 3.973911074 | 7.744274598 | 5.21E-14 | 2.89E-13 | 20.94786665 |
| DPP4 | 1.043139924 | 3.646235834 | 7.742382039 | 5.28E-14 | 2.93E-13 | 20.93478902 |
| DLGAP2 | -1.503589526 | 5.952976438 | -7.732354995 | 5.67E-14 | 3.13E-13 | 20.86554394 |
| C2orf80 | -1.008187631 | 7.402578216 | -7.727548604 | 5.87E-14 | 3.24E-13 | 20.83237691 |
| ANKRD22 | 1.570721842 | 5.808034454 | 7.723905879 | 6.02E-14 | 3.31E-13 | 20.80725074 |
| CBR1 | 1.08937828 | 9.938252521 | 7.723690253 | 6.03E-14 | 3.32E-13 | 20.80576372 |
| NBLA00301 | 1.19751159 | 1.112669452 | 7.721777085 | 6.11E-14 | 3.36E-13 | 20.79257144 |
| AURKB | 1.598031143 | 4.754278693 | 7.716441819 | 6.34E-14 | 3.48E-13 | 20.75579564 |
| C1QL2 | -1.348418735 | 5.648964581 | -7.716150994 | 6.35E-14 | 3.49E-13 | 20.75379157 |
| PSORS1C1 | 1.057990206 | 2.142096714 | 7.715896143 | 6.37E-14 | 3.50E-13 | 20.75203545 |
| SNORD115-26 | -1.21248489 | 3.043918911 | -7.715838552 | 6.37E-14 | 3.50E-13 | 20.75163861 |
| COL27A1 | 1.177709538 | 5.968922888 | 7.713863556 | 6.46E-14 | 3.54E-13 | 20.73803096 |
| SMAD9 | -1.018435556 | 8.263519191 | -7.697516773 | 7.24E-14 | 3.96E-13 | 20.62550798 |
| GPR98 | -1.167252992 | 10.79318043 | -7.696934624 | 7.27E-14 | 3.97E-13 | 20.62150424 |
| NCAPH | 1.160629508 | 5.901001655 | 7.696168996 | 7.31E-14 | 3.99E-13 | 20.61623899 |
| MND1 | 1.049268326 | 3.582396658 | 7.694058559 | 7.42E-14 | 4.05E-13 | 20.60172758 |
| BDKRB2 | 1.219526673 | 4.081943816 | 7.693014301 | 7.47E-14 | 4.08E-13 | 20.5945484 |
| GPR114 | 1.072826903 | 3.538490027 | 7.692849728 | 7.48E-14 | 4.08E-13 | 20.59341705 |
| C14orf86 | 1.168538877 | 3.101362758 | 7.682172521 | 8.06E-14 | 4.38E-13 | 20.52005774 |
| SLC47A1 | 1.093370559 | 5.456539143 | 7.68183268 | 8.08E-14 | 4.39E-13 | 20.51772413 |
| RAB27B | -1.216489627 | 4.570586333 | -7.681152024 | 8.12E-14 | 4.41E-13 | 20.51305048 |
| RXRG | -1.080264037 | 6.633126259 | -7.677586602 | 8.33E-14 | 4.51E-13 | 20.48857426 |
| TFCP2L1 | 1.536972023 | 6.019095298 | 7.667917777 | 8.91E-14 | 4.81E-13 | 20.42224418 |
| SERINC2 | 1.135046568 | 5.670275664 | 7.661664267 | 9.31E-14 | 5.01E-13 | 20.37937912 |
| ST14 | 1.252953936 | 6.226903714 | 7.657377048 | 9.59E-14 | 5.16E-13 | 20.35000814 |
| RAD51 | 1.010125847 | 4.767579413 | 7.645808648 | 1.04E-13 | 5.58E-13 | 20.27082011 |
| SLCO5A1 | -1.144627062 | 4.78493594 | -7.64482151 | 1.05E-13 | 5.62E-13 | 20.26406735 |
| RIMS1 | -1.307306829 | 7.463745376 | -7.643738734 | 1.05E-13 | 5.66E-13 | 20.25666115 |
| DEPDC1B | 1.07768698 | 4.633352309 | 7.637212904 | 1.10E-13 | 5.92E-13 | 20.21204203 |
| RHOD | 1.050687171 | 2.841798128 | 7.636294182 | 1.11E-13 | 5.95E-13 | 20.20576287 |
| C1QTNF4 | -1.138727438 | 6.014445643 | -7.635647889 | 1.12E-13 | 5.98E-13 | 20.20134604 |
| GBP2 | 1.28110733 | 8.822681863 | 7.635058939 | 1.12E-13 | 6.00E-13 | 20.19732135 |
| ACSS3 | 1.11927589 | 7.172113761 | 7.626396019 | 1.19E-13 | 6.37E-13 | 20.13815029 |
| KLRB1 | 1.070352735 | 1.549292458 | 7.615820539 | 1.28E-13 | 6.84E-13 | 20.06598806 |
| LECT1 | 1.413858038 | 2.806286152 | 7.613412304 | 1.30E-13 | 6.95E-13 | 20.04956649 |
| GRIP2 | -1.314490072 | 4.35725749 | -7.604256367 | 1.39E-13 | 7.39E-13 | 19.98717056 |
| ALDH1A3 | 1.675586802 | 3.591513946 | 7.60385018 | 1.39E-13 | 7.41E-13 | 19.98440386 |
| PRDM8 | -1.084961769 | 7.380494814 | -7.602183063 | 1.41E-13 | 7.50E-13 | 19.97304969 |
| BATF | 1.086321886 | 2.956021285 | 7.599871991 | 1.43E-13 | 7.61E-13 | 19.95731304 |
| UBE2C | 1.569466557 | 5.933879658 | 7.589852202 | 1.53E-13 | 8.13E-13 | 19.8891299 |
| MCOLN2 | 1.115548077 | 2.811214686 | 7.58932426 | 1.54E-13 | 8.15E-13 | 19.88553932 |
| CALB1 | -1.515948132 | 5.96938353 | -7.578859362 | 1.66E-13 | 8.75E-13 | 19.81440769 |
| PI15 | 1.078480106 | 4.374979086 | 7.564823128 | 1.82E-13 | 9.61E-13 | 19.71912391 |
| SPRY4 | 1.304214142 | 8.160751114 | 7.557951894 | 1.91E-13 | 1.01E-12 | 19.67253047 |
| PCSK6 | -1.215824358 | 9.772937885 | -7.543852541 | 2.11E-13 | 1.11E-12 | 19.57702935 |
| KCNT1 | -1.684674779 | 6.154183684 | -7.533872669 | 2.26E-13 | 1.18E-12 | 19.50951734 |
| KCNJ3 | -1.360896138 | 5.755903379 | -7.531052695 | 2.30E-13 | 1.20E-12 | 19.49045366 |
| SFTPD | -1.066959181 | 4.488251834 | -7.530745283 | 2.31E-13 | 1.21E-12 | 19.48837583 |
| FREM2 | 1.423407881 | 7.069244811 | 7.524545655 | 2.41E-13 | 1.25E-12 | 19.44648634 |
| CLVS2 | -1.58626206 | 5.142078553 | -7.524434356 | 2.41E-13 | 1.25E-12 | 19.44573457 |
| EPR1 | 1.482524042 | 5.764661124 | 7.523105825 | 2.43E-13 | 1.27E-12 | 19.43676168 |
| VIPR2 | -1.624168135 | 9.64889731 | -7.521817624 | 2.45E-13 | 1.28E-12 | 19.42806238 |
| C1orf158 | 1.427150746 | 1.512055184 | 7.520983357 | 2.47E-13 | 1.28E-12 | 19.42242916 |
| RAB3A | -1.119706125 | 9.288157484 | -7.515968344 | 2.56E-13 | 1.33E-12 | 19.38857685 |
| MMRN1 | 1.129704359 | 5.424150243 | 7.515693208 | 2.56E-13 | 1.33E-12 | 19.38672015 |
| PLEKHG4B | 1.366171614 | 3.319213021 | 7.509987198 | 2.66E-13 | 1.38E-12 | 19.34822655 |
| TLL1 | -1.443121342 | 4.197367035 | -7.509367923 | 2.67E-13 | 1.39E-12 | 19.34405023 |
| RGS7 | -1.143154059 | 7.322836893 | -7.485902394 | 3.14E-13 | 1.62E-12 | 19.18600473 |
| LHX3 | -1.406953447 | 2.439315663 | -7.475574471 | 3.37E-13 | 1.73E-12 | 19.11656947 |
| TRHDE | -1.694949065 | 4.615317852 | -7.474788258 | 3.39E-13 | 1.74E-12 | 19.11128686 |
| DCAF12L2 | -1.263171988 | 2.939905933 | -7.471385265 | 3.47E-13 | 1.77E-12 | 19.08842709 |
| MC4R | -1.042646643 | 2.332390276 | -7.464075872 | 3.65E-13 | 1.86E-12 | 19.03935411 |
| ANXA3 | -1.206231636 | 4.955123321 | -7.460033255 | 3.75E-13 | 1.92E-12 | 19.01222979 |
| C1QL1 | -1.199183581 | 11.35821401 | -7.455890408 | 3.86E-13 | 1.97E-12 | 18.9844452 |
| COL14A1 | 1.326195901 | 6.508444693 | 7.455258764 | 3.88E-13 | 1.97E-12 | 18.98021007 |
| CYBB | 1.002177486 | 9.613283538 | 7.453774146 | 3.92E-13 | 1.99E-12 | 18.97025696 |
| CAMKV | -1.390739777 | 8.563484421 | -7.453727422 | 3.92E-13 | 1.99E-12 | 18.96994374 |
| FAM131C | -1.112878444 | 6.064502035 | -7.45348616 | 3.92E-13 | 1.99E-12 | 18.96832645 |
| C10orf81 | 1.440939555 | 1.946954971 | 7.451629367 | 3.97E-13 | 2.02E-12 | 18.95588084 |
| C11orf88 | 1.064429202 | 1.051507651 | 7.447667472 | 4.08E-13 | 2.07E-12 | 18.92933361 |
| C5orf38 | -1.870968288 | 5.109687199 | -7.430184644 | 4.60E-13 | 2.32E-12 | 18.81232291 |
| NKX3-2 | 1.170832384 | 2.163739206 | 7.426660705 | 4.71E-13 | 2.38E-12 | 18.78876432 |
| CAV1 | 1.012493417 | 8.85566246 | 7.422561461 | 4.85E-13 | 2.45E-12 | 18.76137096 |
| OR2L13 | -1.381894905 | 4.082729346 | -7.421222598 | 4.89E-13 | 2.47E-12 | 18.75242659 |
| TMPRSS3 | 1.365095066 | 2.162476475 | 7.420776891 | 4.90E-13 | 2.47E-12 | 18.7494493 |
| STRA6 | 1.19770996 | 4.25008064 | 7.390262277 | 6.04E-13 | 3.03E-12 | 18.54595616 |
| WNK2 | -1.218522578 | 9.247374227 | -7.376025487 | 6.65E-13 | 3.32E-12 | 18.45124623 |
| PTGFR | -1.234768681 | 4.248209916 | -7.370622845 | 6.90E-13 | 3.44E-12 | 18.41534382 |
| HOXD4 | 1.525195634 | 2.58821541 | 7.361542956 | 7.33E-13 | 3.65E-12 | 18.35505264 |
| NKG7 | 1.071924143 | 3.36996865 | 7.356433643 | 7.59E-13 | 3.78E-12 | 18.32115278 |
| DYNC1I1 | -1.00260416 | 8.606428446 | -7.350417724 | 7.91E-13 | 3.93E-12 | 18.28126201 |
| GABBR2 | -1.157484354 | 10.740646 | -7.347258415 | 8.08E-13 | 4.01E-12 | 18.26032361 |
| CDC45 | 1.482735762 | 4.604049039 | 7.346862696 | 8.10E-13 | 4.02E-12 | 18.25770148 |
| GLIS1 | -1.287430285 | 4.713570989 | -7.343006608 | 8.31E-13 | 4.12E-12 | 18.23215611 |
| FOSL1 | 1.201471775 | 4.604685634 | 7.338090695 | 8.60E-13 | 4.25E-12 | 18.19960546 |
| MTTP | 1.037202361 | 5.70131358 | 7.321762312 | 9.60E-13 | 4.74E-12 | 18.09161377 |
| RASSF9 | 1.177678365 | 2.146603217 | 7.313453037 | 1.02E-12 | 5.00E-12 | 18.03673309 |
| RASSF10 | 1.116407711 | 1.903845904 | 7.303591292 | 1.08E-12 | 5.33E-12 | 17.97166419 |
| MATK | -1.151719891 | 6.766930234 | -7.302973791 | 1.09E-12 | 5.35E-12 | 17.96759222 |
| GABRB1 | -1.098858874 | 5.940870244 | -7.301905454 | 1.10E-12 | 5.38E-12 | 17.96054796 |
| PABPC1L2B | -1.295103908 | 4.918075209 | -7.295847049 | 1.14E-12 | 5.59E-12 | 17.92061666 |
| ZNF727 | -1.220636165 | 4.050023547 | -7.227518976 | 1.81E-12 | 8.66E-12 | 17.47212489 |
| ENPEP | 1.084802938 | 5.682187986 | 7.217593643 | 1.93E-12 | 9.23E-12 | 17.40726229 |
| OR4N4 | -1.12900535 | 1.5346827 | -7.199472364 | 2.18E-12 | 1.04E-11 | 17.28902592 |
| AMH | -1.26468282 | 6.248837543 | -7.197350244 | 2.21E-12 | 1.05E-11 | 17.27519552 |
| PABPC1L2A | -1.087167652 | 3.131137916 | -7.194631438 | 2.25E-12 | 1.07E-11 | 17.2574812 |
| ADAMTS18 | 1.501785043 | 2.565940012 | 7.186431106 | 2.38E-12 | 1.13E-11 | 17.20408515 |
| CCDC135 | 1.232984324 | 3.670857527 | 7.184822876 | 2.40E-12 | 1.14E-11 | 17.19361908 |
| FAM19A1 | -1.54348477 | 5.128662685 | -7.183996536 | 2.41E-12 | 1.14E-11 | 17.18824213 |
| KLRK1 | -1.197164769 | 6.269991271 | -7.177847239 | 2.51E-12 | 1.19E-11 | 17.14824494 |
| MT1M | 1.051504317 | 7.034272799 | 7.169747523 | 2.65E-12 | 1.25E-11 | 17.09560417 |
| TDRD9 | -1.141066009 | 4.558690668 | -7.167965416 | 2.68E-12 | 1.27E-11 | 17.08402861 |
| PRKCB | -1.043152093 | 10.10601316 | -7.164859143 | 2.74E-12 | 1.29E-11 | 17.06385763 |
| KCNC2 | -1.86519239 | 6.176390181 | -7.13567795 | 3.33E-12 | 1.56E-11 | 16.87471452 |
| CACNA2D1 | -1.395276169 | 6.823832731 | -7.110974047 | 3.91E-12 | 1.82E-11 | 16.71508523 |
| SYCE1 | -1.883792334 | 4.522349295 | -7.108397804 | 3.98E-12 | 1.85E-11 | 16.69846441 |
| DDN | -1.668796023 | 8.147724592 | -7.104178741 | 4.09E-12 | 1.90E-11 | 16.67125546 |
| ADAMTS3 | 1.100433741 | 4.347111008 | 7.101446828 | 4.17E-12 | 1.93E-11 | 16.65364427 |
| ESX1 | -1.154178778 | 1.339975035 | -7.099158632 | 4.23E-12 | 1.96E-11 | 16.63889776 |
| ARC | 1.125183379 | 9.125732123 | 7.093495621 | 4.39E-12 | 2.03E-11 | 16.60241866 |
| ESCO2 | 1.191137353 | 4.593634025 | 7.07810253 | 4.86E-12 | 2.24E-11 | 16.50338247 |
| NPM2 | -1.290436273 | 5.710618323 | -7.07444918 | 4.98E-12 | 2.29E-11 | 16.47990343 |
| SYT13 | -1.654948348 | 8.004112323 | -7.061763028 | 5.41E-12 | 2.48E-11 | 16.3984504 |
| GRM1 | -1.03233927 | 6.403699435 | -7.056390971 | 5.60E-12 | 2.57E-11 | 16.3639946 |
| LY6H | -1.169389966 | 8.580861114 | -7.050460707 | 5.83E-12 | 2.66E-11 | 16.32598354 |
| C3orf16 | 1.078429523 | 1.454309711 | 7.038838737 | 6.29E-12 | 2.87E-11 | 16.25156667 |
| FAM180B | 1.042141267 | 2.635967694 | 7.038022263 | 6.32E-12 | 2.88E-11 | 16.24634249 |
| GPR3 | 1.008308644 | 4.923114956 | 7.018263449 | 7.19E-12 | 3.27E-11 | 16.12006824 |
| HRNBP3 | -1.600034977 | 6.997156958 | -7.012634465 | 7.46E-12 | 3.38E-11 | 16.08414812 |
| P2RY8 | 1.095127469 | 4.280917786 | 7.008250385 | 7.67E-12 | 3.48E-11 | 16.05618851 |
| C19orf30 | -1.443813518 | 3.407069044 | -7.005470412 | 7.82E-12 | 3.54E-11 | 16.0384666 |
| COL15A1 | 1.104709472 | 4.638991795 | 7.004935409 | 7.84E-12 | 3.55E-11 | 16.0350567 |
| LRRIQ1 | 1.129446337 | 1.931716122 | 7.003730638 | 7.90E-12 | 3.58E-11 | 16.02737875 |
| LRRC7 | -1.103544954 | 5.901495626 | -6.998755037 | 8.16E-12 | 3.69E-11 | 15.99568101 |
| PCDHA1 | -1.084328516 | 7.212645184 | -6.993121139 | 8.47E-12 | 3.82E-11 | 15.95981189 |
| SECTM1 | 1.04678133 | 5.309363545 | 6.980083017 | 9.22E-12 | 4.14E-11 | 15.87689393 |
| E2F2 | 1.244169541 | 5.247886708 | 6.971060899 | 9.77E-12 | 4.39E-11 | 15.8195911 |
| LOC148145 | -1.415312992 | 4.758378364 | -6.969887191 | 9.85E-12 | 4.42E-11 | 15.81214093 |
| IGFBP3 | 1.259566289 | 9.355363418 | 6.94586822 | 1.15E-11 | 5.14E-11 | 15.65990701 |
| IL7R | 1.002964419 | 2.127978997 | 6.942459567 | 1.18E-11 | 5.25E-11 | 15.63833791 |
| FAM163A | -1.206328782 | 2.467169281 | -6.93750194 | 1.21E-11 | 5.41E-11 | 15.60698291 |
| CLEC4GP1 | -1.298871302 | 2.604207496 | -6.933994524 | 1.24E-11 | 5.53E-11 | 15.5848111 |
| RXFP1 | -1.44831365 | 3.277097767 | -6.931838026 | 1.26E-11 | 5.60E-11 | 15.5711836 |
| CHRNA7 | -1.03776276 | 3.722814789 | -6.928133086 | 1.29E-11 | 5.73E-11 | 15.54777925 |
| HLA-DRB6 | 1.379875296 | 6.116323081 | 6.925908148 | 1.31E-11 | 5.81E-11 | 15.53372915 |
| CHD5 | -1.421103963 | 8.471337378 | -6.918344752 | 1.37E-11 | 6.09E-11 | 15.48599551 |
| PGF | -1.18022483 | 8.488743921 | -6.912371651 | 1.43E-11 | 6.32E-11 | 15.44832896 |
| FGF17 | -1.276676177 | 5.081490767 | -6.909209259 | 1.46E-11 | 6.44E-11 | 15.42839772 |
| SP8 | 1.377468983 | 1.962634205 | 6.906125751 | 1.49E-11 | 6.57E-11 | 15.40897094 |
| LOC84856 | -1.30968129 | 6.99029826 | -6.900970917 | 1.54E-11 | 6.78E-11 | 15.37651037 |
| SSPO | -1.003629871 | 6.870637472 | -6.897564527 | 1.57E-11 | 6.92E-11 | 15.35507098 |
| KCNK5 | 1.131727315 | 2.090233368 | 6.89011455 | 1.65E-11 | 7.25E-11 | 15.30821231 |
| TMEM132D | -1.741528276 | 5.562194069 | -6.888855294 | 1.66E-11 | 7.30E-11 | 15.30029603 |
| CD163 | 1.622311682 | 7.768833224 | 6.878870242 | 1.77E-11 | 7.77E-11 | 15.23756768 |
| OMD | -1.003601815 | 4.458529676 | -6.865683205 | 1.93E-11 | 8.42E-11 | 15.15483941 |
| UGT8 | -1.003374148 | 9.908518759 | -6.860285244 | 2.00E-11 | 8.70E-11 | 15.12101356 |
| FPR1 | 1.088367898 | 7.754414815 | 6.84169389 | 2.25E-11 | 9.73E-11 | 15.00468159 |
| SLC24A4 | -1.240038376 | 7.367434942 | -6.841129701 | 2.26E-11 | 9.77E-11 | 15.00115538 |
| RCC1 | 1.077386931 | 7.030908129 | 6.839808303 | 2.28E-11 | 9.85E-11 | 14.99289753 |
| FKBP5 | 1.114665814 | 9.869109918 | 6.828614054 | 2.45E-11 | 1.05E-10 | 14.92299417 |
| CDK1 | 1.112614077 | 6.693239162 | 6.810914054 | 2.74E-11 | 1.18E-10 | 14.81265954 |
| RSPO3 | -1.25496263 | 3.891758454 | -6.799116833 | 2.95E-11 | 1.26E-10 | 14.73925286 |
| CYP4X1 | -1.053485241 | 4.861699119 | -6.793289884 | 3.06E-11 | 1.31E-10 | 14.70303453 |
| SLC17A6 | -1.647791825 | 4.315412664 | -6.793084637 | 3.07E-11 | 1.31E-10 | 14.70175926 |
| PTPRN | -1.437390356 | 9.696271153 | -6.792393897 | 3.08E-11 | 1.32E-10 | 14.69746768 |
| BEST3 | -1.096762511 | 8.891481749 | -6.789271788 | 3.14E-11 | 1.34E-10 | 14.6780745 |
| CPLX3 | -1.40976566 | 4.82183883 | -6.787295102 | 3.18E-11 | 1.36E-10 | 14.66580003 |
| KCNA4 | -1.18309867 | 4.084701067 | -6.785206321 | 3.22E-11 | 1.37E-10 | 14.65283273 |
| TEAD4 | 1.001298695 | 5.285488825 | 6.778519557 | 3.36E-11 | 1.43E-10 | 14.6113432 |
| KRT7 | 1.053125843 | 1.756526447 | 6.776714041 | 3.40E-11 | 1.45E-10 | 14.60014632 |
| ANGPT2 | 1.047633992 | 7.118659206 | 6.773336842 | 3.48E-11 | 1.48E-10 | 14.57920935 |
| SEMA3A | 1.212185148 | 5.731504828 | 6.77188699 | 3.51E-11 | 1.49E-10 | 14.57022365 |
| BCL2A1 | 1.147495637 | 4.360191033 | 6.763509873 | 3.70E-11 | 1.57E-10 | 14.51833652 |
| HOXA11AS | 1.293564137 | 1.444737237 | 6.761142118 | 3.76E-11 | 1.59E-10 | 14.50368057 |
| HMMR | 1.036942298 | 5.005016948 | 6.744866879 | 4.16E-11 | 1.75E-10 | 14.40305591 |
| ROPN1L | 1.046574867 | 2.136807054 | 6.740582212 | 4.28E-11 | 1.80E-10 | 14.37659886 |
| GPR6 | -1.211967672 | 2.134833187 | -6.735168613 | 4.43E-11 | 1.86E-10 | 14.34319094 |
| CIDEA | -1.065479281 | 1.792640542 | -6.715774441 | 5.00E-11 | 2.09E-10 | 14.22369163 |
| EFHC2 | 1.142131134 | 4.389535444 | 6.711699111 | 5.13E-11 | 2.15E-10 | 14.1986177 |
| SYN2 | -1.546398056 | 9.131141947 | -6.706219952 | 5.31E-11 | 2.22E-10 | 14.16492662 |
| PBK | 1.387873091 | 6.087758651 | 6.690383713 | 5.87E-11 | 2.44E-10 | 14.06767994 |
| PAX1 | -1.647848978 | 4.086014716 | -6.688583033 | 5.94E-11 | 2.46E-10 | 14.05663458 |
| PIRT | 1.393753655 | 6.578938852 | 6.683584281 | 6.13E-11 | 2.54E-10 | 14.02598531 |
| GLS2 | -1.152607397 | 5.388163948 | -6.682408888 | 6.17E-11 | 2.56E-10 | 14.01878132 |
| CACNA1E | -1.041644283 | 7.980102337 | -6.674164519 | 6.50E-11 | 2.68E-10 | 13.96828135 |
| HOXD3 | 1.584335057 | 3.188910657 | 6.669726445 | 6.68E-11 | 2.75E-10 | 13.94111807 |
| SKA3 | 1.022878987 | 5.181576894 | 6.668119237 | 6.75E-11 | 2.78E-10 | 13.93128487 |
| ZDHHC23 | 1.133569368 | 5.922702231 | 6.633206278 | 8.40E-11 | 3.43E-10 | 13.71817202 |
| DTL | 1.113490062 | 6.123645771 | 6.631646315 | 8.48E-11 | 3.47E-10 | 13.70867175 |
| CALY | -1.639300867 | 6.547607061 | -6.623833908 | 8.90E-11 | 3.63E-10 | 13.661122 |
| GRIN2B | -1.263619513 | 3.378667065 | -6.616729381 | 9.30E-11 | 3.79E-10 | 13.61792165 |
| CCKBR | -1.469795391 | 4.635129411 | -6.595150535 | 1.06E-10 | 4.32E-10 | 13.48694681 |
| H19 | 1.799937039 | 4.518865999 | 6.575964354 | 1.20E-10 | 4.83E-10 | 13.37079713 |
| RGS7BP | -1.07929091 | 7.055942276 | -6.568061097 | 1.26E-10 | 5.07E-10 | 13.32303519 |
| TLE6 | -1.003937594 | 5.540693153 | -6.56772861 | 1.26E-10 | 5.08E-10 | 13.32102692 |
| HILS1 | 1.220938272 | 1.731222293 | 6.562058802 | 1.31E-10 | 5.25E-10 | 13.28679374 |
| ANKRD55 | -1.148066165 | 3.835741265 | -6.543156864 | 1.47E-10 | 5.88E-10 | 13.17284781 |
| CNTN6 | -1.25512923 | 4.636114691 | -6.54161644 | 1.48E-10 | 5.94E-10 | 13.16357396 |
| VEPH1 | -1.762348638 | 6.195985747 | -6.535468172 | 1.54E-10 | 6.16E-10 | 13.12657774 |
| HTR5A | -1.470097125 | 3.90925332 | -6.534911378 | 1.54E-10 | 6.18E-10 | 13.12322877 |
| CASC5 | 1.262456733 | 4.904707576 | 6.528227159 | 1.61E-10 | 6.42E-10 | 13.08304379 |
| NDST3 | -1.319315763 | 4.877753692 | -6.526803011 | 1.62E-10 | 6.47E-10 | 13.07448642 |
| SPATA18 | 1.046680212 | 4.068191526 | 6.525551271 | 1.64E-10 | 6.52E-10 | 13.06696631 |
| SLC35F3 | -1.132879507 | 4.940023696 | -6.514048674 | 1.76E-10 | 6.98E-10 | 12.99791898 |
| PVALB | -1.696745939 | 3.577696173 | -6.509749725 | 1.80E-10 | 7.16E-10 | 12.97213986 |
| NTS | 1.133334094 | 1.081636279 | 6.494581711 | 1.98E-10 | 7.83E-10 | 12.88129825 |
| MTUS2 | -1.124680585 | 4.865489872 | -6.49354669 | 1.99E-10 | 7.88E-10 | 12.87510602 |
| OLFM4 | -1.163308939 | 4.368407028 | -6.492680245 | 2.00E-10 | 7.92E-10 | 12.86992297 |
| ACCN1 | -1.318790305 | 5.542160817 | -6.492012883 | 2.01E-10 | 7.95E-10 | 12.86593124 |
| TSHR | -1.62691093 | 5.916879134 | -6.485893402 | 2.09E-10 | 8.23E-10 | 12.82934456 |
| PITX1 | 1.280632409 | 2.706519336 | 6.485654798 | 2.09E-10 | 8.25E-10 | 12.82791861 |
| SNAP25 | -1.423647288 | 11.44221201 | -6.484401628 | 2.11E-10 | 8.30E-10 | 12.82043009 |
| SYNGR3 | -1.209781228 | 7.774416668 | -6.469129094 | 2.31E-10 | 9.09E-10 | 12.72926517 |
| LOC134466 | -1.176899054 | 6.360802431 | -6.454809841 | 2.52E-10 | 9.90E-10 | 12.64395613 |
| VSTM2L | -1.100964149 | 7.959650617 | -6.428844009 | 2.96E-10 | 1.15E-09 | 12.48967012 |
| CLEC2L | -1.465970058 | 5.264673205 | -6.419129767 | 3.14E-10 | 1.22E-09 | 12.43208497 |
| CACNG3 | -1.702025903 | 5.901601284 | -6.417596462 | 3.17E-10 | 1.23E-09 | 12.42300244 |
| KIFC1 | 1.134136229 | 6.665668304 | 6.413614763 | 3.24E-10 | 1.26E-09 | 12.39942546 |
| VCAM1 | 1.252213316 | 9.016673322 | 6.405557235 | 3.41E-10 | 1.32E-09 | 12.35175218 |
| C5orf49 | 1.184292413 | 4.556171871 | 6.398132721 | 3.56E-10 | 1.38E-09 | 12.30786933 |
| CDSN | 1.015875438 | 2.453348436 | 6.395831352 | 3.61E-10 | 1.39E-09 | 12.29427579 |
| CLVS1 | -1.208596551 | 6.169842369 | -6.388916935 | 3.77E-10 | 1.45E-09 | 12.25345934 |
| MKI67 | 1.314549469 | 7.929914844 | 6.363978595 | 4.38E-10 | 1.67E-09 | 12.10655829 |
| EPHA6 | -1.108253527 | 2.983509641 | -6.355724317 | 4.61E-10 | 1.76E-09 | 12.05804372 |
| MARVELD3 | -1.305138647 | 5.982518766 | -6.351097788 | 4.74E-10 | 1.80E-09 | 12.03087474 |
| TCERG1L | -1.257065006 | 3.591302715 | -6.349434229 | 4.78E-10 | 1.82E-09 | 12.02110973 |
| COL17A1 | -1.002682173 | 2.936617413 | -6.342026939 | 5.00E-10 | 1.90E-09 | 11.97765578 |
| PPP2R2C | -1.125768736 | 9.791815582 | -6.327087494 | 5.47E-10 | 2.07E-09 | 11.89014706 |
| PTPRR | -1.230619242 | 5.107081852 | -6.324220512 | 5.57E-10 | 2.10E-09 | 11.87337369 |
| ZMAT4 | -1.183867087 | 4.932987367 | -6.308198385 | 6.13E-10 | 2.30E-09 | 11.77975526 |
| HCN1 | -1.531055014 | 5.408433787 | -6.298036495 | 6.51E-10 | 2.44E-09 | 11.72048381 |
| NPY2R | 1.192189763 | 3.180480583 | 6.290637828 | 6.81E-10 | 2.55E-09 | 11.67738086 |
| MSX2 | -1.352372302 | 4.856532824 | -6.281299314 | 7.20E-10 | 2.69E-09 | 11.62303868 |
| F2RL1 | 1.034077341 | 5.948747987 | 6.27171426 | 7.62E-10 | 2.84E-09 | 11.5673337 |
| FOXD3 | 1.316513003 | 2.694864403 | 6.269184781 | 7.74E-10 | 2.88E-09 | 11.5526454 |
| CCDC129 | 1.152412759 | 1.378672205 | 6.256375601 | 8.35E-10 | 3.10E-09 | 11.47834237 |
| HS3ST2 | -1.2003329 | 6.502622436 | -6.255725509 | 8.38E-10 | 3.11E-09 | 11.47457481 |
| INMT | 1.121527204 | 4.318076948 | 6.255069647 | 8.42E-10 | 3.12E-09 | 11.47077415 |
| SDC4 | 1.107465303 | 9.490282133 | 6.240453637 | 9.18E-10 | 3.39E-09 | 11.38616437 |
| RALYL | -1.044975042 | 6.591255383 | -6.233883682 | 9.54E-10 | 3.52E-09 | 11.34818722 |
| GABRA1 | -1.727405795 | 7.188355568 | -6.215268273 | 1.07E-09 | 3.92E-09 | 11.24076864 |
| DLEC1 | 1.049458573 | 5.874108068 | 6.209172267 | 1.10E-09 | 4.06E-09 | 11.20565212 |
| LRAT | 1.136962944 | 5.677688826 | 6.208333479 | 1.11E-09 | 4.07E-09 | 11.20082253 |
| LILRB5 | -1.028555775 | 3.905603393 | -6.19797126 | 1.18E-09 | 4.32E-09 | 11.141205 |
| FNDC1 | 1.141092951 | 3.0625598 | 6.173304497 | 1.37E-09 | 4.96E-09 | 10.99963282 |
| C1orf168 | -1.071879853 | 2.844960397 | -6.167117932 | 1.42E-09 | 5.14E-09 | 10.96420189 |
| STC1 | 1.161071624 | 3.678989227 | 6.157024522 | 1.50E-09 | 5.44E-09 | 10.90646179 |
| PKMYT1 | 1.004779694 | 6.261962431 | 6.140441652 | 1.66E-09 | 5.98E-09 | 10.8117751 |
| TMEM130 | -1.337643444 | 8.993048893 | -6.121917965 | 1.85E-09 | 6.63E-09 | 10.70626678 |
| C18orf34 | -1.140898779 | 4.280832608 | -6.116182073 | 1.91E-09 | 6.84E-09 | 10.67365169 |
| PTGER3 | -1.132828838 | 3.891324675 | -6.098405439 | 2.12E-09 | 7.55E-09 | 10.57273883 |
| NEUROD6 | -1.561258448 | 3.732212327 | -6.091122413 | 2.21E-09 | 7.86E-09 | 10.53146843 |
| LMX1A | -1.286881759 | 1.858531147 | -6.088358708 | 2.25E-09 | 7.98E-09 | 10.5158186 |
| GLT1D1 | -1.147939901 | 5.944166943 | -6.088123589 | 2.25E-09 | 7.99E-09 | 10.5144875 |
| FPR3 | 1.185973985 | 5.574087636 | 6.087324008 | 2.26E-09 | 8.03E-09 | 10.50996107 |
| C10orf105 | 1.119593178 | 5.898162308 | 6.082864933 | 2.32E-09 | 8.23E-09 | 10.48472765 |
| MSLN | 1.184320635 | 3.547749185 | 6.056941153 | 2.69E-09 | 9.53E-09 | 10.33834448 |
| GRM2 | -1.028279708 | 5.271038239 | -6.053581346 | 2.75E-09 | 9.70E-09 | 10.31941233 |
| TNFSF13B | 1.104833263 | 5.283971911 | 6.01877402 | 3.36E-09 | 1.18E-08 | 10.12381208 |
| SLC7A4 | -1.015494024 | 3.983406367 | -6.012710503 | 3.48E-09 | 1.22E-08 | 10.08983803 |
| GJA3 | 1.170956752 | 2.120110225 | 5.976679721 | 4.28E-09 | 1.49E-08 | 9.888569431 |
| ANO3 | -1.09782429 | 5.64096144 | -5.965175446 | 4.57E-09 | 1.59E-08 | 9.824527595 |
| DRD5 | -1.008119281 | 2.426429501 | -5.950169009 | 4.98E-09 | 1.72E-08 | 9.741151169 |
| MAL2 | -1.454192488 | 5.877185186 | -5.944679913 | 5.14E-09 | 1.77E-08 | 9.710699127 |
| GRM5 | -1.139437434 | 8.049252025 | -5.940017674 | 5.28E-09 | 1.82E-08 | 9.684853465 |
| TFAP2B | 1.046251412 | 0.966363256 | 5.921746428 | 5.86E-09 | 2.01E-08 | 9.583734818 |
| PHYHIP | -1.119606358 | 9.90238349 | -5.919522182 | 5.93E-09 | 2.03E-08 | 9.571443682 |
| MPPED1 | -1.430794028 | 5.853030966 | -5.914032497 | 6.12E-09 | 2.10E-08 | 9.541125005 |
| COL6A3 | 1.092782544 | 5.606459502 | 5.913612563 | 6.14E-09 | 2.10E-08 | 9.538806784 |
| COL4A6 | 1.077825325 | 5.413326286 | 5.906334983 | 6.39E-09 | 2.19E-08 | 9.498654109 |
| LRRC2 | 1.062406141 | 5.470711513 | 5.903565255 | 6.50E-09 | 2.22E-08 | 9.483383975 |
| UNC13C | -1.468363665 | 5.905722851 | -5.900449078 | 6.61E-09 | 2.26E-08 | 9.466211257 |
| ESM1 | 1.238987915 | 2.848237629 | 5.895500464 | 6.80E-09 | 2.32E-08 | 9.438956528 |
| RGS1 | 1.109671679 | 9.15268404 | 5.873290925 | 7.71E-09 | 2.61E-08 | 9.316882067 |
| CCK | -1.529064299 | 6.865471995 | -5.872987419 | 7.72E-09 | 2.62E-08 | 9.315216635 |
| GNG13 | -1.073529606 | 2.362052189 | -5.864318626 | 8.11E-09 | 2.74E-08 | 9.267679967 |
| COL22A1 | 1.552246269 | 5.744179639 | 5.859393704 | 8.34E-09 | 2.82E-08 | 9.240700705 |
| CPNE7 | -1.193222556 | 4.840966776 | -5.853531528 | 8.62E-09 | 2.91E-08 | 9.20861287 |
| CDH7 | -1.158637861 | 3.344448718 | -5.825380062 | 1.01E-08 | 3.38E-08 | 9.054911112 |
| HCN4 | -1.02841406 | 4.813503694 | -5.806789112 | 1.12E-08 | 3.74E-08 | 8.953763356 |
| ICAM5 | -1.243632449 | 6.755301266 | -5.785729286 | 1.26E-08 | 4.18E-08 | 8.839525199 |
| CPNE9 | -1.152184914 | 3.916136331 | -5.77360631 | 1.35E-08 | 4.47E-08 | 8.77392957 |
| YSK4 | 1.285166316 | 2.670003268 | 5.759106614 | 1.46E-08 | 4.82E-08 | 8.695632321 |
| FAM81B | 1.181166874 | 1.658744873 | 5.7562399 | 1.49E-08 | 4.89E-08 | 8.680172735 |
| CAMK4 | -1.158571561 | 5.444953338 | -5.729719141 | 1.72E-08 | 5.62E-08 | 8.537472294 |
| PCDHGA3 | -1.025789031 | 6.315425598 | -5.728005428 | 1.74E-08 | 5.67E-08 | 8.528271211 |
| KLK7 | -1.329625101 | 2.951021493 | -5.71848522 | 1.83E-08 | 5.97E-08 | 8.477200393 |
| FXYD7 | -1.209446025 | 7.122507788 | -5.704609863 | 1.98E-08 | 6.43E-08 | 8.402900287 |
| FBN3 | 1.171885063 | 5.118678995 | 5.694689113 | 2.09E-08 | 6.78E-08 | 8.349873748 |
| ZNF676 | -1.261374634 | 3.285258696 | -5.682312226 | 2.24E-08 | 7.23E-08 | 8.283833002 |
| IL6 | 1.044366961 | 2.8458947 | 5.67954078 | 2.27E-08 | 7.34E-08 | 8.269062425 |
| STC2 | -1.006884429 | 7.629254936 | -5.678566829 | 2.28E-08 | 7.37E-08 | 8.263873199 |
| STYK1 | -1.284783248 | 3.566213923 | -5.671357205 | 2.38E-08 | 7.66E-08 | 8.225484622 |
| ANKRD34C | -1.025898548 | 2.449684262 | -5.665615271 | 2.45E-08 | 7.89E-08 | 8.19494168 |
| KCNE1L | 1.174742753 | 4.811819072 | 5.649396799 | 2.68E-08 | 8.60E-08 | 8.108818408 |
| PRKCG | -1.350002412 | 6.737623171 | -5.633503368 | 2.92E-08 | 9.33E-08 | 8.024632385 |
| NEFM | -1.501290618 | 8.173837468 | -5.627286579 | 3.02E-08 | 9.64E-08 | 7.991759566 |
| OPRK1 | -1.167695963 | 3.739127538 | -5.62214476 | 3.11E-08 | 9.90E-08 | 7.964595125 |
| NEURL | -1.07076679 | 7.295016765 | -5.609423975 | 3.33E-08 | 1.06E-07 | 7.897484927 |
| TEKT1 | 1.119072992 | 3.042364584 | 5.601200336 | 3.48E-08 | 1.10E-07 | 7.854171479 |
| SH2D5 | -1.112377271 | 5.87661738 | -5.585467033 | 3.80E-08 | 1.20E-07 | 7.77146157 |
| GABRB2 | -1.390027857 | 7.231359326 | -5.577179624 | 3.97E-08 | 1.25E-07 | 7.727977408 |
| CCL2 | 1.054003494 | 7.608624275 | 5.548047192 | 4.65E-08 | 1.45E-07 | 7.575572608 |
| SLITRK4 | -1.046775198 | 5.158077274 | -5.538086098 | 4.90E-08 | 1.53E-07 | 7.523623829 |
| GJB2 | 1.132366696 | 4.400233197 | 5.526270288 | 5.22E-08 | 1.62E-07 | 7.462109662 |
| SLC6A17 | -1.24042342 | 8.349672058 | -5.454735849 | 7.66E-08 | 2.34E-07 | 7.092185563 |
| CRH | -1.000284711 | 2.588158727 | -5.454463223 | 7.67E-08 | 2.34E-07 | 7.090783931 |
| SCARA5 | -1.113540615 | 3.588334797 | -5.393322407 | 1.06E-07 | 3.19E-07 | 6.778020992 |
| HOXD8 | 1.460549469 | 3.94752647 | 5.386722854 | 1.10E-07 | 3.30E-07 | 6.744449164 |
| SLC17A8 | -1.42041555 | 4.96603461 | -5.384599151 | 1.11E-07 | 3.34E-07 | 6.733653716 |
| VIP | -1.105215485 | 4.003011873 | -5.37766151 | 1.15E-07 | 3.46E-07 | 6.698413974 |
| LOC154822 | -1.561870935 | 8.629162422 | -5.357717409 | 1.28E-07 | 3.82E-07 | 6.597333773 |
| GPX3 | -1.010966131 | 11.26750206 | -5.351918514 | 1.32E-07 | 3.94E-07 | 6.568006887 |
| NR0B1 | -1.043757055 | 4.495505138 | -5.335581627 | 1.43E-07 | 4.27E-07 | 6.485538537 |
| ADAM6 | 1.345050725 | 4.943888911 | 5.321601173 | 1.54E-07 | 4.58E-07 | 6.415144382 |
| SCN7A | 1.236409453 | 4.300003747 | 5.309762148 | 1.64E-07 | 4.85E-07 | 6.35566189 |
| KCNJ4 | -1.043575422 | 6.888059539 | -5.277215988 | 1.94E-07 | 5.70E-07 | 6.192752302 |
| CTXN3 | -1.26323643 | 2.339584168 | -5.267200594 | 2.05E-07 | 5.98E-07 | 6.142800867 |
| SLC12A5 | -1.24315023 | 8.176707375 | -5.264643574 | 2.07E-07 | 6.06E-07 | 6.130061451 |
| NELL1 | -1.275173543 | 5.325213719 | -5.250061356 | 2.24E-07 | 6.51E-07 | 6.057516885 |
| SOHLH1 | -1.188421897 | 3.477501761 | -5.231356243 | 2.46E-07 | 7.13E-07 | 5.964725789 |
| NTSR2 | -1.159555432 | 8.413249447 | -5.224927725 | 2.54E-07 | 7.36E-07 | 5.932904264 |
| PACSIN1 | -1.398763842 | 8.775730011 | -5.222385507 | 2.58E-07 | 7.45E-07 | 5.920329837 |
| GABRA5 | -1.344066811 | 6.304945446 | -5.211745802 | 2.72E-07 | 7.85E-07 | 5.867762915 |
| STMN2 | -1.296874283 | 9.747079733 | -5.206211289 | 2.80E-07 | 8.06E-07 | 5.840456978 |
| SDR16C5 | -1.038160789 | 2.728786121 | -5.183514058 | 3.14E-07 | 9.01E-07 | 5.728747188 |
| SLC6A7 | -1.442130908 | 4.599982521 | -5.145481573 | 3.81E-07 | 1.08E-06 | 5.542546003 |
| KIAA1239 | -1.211156455 | 3.597260289 | -5.14457549 | 3.83E-07 | 1.09E-06 | 5.538125017 |
| SCGN | -1.114850265 | 2.223726303 | -5.134409001 | 4.03E-07 | 1.14E-06 | 5.488568529 |
| A2BP1 | -1.113472117 | 7.390819316 | -5.117201294 | 4.40E-07 | 1.24E-06 | 5.404891038 |
| TMEM155 | -1.21019294 | 5.28428054 | -5.091729412 | 5.00E-07 | 1.40E-06 | 5.281491839 |
| SULF1 | 1.002575962 | 8.693560342 | 5.075988005 | 5.41E-07 | 1.51E-06 | 5.205510145 |
| NEUROD2 | -1.249187349 | 5.772400987 | -5.064618611 | 5.73E-07 | 1.60E-06 | 5.150763783 |
| SLC6A15 | -1.097064651 | 6.191081974 | -5.057582434 | 5.93E-07 | 1.65E-06 | 5.11693848 |
| WNT10B | -1.035621017 | 4.647834191 | -5.056655601 | 5.96E-07 | 1.66E-06 | 5.112486051 |
| OLFM3 | -1.1192899 | 5.113375625 | -5.029484599 | 6.82E-07 | 1.89E-06 | 4.982286693 |
| SHISA6 | -1.131884745 | 5.19554967 | -5.015426552 | 7.32E-07 | 2.02E-06 | 4.915171878 |
| ARSF | 1.140463913 | 4.175662769 | 4.992169944 | 8.21E-07 | 2.25E-06 | 4.804515648 |
| SULT4A1 | -1.268578424 | 7.929497308 | -4.968520815 | 9.22E-07 | 2.52E-06 | 4.692469949 |
| FSTL4 | -1.035966539 | 5.712708514 | -4.919631715 | 1.17E-06 | 3.17E-06 | 4.462372889 |
| SST | -1.086829516 | 6.112529808 | -4.912551451 | 1.21E-06 | 3.27E-06 | 4.429220951 |
| NPTX1 | -1.068981615 | 9.226937858 | -4.91218686 | 1.22E-06 | 3.28E-06 | 4.427515001 |
| DMRTC1 | -1.153751603 | 2.722455745 | -4.907577423 | 1.24E-06 | 3.35E-06 | 4.405957009 |
| IGFBPL1 | -1.052508506 | 4.238851653 | -4.90730554 | 1.24E-06 | 3.35E-06 | 4.40468601 |
| MCHR2 | -1.108664224 | 2.13342821 | -4.891247849 | 1.35E-06 | 3.61E-06 | 4.329732957 |
| GPR22 | -1.096399072 | 4.080680246 | -4.875241181 | 1.45E-06 | 3.89E-06 | 4.255240594 |
| MMP7 | 1.003932609 | 2.125963487 | 4.874539175 | 1.46E-06 | 3.90E-06 | 4.251978661 |
| GABRA4 | -1.277669591 | 6.070914861 | -4.868609998 | 1.50E-06 | 4.01E-06 | 4.224445307 |
| SCN9A | 1.000139656 | 4.07765172 | 4.855903342 | 1.60E-06 | 4.25E-06 | 4.165542137 |
| SYNPR | -1.20761627 | 6.646226696 | -4.83365119 | 1.78E-06 | 4.71E-06 | 4.062727739 |
| FRMPD4 | -1.07722633 | 5.192825314 | -4.831081598 | 1.80E-06 | 4.76E-06 | 4.050882865 |
| LOC285780 | -1.06966675 | 2.662445416 | -4.824881756 | 1.85E-06 | 4.90E-06 | 4.022327529 |
| SV2B | -1.242205062 | 7.672594499 | -4.811526803 | 1.98E-06 | 5.21E-06 | 3.960930737 |
| LEFTY2 | 1.221666982 | 4.390302721 | 4.785999401 | 2.23E-06 | 5.85E-06 | 3.844005864 |
| SLC32A1 | -1.20587557 | 5.802646122 | -4.78516372 | 2.24E-06 | 5.87E-06 | 3.840187743 |
| GJB6 | -1.443583834 | 5.36990272 | -4.775374306 | 2.35E-06 | 6.13E-06 | 3.795506518 |
| PRMT8 | -1.065352708 | 5.553570387 | -4.760442206 | 2.52E-06 | 6.55E-06 | 3.727513957 |
| CREG2 | -1.226919269 | 6.059301786 | -4.739906043 | 2.78E-06 | 7.18E-06 | 3.634321745 |
| KCNH7 | -1.040988496 | 3.720275971 | -4.733206364 | 2.87E-06 | 7.41E-06 | 3.603998662 |
| GAD2 | -1.179044475 | 5.234239211 | -4.714170187 | 3.14E-06 | 8.06E-06 | 3.518054324 |
| KRT17 | -1.054606981 | 3.253492704 | -4.712912275 | 3.15E-06 | 8.11E-06 | 3.512386293 |
| VSNL1 | -1.315398871 | 8.760507963 | -4.683873707 | 3.61E-06 | 9.24E-06 | 3.381926688 |
| ADAMDEC1 | 1.020745124 | 2.067093876 | 4.634929339 | 4.54E-06 | 1.15E-05 | 3.163712375 |
| GPR26 | -1.357268212 | 4.452351814 | -4.629062084 | 4.67E-06 | 1.18E-05 | 3.137695039 |
| RYR2 | -1.126223114 | 6.354854672 | -4.620293847 | 4.86E-06 | 1.23E-05 | 3.09887025 |
| SLC17A7 | -1.428555339 | 9.52155221 | -4.59941007 | 5.35E-06 | 1.35E-05 | 3.006671795 |
| CAMK2A | -1.260271252 | 9.730580364 | -4.599265698 | 5.35E-06 | 1.35E-05 | 3.006035754 |
| NGB | -1.117936808 | 3.728594508 | -4.547195629 | 6.80E-06 | 1.69E-05 | 2.777836316 |
| PTPN5 | -1.045628376 | 7.428427575 | -4.516933537 | 7.80E-06 | 1.93E-05 | 2.646311808 |
| KCNV1 | -1.248499404 | 3.715377242 | -4.49349485 | 8.68E-06 | 2.13E-05 | 2.54500003 |
| C1QL3 | -1.171840143 | 4.478122919 | -4.404402528 | 1.29E-05 | 3.12E-05 | 2.164353429 |
| TAC1 | -1.138667202 | 5.070264838 | -4.293275772 | 2.11E-05 | 4.95E-05 | 1.699473329 |
| TNNT2 | -1.027856101 | 2.564649004 | -4.180318519 | 3.43E-05 | 7.86E-05 | 1.238267794 |
| COL11A1 | 1.02034882 | 7.478560423 | 4.17878987 | 3.45E-05 | 7.91E-05 | 1.232104953 |
| TBR1 | -1.023181787 | 5.101312961 | -4.0739341 | 5.36E-05 | 0.000119886 | 0.814403246 |
| NEFL | -1.216819026 | 8.683723729 | -4.070539291 | 5.44E-05 | 0.000121471 | 0.801045769 |
| NRGN | -1.041921443 | 9.830275255 | -4.017795132 | 6.76E-05 | 0.000149235 | 0.594855713 |
| RASAL1 | -1.007647569 | 5.655675641 | -3.956146758 | 8.70E-05 | 0.000189469 | 0.357057019 |
| SLC30A3 | -1.083484079 | 5.297810432 | -3.902517714 | 0.000107964 | 0.000232875 | 0.153004351 |
| psiTPTE22 | -1.100583993 | 7.1171698 | -3.816158327 | 0.000152181 | 0.000322423 | -0.17006636 |
| ECEL1 | -1.002952526 | 3.608004726 | -3.707502545 | 0.000232244 | 0.00048137 | -0.56684127 |
